# IMGT-NC Engineered Variants of INN Therapeutic IG or Antibodies and Related IgSF Proteins (TR, FPIA and CPCA): Bridging Sequences, Structures and Functions for AI

**DOI:** 10.3390/biom16091264

**Published:** 2026-09-01

**Authors:** Marie-Paule Lefranc

**Affiliations:** 1 IMGT^®^, the International ImMunoGeneTics Information System^®^ (IMGT), Laboratoire d’ImmunoGénétique Moléculaire (LIGM), Institut de Génétique Humaine (IGH), Université de Montpellier (UM), Centre National de la Recherche Scientifique (CNRS), UMR 9002 CNRS-UM, 141 Rue de la Cardonille, CEDEX 5, 34396 Montpellier, France; marie-paule.lefranc@umontpellier.fr; 2 Institut Universitaire de France, 75231 Paris, France

**Keywords:** antibody-dependent cellular cytotoxicity (ADCC), antibody-dependent cellular phagocytosis (ADCP), complement-dependent cytotoxicity (CDC), composite protein for clinical applications (CPCA), fusion protein for immune applications (FPIA), ImMunoGeneTics (IMGT), immunoglobulin (IG), immunoglobulin superfamily (IgSF), T-cell receptor (TR)

## Abstract

IMGT^®^, the international information system^®^ (IMGT), was created in 1989 by Marie-Paule Lefranc (Université de Montpellier and CNRS) in Montpellier, France, to deal with and to manage the huge diversity of immunoglobulins (IG) or antibodies and T-cell receptors (TR), which are the antigen receptors (AR) of the adaptive immune response (AIR) of jawed vertebrates. The founding of IMGT^®^ marked the advent of immunoinformatics, a new science which emerged at the interface between immunogenetics and bioinformatics. The biocuration of the IMGT data (IG and TR sequences, genes and structures) and the implementation of the IMGT system (7 databases, 17 tools, 25,000 Web resources pages) are based on the IMGT Scientific chart rules (keywords, labels, nomenclature, numbering…) generated from the IMGT-ONTOLOGY axioms and concepts. The IMGT nomenclature (IMGT-NC) and the IMGT unique numbering, the two pillars of immunoinformatics, have been used to define 335 engineered variants for effector properties and formats of therapeutic antibodies (including 12 chimerisotypes) and TR, fusion proteins for immune applications (FPIA) and composite proteins for clinical applications (CPCA). IMGT-NC engineered variant names from the World Health Organization (WHO) International Nonproprietary Name (INN) programme descriptions contribute to the common language for immunoinformatics and artificial intelligence (AI).

## 1. Introduction

Immunogenetics is a science that studies the genetics of the immune system and immune responses. Among them, the adaptive immune response (AIR), acquired by jawed vertebrates (or *gnathostomata*) about 450 million years ago, is characterized by an extreme diversity of the specific antigen receptors (AR) that comprise the immunoglobulins (IG) or antibodies [1] and the T-cell receptors (TR) [2]. The IG occur as membrane IG (mIG) monomers (two light chains, kappa (IGK) or lambda (IGL) and two heavy chains (IGH)), anchored by their heavy chains in the surface membrane of the B cells, associated with the signaling CD79A and CD79 B coreceptors, which together form the B-cell receptor (BcR) [1] or as secreted IG (sIG) by the plasma cells [1]. The TR occur as membrane monomers (two chains, alpha (TRA) and beta (TRB), or gamma (TRG) and delta (TRD)), anchored by their two chains in the surface membrane of the T cells, associated with the signaling dimeric CD3D_CD3E, CD3G_CD3E and CD247_CD247 coreceptors, which together form the T-cell receptor (TcR) [2]. The potential expressed AR repertoire of each individual is estimated to comprise about 2 × 10^12^ different IG and TR, and the limiting factor is only the number of B and T cells that an organism is genetically programmed to produce [3]. This huge diversity results from the complex and unique molecular synthesis and genetics of the IG and TR chains [1,2] that include (i) DNA molecular rearrangements (combinatorial diversity) in multiple loci, three loci for IG (IGH at 14q32.33, IGK at 2p11.2, IGL at 22q11.2 [1,4]) and four loci for TR (TRA and TRD at 14q11.2, TRB at 7q34, TRG at 7p14 [2,5]) in humans, located on different chromosomes (four in humans, chr 2, 7, 14 and 22); (ii) nucleotide deletions and insertions at the rearrangement junctions (junctional diversity, mostly represented by the N-diversity) [1,2]; and (iii) for the IG, somatic hypermutations (SHM) and class switch recombinations (CSR) [1,6,7,8].

Owing to the complexity and diversity of the IG and TR immune-expressed repertoires and to their implications in fundamental and medical research, immunogenetics represents one of the greatest challenges for data interpretation. Extensive biological expertise, considerable effort in standardization and elaboration of an efficient system for the management of related knowledge were required. To answer that challenge, IMGT^®^, the international ImMunoGeneTics information system^®^ (IMGT) [1,2,3,9,10,11,12,13], was created in 1989 by Marie-Paule Lefranc (Laboratoire d’ImmunoGénétique Moléculaire (LIGM) founded by Gérard Lefranc, Université de Montpellier (UM) and Centre National de la Recherche Scientifique (CNRS), Montpellier, France).

The founding of IMGT^®^ marked the advent of immunoinformatics [3], a novel science that emerged at the interface between immunogenetics and bioinformatics [9,10,11,12,13]. Immunoinformatics has been built on IMGT-ONTOLOGY [1,2,14,15,16,17,18,19,20,21,22,23] and its seven axioms: IDENTIFICATION [24], DESCRIPTION [25], CLASSIFICATION [26], NUMEROTATION [27,28,29,30,31,32,33,34], ORIENTATION [21], LOCALIZATION [21], OBTENTION [14,21]. These postulate that any object and process and any relation have to be identified, described, classified, numbered, localized and orientated, and that the way it is obtained can be characterized.

Each IMGT-ONTOLOGY axiom has generated concepts, which have themselves generated the IMGT Scientific chart rules (examples are shown between parentheses): (i) concepts of identification (IMGT standardized keywords) [24], (ii) concepts of description (IMGT standardized labels in capital letters) [25], (iii) concepts of classification [26] (IMGT nomenclature or IMGT-NC [1,2,4,5,26,35,36,37,38,39], for IG and TR loci [1,2,40], for genes and alleles [1,2,39], for gene copy number variations (CNV) [39,40], for amino acids [41], for allotypes [42,43,44], for IGHG engineered variants involved in antibody effector properties, half-life, physicochemical properties and structure [45,46]), (iv) concepts of numerotation [27,28,29,30,31,32,33,34] (IMGT unique numbering for variable (V) domain of IG, TR and immunoglobulin superfamily (IgSF) proteins other than IG and TR [29], IMGT unique numbering for constant (C) domain of IG, TR and IgSF proteins other than IG and TR [30], IMGT unique numbering for groove (G) domain of major histocompatibility (MH) class I (MH1), class II (MH2) and MH superfamily (MhSF) proteins other than MH [31]; IMGT Collier de Perles ‘on one layer’ and ‘on two layers’ [47,48,49,50,51,52]; IMGT complementarity determining region (CDR-IMGT) and IMGT framework region (FR-IMGT) lengths [53,54,55,56,57,58,59]; paratope, pMH contacts sites, contact analysis [54,55,60,61,62]), (v) concepts of orientation (IMGT locus orientation forward ‘FWD’ or reverse ‘REV’ on chromosome [1,2,40], gene in direct or opposite orientation of transcription in locus [1,2,39,40]), (vi) concepts of localization (IMGT gene order from 5′ to 3′ in the locus, haplotypes [39,40]), and (vii) concepts of obtention (source, metadata) [24].

The IMGT nomenclature and the IMGT unique numbering, created for immunogenetics data, are the two pillars of immunoinformatics [3,38], on which the IMGT^®^ databases, tools and Web resources pages have been built for 35 years [63,64,65,66,67,68,69,70,71]. These two breakthroughs are the foundations which have made IMGT^®^ the global reference in immunogenetics and immunoinformatics [1,2,38,39,40]. IMGT^®^ data focused mainly on the genes, sequences and structures of the IG or antibodies and TR, from any species from fish to humans, and relations based on IMGT-ONTOLOGY. IMGT^®^ data are particularly useful in scientific basic and medical research, for the study of the IG and TR gene and locus evolution of the adaptive immune response, the analysis of the diversity and clonality of the IG and TR repertoires, and the development of antibody engineering for diagnostic and therapeutic applications. IMGT^®^ biocurated data include, as examples, (i) IG and TR genes and alleles in databases (nucleotide (nt-) sequences and translation in IMGT/LIGM-DB [72,73,74,75,76], IG and TR gene and allele names in IMGT/GENE-DB [77], amino acid (aa-) sequences in IMGT/2Dstructure-DB and IMGT/3Dstructure-DB [53,56,57]), (ii) sequence reference directories in analysis tools (nt-sequences in IMGT/V-QUEST and IMGT/HighV-QUEST [70], domain aa-sequences in IMGT/DomainGapAlign [56,58,59]), (iii) Chromosomal localizations, Locus representations [1,2], Gene tables [4,5], Alignments of alleles [1,2], Allotypes [43,44], Protein displays [1,2], IMGT Colliers de Perles [34], etc., in the IMGT Repertoire for IG and TR [71], for MH, and for related proteins of the immune system (RPI).

Created in 2010, IMGT/mAb-DB [71,78,79,80] is a query interface for therapeutic monoclonal antibodies (IG, mAb), TR, fusion proteins for immune applications (FPIA), composite proteins for clinical applications (CPCA) and related proteins of the immune system (RPI). The aa-sequences, from the World Health Organisation (WHO) International Nonproprietary Name (INN) programme lists [81,82,83], are biocurated in IMGT/2Dstructure-DB (INN number code) [53,56,57] and used as two-dimensional (2D) reference aa-sequences (with a receptor description per chain and domains) for a given INN. If a three-dimensional (3D) structure is available in the Research Collaboratory for Structural Bioinformatics Protein Data Bank (RCSB PDB) [84], a link from IMGT/mAb-DB provides access to the biocurated aa-sequences in IMGT/3Dstructure-DB (PDB code) [53,56,57] and to the specific data related to 3D structures (contact analysis, paratope, epitope, hydrogen bonds in IMGT Collier de Perles on two layers). In IMGT/2Dstructure-DB and in IMGT/3Dstructure-DB [53,56,57], the aa-sequences are displayed assigned, per domain, to the closest gene(s) and allele(s), by comparison with the IMGT reference directory using the internal IMGT/DomainGapAlign tool [56,58,59]. Experimental analysis of binding sites [85,86,87], effector properties [88,89,90,91,92,93,94] and half-life [95] have led to advances in the engineering of the antibody Fc with an increasing number of variants. Systematic analysis of the varied designs of therapeutic antibodies and FPIA assigned INN and systematic analysis of the Fc aa changes in therapeutic antibodies and FPIA designed to reduce or to enhance binding to Fc-gamma receptors have been performed [96,97,98,99]. In order to bridge the gap at the amino acid level, between aa-sequences and properties and function types, the IMGT^®^ nomenclature of IGHG engineered variants using the IMGT numbering has been implemented [45,46], with the Eu-IMGT positions aligned to the IMGT numbering (rather than to the Eu aa-sequence itself [100]). The Eu-IMGT positions permit the creation of additional positions not present in the Eu protein and avoid the original Eu aa-sequence errors.

Here, we first review, in three sections, the IMGT nomenclature (IMGT-NC) and IMGT unique numbering breakthroughs, the two pillars of immunoinformatics; the IMGT IGHG and IGHA reference aa-sequences, IMGT/DomainGapAlign tool, IMGT/3Dstructure-DB and IMGT/2Dstructure-DB, for variants aa data and sequence analysis; the IMGT-NC engineered IGHG variant classification in four categories and in 18 types, each defined by a ‘Property and Function Type’ [45,46]. In the core fourth section, we report, in an IMGT-formatted Supplementary Table S1, the IMGT-NC and description of 335 IMGT engineered variants. In the following fifth section, we report among them (in Supplementary Table S2), the 228 IMGT engineered variant names identified in the WHO INN sequences [81,82,83], up to recommended list R95 and proposed list L134, included. The twelve chimerisotype variants (of which six are found in INN) are detailed in the sixth section. The properties and functions of the IMGT-NC engineered variants are extracted from humanly retrieved bibliographical references [45,46] and, thus, contribute with those assigned to the INN therapeutic antibodies [81,82,83], to the common language of immunogenetics and immunoinformatics in antibody engineering for artificial intelligence (AI).

## 2. IMGT Nomenclature (IMGT-NC) and IMGT Unique Numbering Breakthroughs

### 2.1. IMGT Nomenclature (IMGT-NC) Breakthrough

The first breakthrough, in June 1989 at the Human Gene Mapping (HGM)10 Workshop at New Haven, was to make the variable (V), diversity (D), and joining (J) DNA, which participate in the synthesis of the IG or TR chain, officially recognized as ‘genes’, as well as the conventional genes, allowing them to be entered for the first time into the HGM gene database [3]. This led to the standardized IMGT nomenclature (IMGT-NC) for IG and TR, based on locus, group, subgroup, gene and allele [1,2,35,36,37,38,39,40].

The nucleotide sequences of the IG and TR genes, identified in the publications and in the generalist sequence databases (European Molecular Biology Laboratory (EMBL) (now European Nucleotide Archive (ENA))/GenBank/DNA Database of Japan (DDBJ)) were biocurated by the IMGT team at LIGM [39] and entered into IMGT/LIGM-DB [72,73,74,75,76], created in 1994. This IMGT nucleotide sequence database uses the same accession numbers ‘GEDI’ (for GenBank_EMBL_DDBJ_IMGT/LIGM-DB) for data interoperability, and for LinkOut at NCBI. Following assignment of an IMGT reference sequence to each IG or TR gene or allele, the IMGT gene names were then entered into HGNC in 1999 [1,2,4,5,39] and in IMGT/GENE-DB [77], the IMGT gene database created in 2003. IMGT/GENE-DB proposes an interface to query the IMGT/LIGM-DB sequences of the genes, per label (nucleotides and translation), thus bridging genes and sequences.

### 2.2. IMGT Numbering Breakthrough

The second breakthrough in 1997 was to consider the variable (V) and constant (C) domains of the IG and TR as evolutionary related structural units [27,28,29,30] (despite their fundamental differences in sequence (V-(D)-J region versus C-region exon, CDR-IMGT versus loops) and in number and type of strands (‘9 antiparallel strands’ for the V domain versus ‘7 antiparallel strands and a transverse CD strand’ for the C domain) [3]. This led to the standardized IMGT unique numbering for V domain [29] and for C domain [30] (with four common amino acids, cysteines C23 and C104 of the disulfide bridge, tryptophane W41, hydrophobic 89) and their IMGT Collier de Perles graphical representation (47, 48, 49, 50, 51, 52). The IMGT unique numbering for V and C domains of IG and TR [29,30] has been extended to the V-like and C-like domains of the immunoglobulin superfamily (IgSF) proteins other than IG and TR [32,33,34]. The IMGT unique numbering defined for the groove (G) domains of major histocompatibility MH1 and MH2 proteins [31] has been extended to the G-like domain of the MH superfamily (MhSF) proteins other than MH1 and MH2 (represented only by MH1-like as there is no known MH2-like) [32,33,34].

## 3. IMGT IGHG and IGHA Reference aa-Sequences, IMGT/DomainGapAlign Tool, IMGT/3Dstructure-DB and IMGT/2Dstructure-DB

### 3.1. IMGT IGHG and IGHA Reference aa-Sequences

The constant region of the IG and TR defines the isotype of the expressed IG and TR chains. In humans, there are nine classical isotypes for the IGH locus, H-mu, H-delta, H-gamma3, H-gamma1, H-alpha1, H-gamma2, H-gamma4, H-epsilon, and H-alpha2 chains, the constant region of the IGH chains being encoded by the IGHM, IGHD, IGHG3, IGHG1, IGHA1, IGHG2, IGHG4, IGHE and IGHA2 genes, respectively, whereas the variable domain VH is encoded by the rearranged V-D-J-region [1,8]. For the light chains, there are one isotype (L-kappa chain) for the IGK locus, the constant region being encoded by one IGKC gene, and several isotypes (L-lambda chains) for the IGL locus depending on the number of functional IGLC genes (e.g., C-lambda2 encoded by IGLC2), respectively, while the variable VL domain (either V-kappa or V-lambda) is encoded by the rearranged V-J-region [1,8].

**Figure 1:**
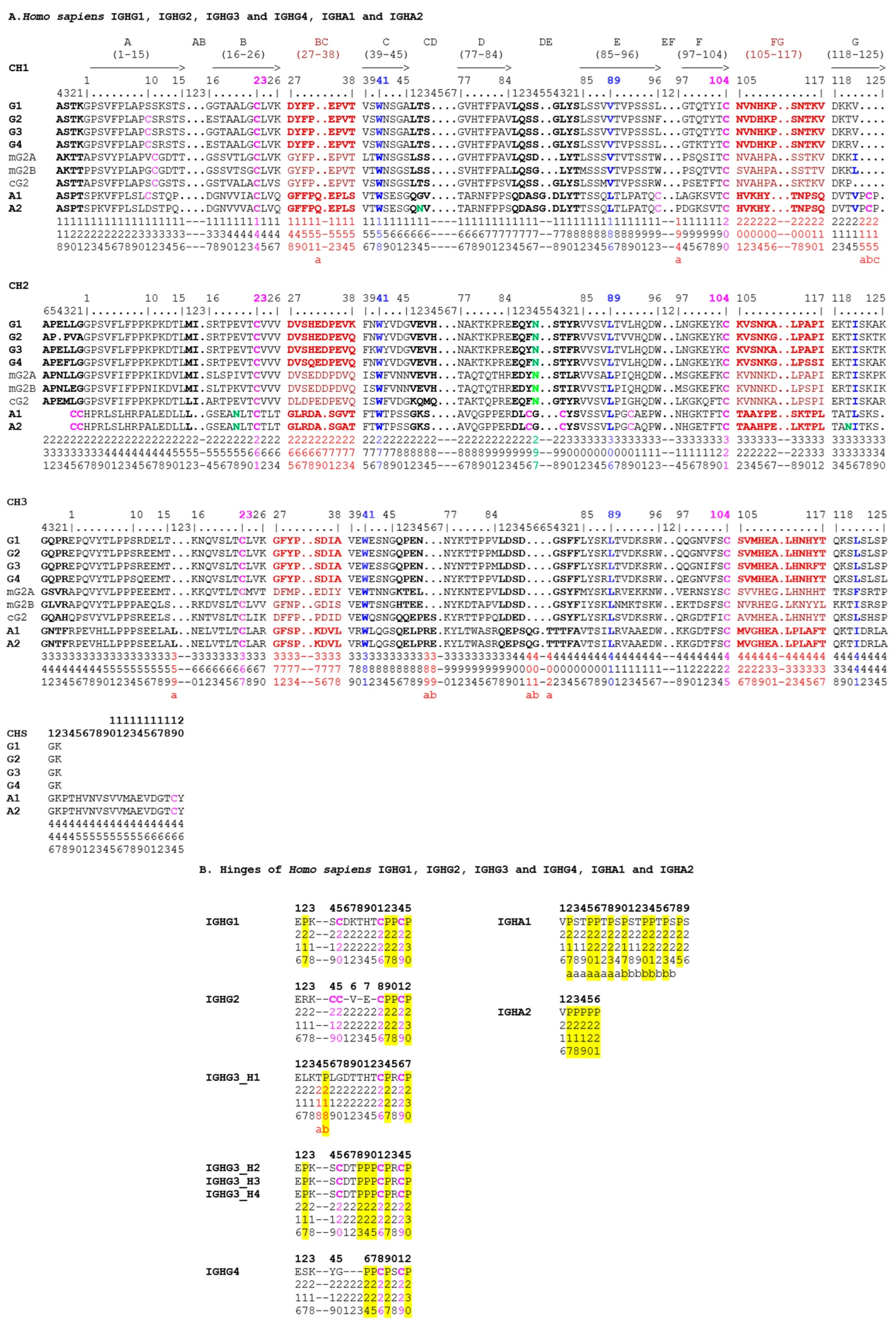
Protein display of the *Homo sapiens* IGHG1, IGHG2, IGHG3, IGHG4, IGHA1 and IGHA2 [1,45,46]. (**A**). Protein display of the CH1, CH2, CH3 and CHS of the *Homo sapiens* IGHG1, IGHG2, IGHG3 and IGHG4, IGHA1 and IGHA2. The IMGT reference aa-sequences displayed are *Homo sapiens* (Homsap) IGHG1*01 (J00228), IGHG2*01 (J00230), IGHG3*01 (X03604), IGHG4*01 (K01316) [1,45,46] (G1, G2, G3 and G4 in Figure 1A), IGHA1*01 (J00220), IGHA2*01 (J00221) [1] (A1 and A2 in Figure 1A), *Mus musculus* (Musmus) IGHG2A*01 (V00825) and IGHG2B*01 (V00763) (mG2A, mG2B in Figure 1A), *Canis lupus familiaris* (Canlupfam) IGHG2*01 (IMGT000001) (cG2 in Figure 1A). The Eu-IMGT positions are read vertically in the three lines below the aa-sequences alignments. The delimitation of the domains (aa start-end) are IGHG1 CH1 1.4–121 which correspond to Eu-positions ‘118’ (read vertically ‘1’ (top line), ‘1’ (middle line)’ and ‘8’ (bottom line)) and ‘215’ (read vertically ‘2’, ‘1’ and ‘5’), CH2 1.6–125 which correspond to Eu-positions 231–340 and CH3 1.4–125 which correspond to the Eu-positions 341–445 [45,46]. (**B**). Protein display of the hinge exons of the IGHG1, IGHG2, IGHG3 (_H1, _H2,_H3 and _H4) and IGHG4 (each aligned to Eu-IMGT positions 216–230) and of the hinge region of IGHA1 (19 aa) and IGHA2 (6 aa) fused in 5′ of the CH2. Proline (P) are highlighted in yellow and cysteines (C) are in pink. Additional Eu-IMGT positions are shown in red and with letters a, b, c. (With permission from M-P. Lefranc and G. Lefranc, LIGM, Founders and Authors of IMGT^®^, the international ImMunoGeneTics information system^®^, https://www.imgt.org).

The basic IMGT molecular immmunogenetics information necessary for the variant characterization [45,46] comprises the following:

(i) The IMGT classes of the 20 common amino acids for the ‘hydropathy’, ‘volume’, ‘chemical characteristics’ properties [41];

(ii) The IMGT Colliers de Perles (‘On one layer’ and ‘On two layers’) of the CH1, CH2 and CH3 domains encoded by the four *Homo sapiens* IGHG genes (IGHG1 [45,46], IGHG2, IGHG3 and IGHG4) [46];

(iii) The structural features of the C domain template and those of the CH1, CH2 and CH3 of the four *Homo sapiens* IGHG genes [46], based on the IMGT unique numbering for C domain [30];

(iv) The correspondence between the IMGT unique numbering of the CH1, CH2 and CH3 of *Homo sapiens* IGHG1*01 (J00228) [1,30] and the Eu-IMGT positions, shown in table [46] or in Protein display (Figure 1);

(v) The IMGT Protein display of the CH1, CH2 and CH3 domains of the IMGT reference aa-sequences, with the Eu-IMGT positions [46] (Figure 1A), which includes the translation of:

- *Homo sapiens* (Homsap) IGHG1*01 (J00228), IGHG2*01 (J00230), IGHG3*01 (X03604), IGHG4*01 (K01316) (G1, G2, G3 and G4) [1,45,46], IGHA1*01 (J00220), IGHA2*01 (J00221) (A1 and A2) [1];
- *Mus musculus* (Musmus) IGHG2A*01 (V00825) and IGHG2B*01 (V00763);
- *Canis lupus familiaris* (Canlupfam) IGHG2*01 (IMGT000001);

(vi) The hinge regions of the *Homo sapiens* IGHG1 (15 aa), IGHG2 (12 aa); IGHG3 (H1 (17 aa), H2, H3 and H4 (15 aa)), IGHG4 (12 aa) encoded by the hinge exons, and those of the *Homo sapiens* IGHA1 (19 aa) and IGHA2 (6 aa) fused in 5′ of the respective CH2 (Figure 1B);

(vii) The display of the Homsap IGKC, and IGLC2 with Eu-IMGT positions (IGKC 1.4–126, 108–214; IGLC2 1.5–128, 107–215) [45].

### 3.2. IMGT/DomainGapAlign Tool

IMGT/DomainGapAlign [56,58,59] is the tool for the analysis of amino acid sequences per domain, widely used for the analysis of the V domain and C domain aa-sequences of therapeutic antibodies. In the V domain analysis, IMGT/DomainGapAlign is used for the identification of the closest V and J gene(s) and allele(s), the N-and-D-region delimitation, CDR-IMGT and FR-IMGT delimitations and lengths (shown with the number of aa, separated by dots, between brackets, e.g., [8.8.13]), the identification of aa-changes due to SHM or to engineered variants, and the evaluation of the V domain humanization. In the C domain analysis, IMGT/DomainGapAlign is used for the identification of the closest C domain(s) of C gene(s) and allele(s), the description of polymorphisms with aa-changes, allotypes and engineered variants. The IMGT/DomainGapAlign tool interface and documentation are available online (‘Analyse your sequence using IMGT domains’, https://www.imgt.org/3Dstructure-DB/cgi/DomainGapAlign.cgi (accessed on 12 July 2026); IMGT/DomainGapAlign Documentation, https://www.imgt.org/3Dstructure-DB/doc/IMGTDomainGapAlign.shtml (accessed on 12 July 2026)). IMGT/DomainGapAlign is the tool used in the assignment of the IMGT-NC names to IGHG engineered variants [45,46], done by comparison to the allele *01 of the closest IGHC gene. For a given receptor, the assignment is completed for each chain, individually (e.g., H, L, H”, L’” chains in INN descriptions or H, L, M, N chains in IMGT/2Dstructure-DB).

### 3.3. IMGT/3Dstructure-DB and IMGT/2Dstructure-DB

IMGT/3Dstructure-DB [53,56,57] has been implemented for the management of aa-sequences from the 3D structures from RCSB PDB (using the PDB code as ID). The aa-sequences of 3D structures (PDB code) are frequently used for the molecular identification of an antigen receptor; however, these sequences may be partial and/or modified for the experimental structural analysis.

IMGT/2Dstructure-DB [53,56,57] is the IMGT protein database, built with the same computational framework and interface as IMGT/3Dstructure-DB. IMGT/2Dstructure-DB is an archive for the Kabat amino acid sequences (336 ‘KAB’ entries), which do not have nucleotide sequences in IMGT/LIGM-DB, and in the generalist nucleotide databases. IMGT/2Dstructure-DB has become the IMGT reference database for the aa-sequences of therapeutic proteins which include antigen receptors (IG or antibodies, TR), fusion proteins for immune applications (FPIA) and composite proteins for clinical applications (CPCA). Thus, IMGT/2Dstructure-DB contains therapeutic aa-sequences from the WHO INN programme, published biannually in the proposed (P) and recommended (R) lists. The ID protein entries are the INN numbers, with the permission of WHO INN [81,82,83].

For each receptor entry, IMGT/2Dstructure-DB displays, per chain and per domain, the aa-sequence analyzed by the internal IMGT/DomainGapAlign tool. Amino acid differences, compared to the aa-sequence of the domain of the IMGT closest reference sequence, are highlighted in the IMGT/2Dstructure-DB sequences (in orange), in the IMGT Collier de Perles [48,49,50,51,52] and in the displayed results of IMGT/DomainGapAlign [56,58,59].

## 4. IMGT-NC Engineered IGHG Variant Classification in Four Categories and in 18 Types

The constant region of the IG or antibody heavy gamma (IGHG) chain is frequently engineered to modify the effector properties (antibody-dependent cellular cytotoxicity (ADCC), anti-dependent cellular phagocytosis (ADCP) and complement-dependent cytotoxicity (CDC)), the half-life, the physicochemical properties and/or the structure of the therapeutic monoclonal antibodies [45,46].

The standardized IMGT^®^ nomenclature (IMGT-NC) of engineered IGHG variants [45,46] has been set up for an easier comparison between engineered antibody variants described in publications and in INN descriptions. The IMGT-NC of the IGHG variants comprises 18 types (from 1 to 18), each type being defined by a ‘Property and Function Type‘ [45,46] (Table 1). These types have been attached to the four categories of ‘effector’, ‘half-life’, ‘physicochemical’ and ‘structure’.

The ‘effector’ category comprises eight types (1 to 8): 1. ADCC reduction, 2. ADCC enhancement, 3. ADCC and ADCP enhancement, 4. CDC enhancement, 5. CDC reduction, 6. ADCC and CDC reduction, 7. FcγRIIB binding increase and B-cell inhibition (coengagement of antigen and FcγR on the same cell), 8. knock out CH2 84.4 glycosylation (ADCC reduction).

Two categories comprise a single type: the ‘half-life’ category includes type 9 (half-life increase or decrease), whereas the ‘physicochemical’ category includes type 10 (abrogation of binding to Protein A, thermal stability, pI, reduced acid-induced aggregation, novel binding).

The ‘structure’ category comprises eight types (11 to 18): 11. formation of additional bridge for domain stabilization, including scFv, 12. prevention of IgG4 half-IG exchange, amino acid changes or insertion at the elbow of crossovers (e.g., aa change at the elbow of VH-C-kappa, insertion upstream of G4 in VK-G4 CH1), 13. hexamerization, 14. enhancement of heterodimerization H-H of bispecific antibodies (knobs-into-holes, charge steering, additional disulfide bridge), 15. suppression of inter H-L and/or inter H-H disulfide bridges, 16. site-specific drug attachment, e.g., additional cysteine, 17. enhancement of heteropairing H-L of bispecific antibodies, 18. control of H chain expression or of half-IG exchange of bispecific IgG by amino acid changes [45,46].

## 5. IMGT^®^ Nomenclature (IMGT-NC) and Description of 335 Engineered Variants

The IMGT® nomenclature (IMGT-NC) of IGHG engineered variants involved in effector properties (ADCC, ADCP, CDC), half-life, physicochemical properties and structure of therapeutic monoclonal antibodies, FPIA and CPCA [45,46] has been built on the analysis of published experimental data. Table 2 provides a list of the engineered variants, which were first analyzed [101,102,103,104,105,106,107,108,109,110,111,112,113,114,115,116,117,118,119,120,121,122,123,124,125,126,127,128,129,130,131,132,133,134,135,136,137,138,139,140,141,142,143,144,145,146,147,148,149,150,151,152,153,154,155,156,157,158,159,160,161,162,163,164,165,166,167,168,169,170,171,172,173,174,175,176,177,178,179,180,181,182,183,184,185,186,187,188,189,190], for their effector properties and/or for the structure formats of the IG. The analysis of these variants has been used to define the standardized IMGT-NC rules for the description of the engineered variants of therapeutic IG or antibodies and related IgSF proteins (TR, FPIA and CPCA) [45,46], reported in Table S1.

The IMGT^®^ nomenclature (IMGT-NC) reported in Table S1 comprises the name and description of 335 engineered variants, with their characterization [45,46], classically displayed in ten columns which comprise the following:

(1) The IMGT variant identifier (ID) (column 1).

(2) The IMGT engineered variant name: it includes the ‘Species’ (column 2) (genus and species in the 6-letter (Homsap for *Homo sapiens*, Musmus *for Mus musculus*) or 9-letter (Canlupfam for *Canis lupus familiaris*) abbreviation, and the ‘Variant name’ (column 3) made of the variant type(s) (number(s) from 1 to 18), the gene name abbreviation (e.g., G1 for IGHG1), the letter ‘v’ (for variant) with a number (e.g., Homsap 1-G1v1, Homsap 6-G1v14), followed if needed with a hyphen and a number (e.g., 6-G1v14-48) [45,46].

(3) The IMGT engineered variant definition (column 4): it includes, for each engineered amino acid (aa) change, the domain (e.g., CH1, CH2 or CH3) or the region (hinge, CHS), the amino acid is in the one-letter abbreviation [41] with its position according to the IMGT unique numbering for C domain [30], e.g., Homsap 1-G1v1, CH2 P1.4, Homsap 6-G1v14, CH2 A1.3, A1.2, A114. In addition to the very common domains and regions mentioned above, the VH, CH1, V-KAPPA, C-KAPPA, C-LAMBDA2 domains are found in type 17 variants, the IMGT unique numbering for V domains [29] being used for VH and V-KAPPA. Type 10 variants have been described for C-KAPPA, C-LAMBDA2 and C-LAMBDA3, the variant (ID690) 9-chain-v1 has been described for JCHAIN and the variant (ID139) 10-G1v73-3 for a region ‘linker’.

(4) The list of the IMGT aa change(s) per domain (column 5): it includes for each aa change, the aa before the change (in bold, red), the symbol ‘>’ and the aa after the change (in bold, green) followed by the Eu-IMGT position between parentheses (e.g., CH2 **P**114 > **A** (329)). Alias variant names found in the literature (e.g., LALA, YTE, etc.) are written in blue.

(5) The Eu-IMGT positions of the aa changes (column 6) (e.g., P329A).

(6) The IMGT topological motifs [45,46] (column 7) identifiable in gene and domain with positions according to the IMGT unique numbering for C-domain [30], followed between parentheses, by the Eu-IMGT positions (the display of the motif shows the aa involved in the change highlighted in bold, red before the change and green after the change, respectively (e.g., IGHG1 CH2 1.6–3 AP**E**LLGGPS > AP**P**LLGGPS; underlined amino acids in the motif correspond to additional positions in the IMGT unique numbering for the C-domain [30], e.g., AP**E**LLG and AP**P**LLG correspond to 1.6, 1.5, 1.4, 1.3, 1.2 and 1.1 positions, respectively. The hinge numbering [45,46] or the IMGT unique numbering for V domain [29] is used whenever it is relevant. The JCHAIN coding aa translation (NCBI, CCDS 3545.1) is used for the 9-Jchain-v1.

(7) ‘Property and function type’ (at least one, and up to three per variant) (columns 8, 9 and 10). In column 10 (the most-right column), information on 3D structure with PDB codes are indicated (if available in IMGT/3Dstructure-DB and RCSB PDB) [45,46]. The number in red indicates the number of WHO INN, up to lists R95 and PL134, included. The first (one or two) INN (name, number and, between parentheses, PL and RL numbers) are displayed in green italics. If more than 2, only the first one (chronological order in the list) is displayed. ‘0’ in black means no INN. In the variant description of the sequences of therapeutic antibodies of the WHO INN lists [81,82,83], the Eu-IMGT position is replaced by the position of the aa change in the INN antibody chain sequences.

Of the total of 335 engineered variants in Table S1, 323 are from *Homo sapiens* (Homsap) with 295 H-gamma (constant region encoded by a IGHG gene) chains. The isotype repartition is the following: two hundred eleven are ‘G1’ (ID 1–199, 700–711), thirty-one ‘G2’ (ID 201–231), five ‘G3’ (ID 301–305), forty-eight ‘G4’ (ID 401–448), eighteen are kappa (four ‘K’ with amino changes on C-kappa and V-kappa (ID 501–504), thirteen ‘KC’ with aa changes on C-kappa (ID 525–537), one ‘KV’ with aa changes on V-kappa (ID 570)), six lambda (five ‘LC2’ with aa changes on C-lambda2 (ID 575–579), one ‘LC3’ with aa change on C-lambda3 (ID 580)), four ‘scFv-‘ (ID 651–654) and one ‘Jchain-‘ (ID 690). The other eleven variants comprise six Canlupfam ‘G2’(ID 601–606) and five Musmus, of which one ‘G2A’ and four ‘G2B’(ID 646–650).

The great majority (310) of these 335 variants is defined by a single type. However, twenty-three variants are assigned to two types. This may result from the combination of amino acids of previously well-defined variants. A first example (ID 025) 6,5-G1v14-1-20 combines (ID 024) 6-G1v14-1 (CH2 A1.3, A1.2, A1), which reduces FcgammaR binding (ADCC reduction) and C1q binding (CDC reduction), and (ID 040) 5-G1v20 (CH2 A105), which reduces C1q binding (CDC reduction). A second example (ID 097) 11,8-G1v54-30 combines (ID 095) 11-G1v54, which stabilizes CH2 in the absence of N84.4 glycosylation [145], and (ID 054) 8-G1v30, which reduces FcgammaR binding (ADCC reduction) owing to the absence of N-glycosylation at CH2 N84.4 [129,130]. Other variants may be assigned to two types directly based on experimental data; an example is (ID 014) 2,7-G1v7 D3, E117 [108]. Two variants are assigned to three types: the first one (ID 016) 2,5,7-G1v8 increases FcgammaRIIIA affinity (ADCC enhancement, type 2), reduces C1q binding (CDC reduction, type 5) and has low binding to inhibitory FcgammaRIIB (type 7) [108]; the second one (ID 038) 4,10,13-G1v18 present in IgG1-005-RGY [116] includes 10,13-G1v98 (CH3 R1), which favors IgG1 hexomerization and increases C1q binding and CDC enhancement (type 4) [116].

The reported total of 335 variants represents unique outcomes with a unique IMGT-NC variant ID. The associations of engineered variants described in the literature with a given name, but whose variants are managed independently in IMGT-NC, are identified by a special ID code x9x* (‘9’ in second position and with an asterisk) (e.g., ID 497*) (Table S1), which is not accounted for in the total of 335 engineered variants. The list includes ten of these codes, where five are G1 (ID 795* to 799*), one G2 (ID 299*) and four G4 (ID 496* to 499*).

Two other special ID codes with ‘°’ or ‘°°’ for G1 indicate that the aa changes did not qualify as engineered variants; the first one, G1 CH1 A121 (ID 793°), is the result of a fortuitous experimental change without linked property or function, and the second one is an error in the sequence of *ofalimumab* in 3giz_H (ID 794°°) by comparison with the Fab sequence of *ofalimumab* in 6y9, which is the correct one. These last two are added to the table for information.

## 6. The 228 IMGT-NC Engineered Variants Identified in INN

Here, 228 IMGT-NC engineered variants from the list of the 335 variants (Table S1) were found assigned to INN, up to R95 and PL134 included. They are reported in Table S2. The four columns show the variant ID, the IMGT-NC engineered variant name, the number of INN per variant, and for each variant, the INN names and INN numbers with, between parentheses, the proposed list and the recommended list. ‘IG’ in black highlights IG fused with other proteins (-fusp suffix), an IG-isolated Fc with a modulating property (-imod suffix) and an IG fragment (-ciment suffix), where ‘CPCA’ and ‘FPIA’ are highlighted in black owing to the diversity of their suffixes [82]. The highlighted Fab (-mab suffix) is counted among the IG. List numbers in black correspond to amendments published in the corresponding INN lists.

## 7. Chimerisotype

### 7.1. Definition and Characterization of the Chimerisotypes

A chimerisotype defines a chimeric antibody chain made of amino acids of two (or more) different isotypes. Among the 294 variant names defined for the G1 to G4 isotypes, 12 chimerisotype variants have been identified and assigned to the isotype considered as the backbone of the chain, respectively, eight G1, three G2 and one G4. In addition to the classical engineered variant name [45,46], they are defined by the backbone isotype in italics, followed, between square brackets, by the number of aa changes of the other isotype(s) by comparison to the allele *01 of the backbone isotype. For example, the chimerisotype variant (ID 702) 4-G1v107, *G1[10aaG3]*, which characterizes G1G3 113F [172], corresponds to an IGHG1*01 backbone with ten G3 aa, four in CH2 (Q38, K40, F85.2 and T124) and six in CH3 (E12, M14, S44, N79, M84, I101). It shows an increase in C1q binding and CDC enhancement [172]. CH3 E12 and M14 are isoallotypic aa, present in IGHG1*03 and in G2, G3 and G4. CH3 102–125 (423–445) are from G1, with H115 (435) and Y116 (436) to keep binding to Protein A [172]. The other eleven chimerisotype variants comprise six identified in the INN and five for novel isotype effector functions or for heterodimerizations, described in the next sections.

### 7.2. Description of Six Different Chimerisotype Variants Found in INN

Six different chimerisotypes (two G1, three G2 and one G4 backbones, respectively) have been identified in a total of 13 INNs and are described below, based on the IGHG backbone.

#### 7.2.1. IGHG1 Backbone Chimerisotype

The chimerisotype variant (ID 015) 10-G1v7-1, *G1[8aaG2]* (Table S1) is present in two INNs, *talacotuzumab* 10508 and *tafasitamab* 10835 (Table S2). It corresponds to an IGHG1*01 backbone with eight G2 aa, five in CH2 (Q38, F84.3, F85.2, V92, T124) and three in CH3 (E12, M14, M84). As a result, and at the exception of a single aa CH2 A110 (conserved from IGHG1*01), the 10-G1v7-1 aa-sequence is, starting from CH2 Q38 and including the whole CH3, identical to that of IGHG2*01. 10-G1v7-1 is associated with (ID 014) 2,7-G1v7 CH2 D3, E117 (which confer the properties of ADCC enhancement by increase binding to FcγRIIIA, particularly the ‘lower-affinity’ FcγRIIIA F158 allele, and strong binding to inhibitory FcγRIIB [108]) to *talacotuzumab* and *tafasitamab* (ID 795*).

The chimerisotype variant (ID 707) 6-G1v112, *G1[12aaG4]* (Table S1), which characterizes one INN *cabotamig* 12632 (Table S2), corresponds to an IGHG1*01 backbone with twelve G4 aa, six in the CH2 (Q30, Q38, F84.3, G110, S115, S116) and six in the CH3 (Q11, E12, M14, R88, E98, L125), making the 6-G1v112 aa-sequence starting from CH2 Q30 and including the whole CH3 identical to those of IGHG4*01. CH3 E12 and M14 are isoallotypic aa present in IGHG1*03 and in G2, G3 and G4. The CH2 G110, S115 and S116 correspond to the (ID 114) 6-G1v60-1 variant and confer ADCC reduction and CDC reduction [136].

#### 7.2.2. IGHG2 Backbone Chimerisotype

The chimerisotype variant (ID 216) 15-G2v37-1, *G2[hG1S5]* (Table S1) of *abituzumab* 9509 (Table S2) corresponds to an IGHG2*01 backbone with a G1 hinge S5 (instead of a G2 hinge), leading to the absence of the disulfide bridge inter H-L. This variant is associated with (ID 214) 8,10-G2v36-7 (CH2 A84.3, Q84.4, with ADCC reduction owing to the absence of N-glycosylation at CH2 84.4 and elimination of a T-cell epitope generated by the Q84.4 aa change (ID299*).

The chimerisotype variant (ID 229) 6-G2v95, *G2[4aaG1,3aaG4]* (Table S1) in two INN, *nemolizumab* 10064 and *satralizumab* 10065 (Table S2), corresponds to an IGHG2*01 backbone with seven aa changes, where four are G1 (CH1 S10, K12, G16, G17) and three are G4 (CH2 Q30, CH3 Q11, E98). 6-G2v95 confers ADCC reduction and CDC reduction. The variant 6-G2v95 is associated in both antibodies with a G2 hinge S4 (ID215) 15-G2v37, leading to the absence of the disulfide bridge inter H-L, and in *satralizumab* with (ID 227) 9-G2v78-2 CH3 A114.

The chimerisotype variant (ID 228) 6-G2v94, *G2[11aaG4]* [179] (Table S1) characterizes six INN, *eculizumab* 8231, *samalizumab* 9307, *olendalizumab* 10037, *ravulizumab* 10659, *omoprubart* 12638 and *ascuprubart* 13837 (Table S2). 6-G2v94 corresponds to an IGHG2*01 backbone with eleven G4 aa, six in the CH2 (Q30, Y85.2, L92, S115, S116, A124) and five in the CH3 (Q11, V84, R88, E98, L125), making the aa-sequence starting from CH2 Q30 and including the whole CH3 identical to those of IGHG4*01. The CH2 Q30, L92, S115 and S116 correspond to the (ID 202) 6-G2v2 variant, which confers ADCC reduction and CDC reduction [176]. 6-G2v94 is associated in *ravulizumab* with (ID 212) 9-G2v24 CH3 L107, S114 and in *omoprubart* to (ID 211) 9-G2v21 CH2 Y15.1, T16, E18.

#### 7.2.3. IGHG4 Backbone Chimerisotype

The chimerisotype variant (ID 445) 10-G4v90 *G4[6aaG1]* (Table S1) of the INN *vixarelimab* 11350 (Table S2) corresponds to an IGHG4*01 backbone with six G1 aa in the CH3 (R11, D12, L14, K88, Q98, P125), making the CH3 identical to that of IGHG1*01. CH3 D12 and L14 are allotypic aa present in IGHG1*01. CH3 K88 (12-G4v6) reduces IgG4 half-IG exchange [141]. IGHG4 CH3 K88 is an allelic polymorphism present in Homsap IGHG4*03 (CH3 codon 69). The variant 10-G4v90 is associated in *vixarelimab* with (ID 408) 12-G4v5 G4 hinge P10, which prevents in vivo and in vitro IgG4 half-IG exchange [183], and with (ID 429) 8-G4v36 CH2 Q84.4, which confers ADCC reduction and CDC reduction owing to the absence of N-glycosylation at CH2 84.4 [129] (ID496*).

### 7.3. Chimerisotypes for Novel Isotype Effector Functions or for Heterodimerizations

Given the high percentage of identity between the H-gamma isotypes, a limited number of aa changes (up to 12 aa) characterize the chimerisotypes described, in the literature, as obtained by exchange of ‘exons’ (or ‘fragments of exons’). However, the number of aa changes is much higher when the isotype is from a different IG class and intended to confer effector properties of a novel class isotype to the original G1 backbone or to enhance heterodimerization of bispecific antibodies, as described below.

#### 7.3.1. Chimerisotypes with Different Class Isotypes for Novel Effector Properties

This is exemplified by the chimerisotype (ID 710) 3,4-G1A1v1, *G1-CH1hCH2[10aaA1]CH3[70aaA1]* [174] (Table 2), illustrated by the ‘cross-isotype’ trastuzumab-IgGA [174], in which the backbone is IGHG1*01 for the CH1-hinge-CH2 with ten IGHA1 aa in the CH2 and seventy IGHA1 aa in the CH3, conferring the effector properties of H-alpha1 of IgA1 (Table S1). The chimerisotype 3,4-G1A1v1 displays ADCC and ADCP enhancement and a high affinity to FcαRI on neutrophils and on macrophages [174]. It retains binding to FcγRI and FcγRIIA. It binds C1q with high affinity and exhibits a three-fold lower dissociation constant with C1q as compared to IgG1, which translates to improved CDC activity [174].

#### 7.3.2. Chimerisotypes with Amino Acid Domain Interface Exchange for the Generation of Fc Heterodimers and Bispecific Antibodies

Amino acid domain interface exchange is used for the generation of Fc heterodimers and bispecific antibodies. Thus, the chimerisotype variant (ID 708) 18-G1v113, *G1_AG SEED CH3[26aaA1]* [173] promotes, with the chimerisotype variant (ID 709) 18-G1v114, *G1_GA SEED CH3[34aaA1]* [173] (Table 2), the heteropairing H-H of bispecific G1 antibodies, by the complementary assymetric strand exchange engineered domain (SEED) composed of alternative IGHA1 and IGHG1 motifs (Table S1). The SEED CH3 heterodimerization is created by the exchange of beta-strands and loops between IgA and IgG1 CH3 homodimers [173].

#### 7.3.3. Chimerisotypes with TR Constant Domain Amino Acids for Heterodimerizations

The heavy chain heterodimerization of this type of chimerisotype is obtained by exchange of aa from the IgG1 CH3 homodimer interface with aa from the TR alpha/beta constant domain interface BEAT (bispecific engagement by antibodies based on the T cell receptor) [163]. The chimerisotype (ID 164) 18,10-G1v82-8, *G1[8aaTRAC,7aaG3,1aa°]* enhances the heteropairing of bispecific IgG1 with the chimerisotype (ID 165) 18,10-G1v82-9 *G1[11aaTRBC,3aa°]* on the other chain [163,164] (Table 2). The backbone of the chimerisotype 18,10-G1v82-8 is IGHG1*01 with eight aa from TRAC (A3, K20, V22, T26, S85.1, V86, W88, N90), seven aa from G3 (E12, M14, S44, M84, I101, R115, F116) and one aa of unspecified origin (Y79°) (Table S1). CH3 E12 and M14 are isoallotypic aa present in IGHG1*03 and in G2, G3 and G4. 18,10-G1v82-8 includes 10-G1v83 (CH3 R115, F116), which abrogates binding to Protein A [165]. The backbone of the chimerisotype 18,10-G1v82-9 is IGHG1*01 with eleven aa from TRBC and three aa of unspecified origin (L84°, E84.2° and A85.1°) [163,164].

## 8. Discussion

The IMGT-NC of the engineered variants provides, at the amino acid level, a standardized molecular description, which bridges sequences, structures and functions in the field of antibody engineering. The generation and production of bi- and multi-specific antibodies require improving biophysical properties [191], antibody scaffold to aid developability [152], the use of novel technologies and molecular formats [192,193,194,195], engineered amino acid changes for increasing heterodimerization H-H or heteropairing H-L [196,197,198,199]. The use of therapeutic antibodies requires understanding the variables which influence efficacity and safety, potency and tumor selectivity in clinical settings [200,201,202,203]. Advances in computational structure-based antibody design need standardized sequences and experimentally validated in silico antibody discovery design [204,205,206,207,208]. Language models, machine learning, large-scale datasets, and high-performance computing enhance antibody design for therapeutic development [209,210,211,212].

## 9. Conclusions

The major challenge of immunogenetics, from antigen receptor repertoires analysis, primary immunodeficiency diagnosis, minimal residual disease tracking, vaccine response studies and cancer immunogenetics to therapeutic antibody design, is to meet the highest standards to advance research, to provide accurate diagnostic information and to find the best and more appropriate therapeutics. The IMGT nomenclature and the IMGT unique numbering provide a rigorous, universal and biologically meaningful framework to identify, describe, classify and number the IG and TR genes, sequences and structures. They have made IMGT^®^, the international ImMunoGeneTics information system^®^, the global reference for immunoinformatics. The IMGT nomenclature is used for any IG and TR genes from *Homo sapiens* and from any other jawed vertebrate species [39], enabling comparative immunology, evolutionary immunogenetics, clinical, veterinary and zoonotic research, from fish to humans. The IMGT nomenclature [39] for IG and TR loci, genes and alleles, CNV and haplotypes, amino acids, allotypes and engineered variants, and the IMGT unique numbering for the V, C and G domains [32] provide the foundational, unifying infrastructure to immunogenetics and immunoinformatics [1,2,3]. They make the huge genetic diversity of the IG and TR antigen receptors and the specificity and effector properties of therapeutic antibodies, standardized, computable and comparable worldwide. Using the IMGT nomenclature and the IMGT unique numbering, researchers participate in a global scientific endeavor for AI in immunogenetics and immunoinformatics, in the field of antibody engineering with bispecific and multi-specific formats and novel engineered variants. In this context, speaking a common language, which bridges IG, TR and MH genes, sequences, structures and functions, ensures that we understand each other and communicate scientific findings effectively while also minimizing misunderstandings and confusion [37].

## 10. Availability and Citation

Online access to IMGT^®^ databases, tools and web resources is freely available for academics. Authors are encouraged to cite the references quoted in this article. Access to IMGT^®^ databases, tools and Web resources is under CNRS licenses and contracts for companies.

## Figures and Tables

**Table 1:** IMGT-NC engineered IGHG variant classification in four categories and 18 types, each type being defined by a ‘Property and Function Type’ [45,46] (with permission from M-P. Lefranc and G. Lefranc, LIGM, Founders and Authors of IMGT^®^, the international ImMunoGeneTics information system^®^, https://www.imgt.org).

IMGT-NC Variant Categories	IMGT-NC Variant Types	IMGT-NC Property and Function Type
Effector	1	antibody-dependent cellular cytotoxicity (ADCC) reduction.
	2	antibody-dependent cellular cytotoxicity (ADCC) enhancement.
	3	antibody-dependent cellular cytotoxicity (ADCC) and antibody-dependent cellular phagocytosis (ADCP) enhancement.
	4	complement-dependent cytotoxicity (CDC) enhancement.
	5	complement-dependent cytotoxicity (CDC) reduction.
	6	antibody-dependent cellular cytotoxicity (ADCC) and complement-dependent cytotoxicity (CDC) reduction.
	7	FcγRIIB binding increase and B cell inhibition (coengagement of antigen and FcγR on the same cell).
	8	knock out of the CH2 84.4 glycosylation (ADCC reduction).
Half-life	9	half-life increase or decrease.
Physicochemical	10	abrogation of binding to Protein A, thermal stability, pI, reduced acid-induced aggregation, novel binding.
Structure	11	additional intrachain disulfide bridge for domain or scFv stabilization.
	12	prevention of IgG4 half-IG exchange, amino acid changes or insertion at the elbow of crossovers.
	13	hexamerization.
	14	enhancement of heterodimerization H-H of bispecific antibodies (knobs-into-holes, charge steering, additional disulfide bridge).
	15	suppression of inter H-L and/or inter H-H disulfide bridges.
	16	site-specific drug attachment, e.g., additional cysteine.
	17	enhancement of heteropairing H-L of bispecific antibodies.
	18	control of H chain expression or of half-IG exchange of bispecific IgG by amino acid changes.

**Table 2:** IMGT-NC engineered variants characterized by published experimental data for effector properties and/or structure formats of the IG. These variants [101,102,103,104,105,106,107,108,109,110,111,112,113,114,115,116,117,118,119,120,121,122,123,124,125,126,127,128,129,130,131,132,133,134,135,136,137,138,139,140,141,142,143,144,145,146,147,148,149,150,151,152,153,154,155,156,157,158,159,160,161,162,163,164,165,166,167,168,169,170,171,172,173,174,175,176,177,178,179,180,181,182,183,184,185,186,187,188,189,190] have been used to define the standardized IMGT-NC rules for the description of the engineered variants of therapeutic IG or antibodies and related IgSF proteins (TR, FPIA and CPCA) [45,46], reported in Table S1.

IMGT-NC Engineered Variant ID	Species	IMGT-NC Engineered Variant Name	IMGT-NC Engineered Variant Definition	References
001	**Homsap**	**1-G1v1**	**CH2** **P1.4**	[101]
002	**Homsap**	**1-G1v1-2-3**	**CH2** **P1.4, V1.3, A1.2**	[101]
003	**Homsap**	**1-G1v2**	**CH2** **V1.3**	[101]
006	**Homsap**	**1-G1v3**	**CH2** **A1.2**	[101]
008	**Homsap**	**6-G1v4**	**CH2** **A114**	[102,103]
011	**Homsap**	**1,4-G1v5**	**CH2** **W109**	[104]
012	**Homsap**	**1,4-G1v5-2**	**CH2** **A1.3, A1.2, A109, S118 **LALA/KAES	[104,105,106,107]
013	**Homsap**	**2-G1v6**	**CH2** **A85.4, A118, A119 ** AAA	[102]
014	**Homsap**	**2,7-G1v7**	**CH2** **D3, E117 ** DE	[108]
016	**Homsap**	**2,5,7-G1v8**	**CH2** **D3, L115, E117 **DLE, 3M	[108,109]
017	**Homsap**	**2-G1v9**	**CH2 L7, P83, L85.2, I88, CH3 L83 ** LPLIL	[110]
019	**Homsap**	**2-G1v10**	**CH2** **Y1.3, Q1.2, W1.1, M3, D30, E34, A85.4**	[111]
020	**Homsap**	**2-G1v11**	**CH2** **E34, D109, M115, E119**	[111]
021	**Homsap**	**3-G1v12**	**CH2** **A1.1, D3, L115, E117 **GASDALIE	[112]
022	**Homsap**	**3-G1v13**	**CH2 A1.1, D3, E117 **GASDIE, ADE	[113]
023	**Homsap**	**6-G1v14**	**CH2** **A1.3, A1.2 **LALA	[105,106,114]
030	**Homsap**	**6-G1v14-49**	**CH2** **A1.3, A1.2, G114 **LALAPG	[114]
034	**Homsap**	**4-G1v15**	**CH2** **S118 ** ES	[104]
035	**Homsap**	**4-G1v15-1**	**CH2** **A109, S118 **KAES	[107]
036	**Homsap**	**4-G1v16**	**CH2** **W109, S118**	[104]
037	**Homsap**	**4-G1v17**	**CH2** **E29, F30, T107 ** EFT	[115]
038	**Homsap**	**4,10,13-G1v18**	**CH3** **R1, G109, Y120 ** IgG1-005-RGY	[116]
039	**Homsap**	**5-G1v19**	**CH2** **A34**	[103,105]
040	**Homsap**	**5-G1v20**	**CH2** **A105 **	[114]
041	**Homsap**	**9-G1v21**	**CH2** **Y15.1, T16, E18** YTE	[117,118,119,120]
042	**Homsap**	**9-G1v22**	**CH2** **Y15.1, T16, E18, CH3** **K113, F114, H116**	[117]
043	**Homsap**	**9-G1v22-1**	**CH2** **Y15.1, T16, E18, CH3** **K113, F114 **	[121]
044	**Homsap**	**6-G1v23**	**CH2** **E1.2**	[122,123]
045	**Homsap**	**9-G1v24**	**CH3** **L107, S114 **	[124]
047	**Homsap**	**7-G1v25**	**CH2** **E29, F113 **SELF	[125,126]
048	**Homsap**	**14-G1v26**	**CH3** **Y22 /14-G1v31**	[127]
052	**Homsap**	**8-G1v29**	**CH2** **A84.4**	[128,129]
054	**Homsap**	**8-G1v30**	**CH2** **G84.4**	[129,130]
055	**Homsap**	**8-G1v30-1**	**CH2** **H84.4 **	[129]
056	**Homsap**	**14-G1v31**	**CH3** **T86 /14-G1v26**	[127]
057	**Homsap**	**14-G1v31-1**	**CH3** **A86 /14-G1v32**	[131]
058	**Homsap**	**14-G1v32**	**CH3** **W22 /14-G1v33, /14-G1v31-1 **	[131]
060	**Homsap**	**14-G1v33**	**CH3 S22, A24, V86 /14-G1v32**	[131]
063	**Homsap**	**4-G1v35**	**CH2 E29 ** SE	[115,132]
064	**Homsap**	**8-G1v36**	**CH2** **Q84.4**	[129,130,133]
065	**Homsap**	**8-G1v36-1**	**CH2** **S84.4 **	[130]
068	**Homsap**	**6-G1v38**	**CH2** **S108, F113**	[134]
069	**Homsap**	**6-G1v39**	**CH2** **F1.3, E1.2, S116 ** FES, TM, LFLE-PS	[135,136]
070	**Homsap**	**6-G1v40**	**CH2** **A1.3,A1.2,S116**	[136]
071	**Homsap**	**6-G1v41**	**CH2** **F1.3, E1.2** FE	[122,137]
073	**Homsap**	**9-G1v42**	**CH2** **Q14, CH3** **L107**	[117]
080	**Homsap**	**3-G1v45**	**CH2** **A1.1, L115, E117 ** GAALIE	[138]
081	**Homsap**	**9-G1v46**	**CH3** **K113, F114 **	[139,140]
082	**Homsap**	**1-G1v47**	**CH2** **delG1.1**	[141]
084	**Homsap**	**6-G1v49**	**CH2** **G114 **	[114]
090	**Homsap**	**6-G1v50-79**	**CH2** **delE1.4, P1.3, V1.2, A1.1, G27, Q110, S115**	[142]
093	**Homsap**	**6-G1v53**	**CH2** **F1.3, Q1.2, Q105 ** FQQ	[143]
094	**Homsap**	**6-G1v53-1**	**CH2 A1.3, Q1.2, Q105 ** AQQ	[144]
095	**Homsap**	**11-G1v54**	**CH2** **C83, C85**	[145]
096	**Homsap**	**11,8-G1v54-29**	**CH2** **C83, A84.4, C85**	[130]
097	**Homsap**	**11,8-G1v54-30**	**CH2** **C83, G84.4, C85**	[130,145]
098	**Homsap**	**11,8-G1v54-36**	**CH2** **C83, Q84.4, C85**	[130]
100	**Homsap**	**16-G1v56**	**CH1** **F85.2(F = pAMF), CH3** **F85.2(F = pAMF)**	[146]
107	**Homsap**	**17-G1v58**	**CH1** **C5 hinge** **V5 /17-LC2v58**	[147]
108	**Homsap**	**17-G1v58-1**	**CH1** **C5, D20,** **hinge** **V5 /17-LC2v58-1**	[148]
109	**Homsap**	**17-G1v58-2**	**CH1** **C5, E20,** **hinge** **V5 /17-LC2v58-1**	[148]
110	**Homsap**	**6-G1v59**	**CH2** **S1.3, T1.2, R1.1** STR	[149]
113	**Homsap**	**6-G1v60**	**CH2** **S115, S116**	[136]
114	**Homsap**	**6-G1v60-1**	**CH2** **G110, S115, S116 **	[136]
118	**Homsap**	**6,7-G1v63-2**	**CH2** **D2**	[150,151]
119	**Homsap**	**6,7-G1v63-3**	**CH2** **D1.4, D1, D2, D30, G35, R115 **V12	[150,151]
125	**Homsap**	**14-G1v68**	**CH3 V6, L22, L79, W81 /14-G1v69 **	[152]
127	**Homsap**	**14-G1v69**	**CH3** **V6, Y7, A85.1, V86 /14-G1v68**	[152]
130	**Homsap**	**14-G1v72**	**CH3** **D7, E24 /14-G1v73**	[153]
132	**Homsap**	**14-G1v72-2**	**CH3 D79, D88 /14-G1v73-2, /14-G1v73-5**	[154]
134	**Homsap**	**14-G1v72-4**	**CH3** **H20, A85.1 /14-G1v73-4**	[155]
136	**Homsap**	**14-G1v73**	**CH3** **K7, K22 ** /**14-G1v72**	[153]
138	**Homsap**	**14-G1v73-2**	**CH3** **K12, K84.2 **/**14-G1v72-2**	[154]
140	**Homsap**	**14-G1v73-4**	**CH3** **T5, F81 /14-G1v72-4**	[155]
143	**Homsap**	**14-G1v74**	**CH3** **C10 /14-G1v75**	[156,175]
144	**Homsap**	**14-G1v75**	**CH3** **C5 /14-G1v74**	[156,175]
146	**Homsap**	**9-G1v77**	**CH3** **A115 **	[157]
150	**Homsap**	**9-G1v78-1**	**CH3** **L107,** **A114,** **T116**	[158,159]
151	**Homsap**	**9-G1v78-2**	**CH3** **A114**	[159]
157	**Homsap**	**18-G1v82-1**	**CH3 L85.1 /18-G1v82-2**	[160]
158	**Homsap**	**18-G1v82-2**	**CH3 R88 /18-G1v82-1 **	[160]
159	**Homsap**	**18-G1v82-3**	**CH3** **K85.1 /18-G1v82-4**	[161]
160	**Homsap**	**18-G1v82-4**	**CH3** **A88 /18-G1v82-3**	[161]
161	**Homsap**	**18-G1v82-5**	**CH3** **D24, S26 /18-G1v82-6**	[162]
162	**Homsap**	**18-G1v82-6**	**CH3** **Q13, K20 **/**18-G1v82-5**	[162]
164	**Homsap**	**18,10-G1v82-8**	*chimerisotype G1 [8aaTRAC,7aaG3,1aa°] /* **18,10-G1v82-9**	[163,164,165]
165	**Homsap**	**18,10-G1v82-9**	*chimerisotype G1 [11aaTRBC,3aa°] * */***18,10-G1v82-8**	[163,164]
166	**Homsap**	**10-G1v83**	**CH3** **R115, F116 ** RF	[147,165]
171	**Homsap**	**7-G1v85**	**CH2** **W1.2,** **N1.1, D30, K115**	[150,159]
172	**Homsap**	**7-G1v86**	**CH2** **Y1.3, D2,** **I26,** **K115 **	[150]
173	**Homsap**	**10-G1v87**	**CH2** **V14, P90 **	[158,159]
174	**Homsap**	**10-G1v88**	**CH2** **R94,** **CH3** **R1.2 **	[158]
175	**Homsap**	**10-G1v88-1**	**CH2** **R94, CH3** **K92 **	[158,159]
176	**Homsap**	**10-G1v89**	**CH3** **R118, E120 **	[161]
177	**Homsap**	**14-G1v90**	**CH3** **E16, W88 /14-G1v91**	[166,175]
178	**Homsap**	**14-G1v91**	**CH3** **R3, V84.2, T85.1 /14-G1v90**	[166,175]
186	**Homsap**	**6-G1v97**	**CH2** **A1.3,** **A1.2, A1, S2, A30, S115, S116 ** G1sigma	[167]
187	**Homsap**	**10,13-G1v98**	**CH3** **R1**	[116]
188	**Homsap**	**10,13-G1v98-1**	**CH3** **K1**	[168]
191	**Homsap**	**14-G1v99-3**	**hinge** **E6, E13, CH3** **E24**	[169]
192	**Homsap**	**14-G1v99-4**	**hinge** **R6, R13, CH3** **R88**	[169]
198	**Homsap**	**3-G1v104**	**CH2** **A85.3 **	[170]
199	**Homsap**	**6-G1v104-1**	**CH2** **N85.4, A85.3, S85.2 ** NNAS	[171]
702	**Homsap**	**4-G1v107**	*chimerisotype G1 [10aaG3]*	[172]
708	**Homsap**	**18-G1v113**	*chimerisotype* *G1_AG SEED CH3 [26aaA1]*	[173]
709	**Homsap**	**18-G1v114**	*chimerisotype* *G1_GA SEED CH3 [34aaA1]*	[173]
710	**Homsap**	**3,4-G1A1v1**	*chimerisotype* *G1-CH1hCH2 [10aaA1]-A1 [CH3]*	[174]
201	**Homsap**	**2-G2v1**	**CH2 P1.4, L1.3, L1.2, G1.1 ** G1-like	[101]
202	**Homsap**	**6-G2v2**	**CH2** **Q30, L92, S115, S116 **IgG2m4	[176]
203	**Homsap**	**6-G2v3**	**CH2** **A1.2, A1, S2, A30, L92, S115, S116 **G2sigma	[137]
205	**Homsap**	**9-G2v4**	**CH2** **Q14**	[177]
206	**Homsap**	**9-G2v5**	**CH3** **L107**	[177]
207	**Homsap**	**9-G2v6**	**CH2** **Q14** **CH3** **L107**	[177]
208	**Homsap**	**10,6-G2v7**	**CH2** **Y85.2, L92, A339 **	[178]
209	**Homsap**	**9-G2v8-1**	**CH2** **A93 **	[120]
218	**Homsap**	**6-G2v59**	**CH2** **S1.3,T1.2, R1.1 ** STR	[149]
228	**Homsap**	**6-G2v94**	*chimerisotype G2 [11aaG4]*	[179]
301	**Homsap**	**9,10-G3v1**	**CH3** **H115**	[180]
302	**Homsap**	**5-G3v20**	**CH2** **A105**	[181]
303	**Homsap**	**6-G3v59**	**CH2** **S1.3,T1.2, R1.1** STR	[149]
401	**Homsap**	**2-G4v1**	**CH2** **L1.3 **	[101]
402	**Homsap**	**4-G4v2**	**CH2** **P116 **	[182]
403	**Homsap**	**6-G4v3**	**CH2** **E1.2** LE	[122]
406	**Homsap**	**6-G4v3-49**	**CH2** **E1.2,** **G114** LEPG	[114]
407	**Homsap**	**6-G4v4**	**CH2** **A1.3, A1.2** FALA	[106]
408	**Homsap**	**12-G4v5**	**hinge** **P10 **	[183]
409	**Homsap**	**12-G4v6**	**CH3** **K88 **	[184]
421	**Homsap**	**9-G4v21**	**CH2** **Y15.1, T16, E18 ** YTE	[117]
422	**Homsap**	**9-G4v22**	**CH2** **T16, P91 CH3** **A114**	[185]
427	**Homsap**	**6-G4v34**	**CH2** **A1.3,A1.2, A1, S2** G4sigma1	[167,186]
428	**Homsap**	**6-G4v35**	**CH2 A1.3, A1.2, delG1.1, A1, S2 ** G4sigma2	[167]
429	**Homsap**	**8-G4v36**	**CH2** **Q84.4**	[129]
432	**Homsap**	**6-G4v49**	**CH2** **G114 **	[114]
437	**Homsap**	**6-G4v59**	**CH2** **S1.3,T1.2, R1.1 ** STR	[149]
536	**Homsap**	**17-KCv100-1**	**C-KAPPA** **N87**	[187]
577	**Homsap**	**17-LC2v58**	**C-LAMBDA2** **C10, V126 /17-G1v58 **	[147]
578	**Homsap**	**17-LC2v58-1**	**C-LAMBDA2** **R5, C10, V126 /17-G1v58-1, /17-G1v58-1 **	[148]
603	**Canlupfam**	**8-G2v29**	**CH2** **A84.4**	[129]
646	**Musmusus**	**6-G2Av1**	**CH2** **A1.3, A1.2** LALA	[188]
647	**Musmusu**	**2-G2Bv1**	**CH2** **L1.2**	[189]
648	**Musmusu**	**5-G2Bv2**	**CH2** **A101**	[190]
649	**Musmusu**	**5-G2Bv3**	**CH2** **A103**	[190]
650	**Musmusu**	**5-G2Bv4**	**CH2** **A105**	[190]

## Data Availability

No new data were created or analyzed in this study. Data sharing is not applicable to this article.

## Supplementary Materials

The following supporting information can be downloaded at: https://www.mdpi.com/article/10.3390/biom16091264/s1, Table S1. IMGT^®^ nomenclature (IMGT-NC) of 335 engineered variants involved in effector properties (ADCC, ADCP, CDC), half-life physicochemical properties and structure of therapeutic monoclonal antibodies, fusion proteins for immune applications (FPIA) and composite proteins for clinical applications (CPCA). Table S2. The 228 IMGT-NC engineered variants assigned to INN.

## Abbreviations

The following abbreviations are used in this manuscript:

2D	Two-dimensional
3D	Three-dimensional
ADCC	Antibody-dependent cellular cytotoxicity
ADCP	Antibody-dependent cellular phagocytosis
AI	Artificial intelligence
AIR	Adaptive immune response
AR	Antigen receptor (IG and/or TR)
BcR	B cell receptor (mIG with coreceptors CD79A and CD79B)
C	Constant (gene type, domain type)
CDC	Complement-dependent cytotoxicity
CDR	Complementarity determining region
CNRS	Centre National de la Recherche Scientifique
CNV	Copy number variation
CPCA	Composite protein for clinical applications
CSR	Class switch recombination
D	Diversity (gene type)
DDBJ	DNA Database of Japan
EMBL	European Molecular Biology Laboratory
ENA	European Nucleotide Archive
Fc	Fragment crystallizable
FcγR	Fc gamma receptor
FPIA	Fusion protein for immune applications
FR	Framework region
FWD	Forward (IG and TR locus orientation on chromosome)
G	groove (domain type)
GEDI	GenBank_EMBL(ENA)_DDBJ_IMGT/LIGM-DB
H	Heavy (chain)
HGM	Human Gene Mapping
HGNC	HUGO Gene Nomenclature Committee
IG	Immunoglobulin or antibody
IGH	IG heavy (locus)
IGK	IG kappa (locus)
IGL	IG lambda (locus)
IgSF	Immunoglobulin superfamily
IMGT	ImMunoGeneTics
IMGT-NC	IMGT nomenclature
INN	International Nonproprietary Name
J	Joining (gene type)
LIGM	Laboratoire d’ImmunoGénétique Moléculaire
mAb	monoclonal antibody
mIG	Membrane IG
MH	Major histocompatibility
MH1	MH class I
MH2	MH class II
MhSF	MH superfamily
NCBI	National Center for Biotechnology Information
PDB	Protein Data Bank
RCSB	Research Collaboratory for Structural Bioinformatics
REV	Reverse (IG and TR locus orientation on chromosome)
RPI	Related protein of the immune system
SHM	Somatic hypermutation
sIG	Secreted IG
TcR	T cell receptor (TR with coreceptors CD3)
TR	T cell receptor
TRA	TR alpha (locus)
TRB	TR beta (locus)
TRD	TR delta (locus)
TRG	TR gamma (locus)
UM	Université de Montpellier
V	Variable (gene type, domain type)
VH	Variable domain of heavy (IG chain)
VL	Variable domain of light (IG chain)
WHO	World Health Organization
