# Supplementary material for: IMGT-NC Engineered Variants of INN Therapeutic IG or Antibodies and Related IgSF Proteins (TR, FPIA and CPCA): Bridging Sequences, Structures and Functions for AI
Source: Biomolecules. 2026 Sep 1;16(9):1264. doi: 10.3390/biom16091264 (PMC13604298; doi:10.3390/biom16091264)
Supplement: Supplementary file 1 [file biomolecules-16-01264-s001.zip › Table S1 _biomolecules-4493254_Lefranc_260826.pdf]

Table S1. IMGT® nomenclature (IMGT-NC) of 335 engineered variants involved in effector properties (ADCC, ADCP, CDC), half-life, physicochemical properties and structure of therapeutic monoclonal antibodies, fusion proteins for immune applications (FPIA) and composite proteins for clinical applications (CPCA).

In column 10 (the most right column), the bold number in red indicates the number of WHO INN entries, up to lists R95 and PL134, included. The first (one or two) INN name(s), INN number(s) and, between parentheses, proposed (PL) and recommended (RL) list numbers, are displayed in green italics. '0' in black in the column means no INN. (Author and Editor Marie-Paule Lefranc marie-paule.lefranc@umontpellier.fr, 14/07/2026). (With permission from M-P. Lefranc and G. Lefranc, LIGM, Founders and Authors of IMGT®, the international ImMunoGeneTics information system®, https://www.imgt.org

IMGT-NC variant ID	IMGT-NC engineered variant name		IMGT-NC engineered variant definition	IMGT amino acid (aa) change(s) per domain according to the IMGT unique numbering (Eu-IMGT positions in parentheses)	Amino acid (aa) changes with Eu-IMGT positions	IMGT topological motifs identifiable in gene and domain with positions according to the IMGT unique numbering (Eu-IMGT positions between parentheses)	1. Property and function	2. Property and function	3. Property and function /3D structure
	Species	Variant name							
**001**	**Homsap**	**1-G1v1**	**CH2**<br>**P1.4**	CH2<br>E1.4>P (233)	E233P	IGHG1 CH2<br>1.6-3 (231-239)<br>APELLGGPS ><br>APPLLGGPS	**ADCC reduction**.<br>Prevents FcγRI binding [101].		0
**002**	**Homsap**	**1-G1v1-2-3**	**CH2**<br>**P1.4,**<br>**V1.3,**<br>**A1.2**	CH2<br>E1.4>P (233)<br>L1.3>V (234)<br>L1.2>A (235)	E233P,<br>L234V,<br>L235A	IGHG1 CH2<br>1.6-3 (231-239)<br>APELLGGPS ><br>APPVAGGPS	**ADCC reduction**.<br>Decreases FcγRI binding [101].		Combines 1-G1v1, 1-G1v2 and 1-G1v3.<br>**1** *levilimab 10905 (120,82).*
**003**	**Homsap**	**1-G1v2**	**CH2**<br>**V1.3**	CH2<br>L1.3>V (234)	L234V	IGHG1 CH2<br>1.6-3 (231-239)<br>APELLGGPS ><br>APEVLGGPS	**ADCC reduction**.<br>Decreases FcγRI binding [101].		0
**006**	**Homsap**	**1-G1v3**	**CH2**<br>**A1.2**	CH2<br>L1.2>A (235)	L235A	IGHG1 CH2<br>1.6-3 (231-239)<br>APELLGGPS ><br>APELAGGPS	**ADCC reduction**.<br>Prevents FcγRI binding [101].		0
**007**	**Homsap**	**6-G1v3-1**	**CH2**<br>**A1.2**<br>**A1**	CH2<br>L1.2>A (235)<br>G1>A (237)	L235A<br>G237A	IGHG1 CH2<br>1.6-3 (231-239)<br>APELLGGPS ><br>APELAGAPS	**ADCC reduction**.<br>Prevents FcγRI binding.	**CDC reduction**.<br>Reduces C1q binding.	**5**<br>*vedolizumab 9093 (100,62)…*

**008**	**Homsap**	**6-G1v4**	**CH2**<br>**A114**	CH2<br>**P**114>**A** (329)	P329A	IGHG1 CH2<br>FG 105-117 (322-332)<br>KVSNKA..LPAPI ><br>KVSNKA..LAAPI	**ADCC reduction**.<br>Reduces FcγR binding [102].	**CDC reduction**.<br>Reduces C1q binding [103].	**1**<br>*lifonebart 13851 (134,).*
**009**	**Homsap**	**6-G1v4-2**	**CH2**<br>**R114**	CH2<br>**P**114>**R** (329)	P329R	IGHG1 CH2<br>FG 105-117 (322-332)<br>KVSNKA..LPAPI ><br>KVSNKA..LRAPI	**ADCC reduction**.<br>Reduces FcγR binding.	**CDC reduction**.<br>Reduces C1q binding.	**1**<br>*tixestobart 13358 (132,94).*
**010**	**Homsap**	**6-G1v4-66**	**CH2**<br>**A27,**<br>**A114**	CH2<br>**D**27>**A** (265)<br>**P**114>**A** (329)	D265A,<br>P329A	IGHG1 CH2<br>23-31 (261-269)<br>CVVVDVSHE ><br>CVVVAVSHE<br>FG 105-117 (322-332)<br>KVSNKA..LPAPI ><br>KVSNKA..LAAPI	**ADCC reduction**.<br>Reduces FcγR binding.	**CDC reduction**.<br>Reduces C1q binding.	Combines 6-G1v4 (CH2 A114) and 1-G1v66 (CH2 A27).<br>**1** *abelacimab 10881 (119,81).*
**011**	**Homsap**	**1, 4-G1v5**	**CH2**<br>**W109**	**CH2**<br>**K**109>**W** (326)	K326W	IGHG1 CH2<br>FG 105-117 (322-332)<br>KVSNKA..LPAPI ><br>KVSNWA..LPAPI	**ADCC reduction**<br>Reduces FcγR binding [104].	**CDC enhancement**.<br>Increases C1q binding [104].	0
**012**	**Homsap**	**1, 4-G1v5-2**	**CH2**<br>**A1.3,**<br>**A1.2,**<br>**A109,**<br>**S118**	**L**1.3>**A** (234)<br>**L**1.2>**A** (235),<br>**K**109>**A** (326),<br>**E**118>**S** (333)<br>LALA/KAES	L234A,<br>L235A,<br>K326A,<br>E333S	IGHG1 CH2<br>1.6-3 (231-239)<br>APELLGGPS ><br>APEAAGGPS<br>FG 105-117 (322-332)<br>KVSNKA..LPAPI ><br>KVSNAA..LPAPI<br>G 118-125 (333-340)<br>EKTISKAK ><br>SKTISKAK	**ADCC reduction**<br>Reduces FcγR binding.<br>LALA [105], [106].	**CDC enhancement**.<br>Increases C1q binding.<br>KAES [107].<br>Includes 4-G1v15 ES [104].	Combines 6-G1v14 (CH2 A1.3, A1.2) 'LALA' and 6-G1v15-1 (CH2 A109, S118) 'KAES' (which includes 4-G1v15 'ES'.<br>0
**013**	**Homsap**	**2-G1v6**	**CH2**<br>**A85.4,**<br>**A118,**<br>**A119**	CH2<br>**S**85.4>**A** (298),<br>**E**118>**A** (333),<br>**K**119>**A** (334)<br>AAA	S298A,<br>E333A,<br>K334A	IGHG1 CH2<br>83-88 (292-305)<br>REEQYN..STYRVVSV ><br>REEQYN..ATYRVVSV<br>G 118-125 (322-340)<br>EKTISKAK ><br>AATISKAK	**ADCC enhancement**.<br>Increases FcγRIIIa binding (50-100 fold more potent cytotoxicity) [102].		**7**<br>*gotistobart 12383 (128,90)...*

**014**	**Homsap**	**2,7-G1v7**	**CH2**<br>**D3,**<br>**E117**	CH2<br>S3>D (239),<br>I117>E (332)<br>DE	S239D,<br>I332E	IGHG1 CH2<br>1.6-3 (231-239)<br>APELLGGPS ><br>APELLGGPD<br>FG 105-117 (322-332)<br>KVSNKA..LPAPI ><br>KVSNKA..LPAPE	**ADCC enhancement.** Increases FcγRIIIA binding (2-log enhancement compared to wildtype Fc) [108].	**FcγRIIB.** Strong binding to inhibitory FcγRIIB [108].	**6**<br>*talacotuzumab 10508 (117,79)...*
**015**	**Homsap**	**10-G1v7-1**<br>*chimerisotype G1[8aaG2]*	**CH2**<br>**Q38,**<br>**F84.3,**<br>**F85.2,**<br>**V92,**<br>**T124,**<br>**CH3**<br>**E12,**<br>**M14,**<br>**M84**	CH2<br>K38>Q (274),<br>Y84.3>F (296),<br>Y85.2>F (300),<br>L92>V (309),<br>A124>T (339),<br>CH3<br>D12>E (356),<br>L14>M (358),<br>V84>M (332)	K274Q,<br>Y296F,<br>Y300F,<br>L309V,<br>A339T,<br><br>D356E,<br>L358M,<br>V397M	IGHG1 CH2<br>34-41 (270-277)<br>DPEVKFNW ><br>DPEVQFNW<br>83-93 (292-310)<br>REEQYN..STYRVVSVLTVLH ><br>REEQFN..STFRVVSVLTVVH<br>G 118-125 (333-340)<br>EKTISKAK ><br>EKTISKTK<br>CH3<br>7-15 (351-359)<br>LPPSRDELT ><br>LPPSREEMT<br>77-84 (390-397)<br>NYKTTPPV ><br>NYKTTPPM	IGHG1*01 backbone with eight G2 aa, five in CH2 (Q38, F84.3, F85.2, V92, T124) and three in CH3 (E12, M14 and M84), making the CH2 starting from Q38 and the CH3 identical to those of IGHG2*01, at the exception of CH2 A110 conserved from IGHG1*01.	*CH3 E12 and M14 are isoallotypic aa present in IGHG1*03 and in G2, G3 and G4.*	**2**<br>*talacotuzumab 10508 (117,79), tafasitamab 10835 (119,81).*
**016**	**Homsap**	**2,5,7-G1v8**	**CH2**<br>**D3,**<br>**L115,**<br>**E117**	CH2<br>S3>D (239),<br>A115>L (330),<br>I117>E (332)<br>DLE, 3M	S239D,<br>A330L,<br>I332E	IGHG1 CH2<br>1.6-3 (231-239)<br>APELLGGPS ><br>APELLGGPD<br>FG 105-117 (322-332)<br>KVSNKA..LPAPI ><br>KVSNKA..LPLPE	**ADCC enhancement.** Increases FcRIIIA affinity (2-log enhancement of DLE-trastuzumab compared to wildtype trastuzumab [108].	**FcγRIIB.** Low binding to inhibitory FcγRIIB [108].<br>**CDC reduction.** Reduces C1q binding [108].	**3D**<br>2ql1<br>[109].<br>**1**<br>*botensilimab 11659 (124,86).*
**017**	**Homsap**	**2-G1v9**	**CH2**<br>**L7,**<br>**P83,**<br>**L85.2,**	CH2<br>F7>L (243),<br>R83>P (292),<br>Y85.2>L (300),	F243L,<br>R292P,<br>Y300L,	IGHG1 CH2<br>4-12 (240-248)<br>VFLFPPKPK ><br>VFLLPPKPK	**ADCC enhancement.** 100% increase [110].		0

			**I88**<br><br><br>**CH3**<br>**L83**	V88>I (305)<br><br><br>CH3<br>P83>L (396)<br>LPLIL	V305I<br><br><br><br>P396L	83-88 (292-305)<br>REEQYN..STYRVVSV ><br>PEEQYN..STLRVVSI<br>CH3<br>83-84.4 (396-401)<br>PVLDSD ><br>LVLDSD			
**018**	**Homsap**	**2-G1v9-1**	**CH2**<br>**V1.2,**<br>**L7,**<br>**P83,**<br>**L85.2,**<br>**CH3**<br>**L83**	CH2<br>L1.2>V (235)<br>F7>L (243)<br>R83>P (292)<br>Y85.2>L (300)<br>CH3<br>P83>L (396)<br>VLPLL	 L235V,<br>F243L,<br>R292P,<br>Y300L,<br>P396L	IGHG1 CH2<br>1.6-10 (231-246)<br>APELLGGPSVFLFPPK ><br>APELVGGPSVFLLPPK<br>83-88 (292-305)<br>REEQYN..STYRVVSV ><br>PEEQYN..STLRVVSV<br>IGHG1 CH3<br>83-84.4 (396-401)<br>PVLDSD ><br>LVLDSD	**ADCC enhancement**.<br>Increases FcγIIIA binding.	**FcγRIIB.**<br>Decreases binding to inhibitory FcγRIIB.	2<br>*margetuximab 9799 (109,71), enoblituzumab 10165 (114/116,76/77).*
**019**	**Homsap**	**2-G1v10**	**CH2**<br>**Y1.3,**<br>**Q1.2,**<br>**W1.1,**<br>**M3,**<br>**D30,**<br>**E34,**<br>**A85.4**	CH2<br>L1.3>Y (234),<br>L1.2>Q (235),<br>G1.1>W (236),<br>S3>M (239),<br>H30>D (268),<br>D34>E (270),<br>S85.4>A (298)	 L234Y,<br>L235Q,<br>G236W,<br>S239M,<br>H268D,<br>D270E,<br>S298A	IGHG1 CH2<br>1.6-3 (231-239)<br>APELLGGPS ><br>APEYQWGPM<br>23-31 (261-269)<br>CVVVDVSHE ><br>CVVVDVSDE<br>34-41 (270-277)<br>DPEVKFNW ><br>EPEVKFNW<br>83-88 (292-305)<br>REEQYN..STYRVVSV ><br>REEQYN..ATYRVVSV	**ADCC enhancement**.<br>Increases FcγIIIA binding >2000-fold (F158), >1000-fold (V158) in the association of 2-G1v10 and 2-G1v11 [111].		0

**020**	**Homsap**	**2-G1v11**	**CH2**<br>**E34,**<br>**D109,**<br>**M115,**<br>**E119**	CH2<br>D34>E (270),<br>K109>D (326),<br>A115>M (330)<br>K119>E (334)	D270E,<br>K326D,<br>A330M,<br>K334E	IGHG1 CH2<br>34-41 (270-277)<br>DPEVKFNW ><br>EPEVKFNW<br>FG 105-117 (322-332)<br>KVSNKA..LPAPI<br>KVSNDA..LPMPI<br>G 118-125 (333-340)<br>EKTISKAK ><br>EETISKAK	**ADCC enhancement**.<br>Increases FcγIIIA binding >2000-fold (F158), >1000-fold (V158) in the association of G1v10 and G1v11 [111].		0
**021**	**Homsap**	**3-G1v12**	**CH2**<br>**A1.1,**<br>**D3,**<br>**L115,**<br>**E117**	CH2<br>G1.1>A (236),<br>S3>D (239),<br>A115>L (330),<br>I117>E (332)<br>GASDALIE	G236A,<br>S239D,<br>A330L,<br>I332E	IGHG1 CH2<br>1.6-3 (231-239)<br>APELLGGPS ><br>APELLAGPD<br>FG 105-117 (322-332)<br>KVSNKA..LPAPI ><br>KVSNKA..LPLPE	**ADCC enhancement.**<br>Increases binding affinity to FcγRIIA and FcγRIIIA [112].	**ADCP enhancement**.<br>NK cell activation.<br>Increases FcγRIIA binding [112].	**3D**<br>5d4q,<br>5d6d<br>[112].<br>**1**<br>*elipovimab 10988 (120,82).*
**022**	**Homsap**	**3-G1v13**	**CH2**<br>**A1.1,**<br>**D3,**<br>**E117**	CH2<br>G1.1>A (236),<br>S3>D (239),<br>I117>E (332)<br>GASDIE, ADE	G236A,<br>S239D,<br>I332E	IGHG1 CH2<br>1.6-3 (231-239)<br>APELLGGPS ><br>APELLAGPD<br>FG 105-117 (322-332)<br>KVSNKA..LPAPI ><br>KVSNKA..LPAPE	**ADCC enhancement**.<br>Increases FcγIIIA binding [113].	**ADCP enhancement**.<br>NK cell activation.<br>Increases FcγRIIA binding (70>fold).<br>Increases FcγRIIA/FcγRIIB binding ratio (15-fold) [113].	**2**<br>*ipsoprubart 13680 (133,95), emzotamig 13697 (133,95).*
**023**	**Homsap**	**6-G1v14**	**CH2**<br>**A1.3,**<br>**A1.2**	CH2<br>L1.3>A (234),<br>L1.2>A (235)<br>LALA	L234A,<br>L235A	IGHG1 CH2<br>1.6-3 (231-239)<br>APELLGGPS ><br>APEAAGGPS	**ADCC reduction**.<br>Reduces FcγR binding [105], [106], [114].	**CDC reduction**.<br>Reduces C1q binding [105], [106], [114].	**65**<br>*teplizumab 8869 (87/97,59)…*
**024**	**Homsap**	**6-G1v14-1**	**CH2**<br>**A1.3,**<br>**A1.2,**<br>**A1**	CH2<br>L1.3>A (234),<br>L1.2>A (235),<br>G1>A (237)	L234A,<br>L235A,<br>G237A	IGHG1 CH2<br>1.6-3 (231-239)<br>APELLGGPS ><br>APEAAGAPS	**ADCC reduction**.<br>Reduces FcγR binding.	**CDC reduction**.<br>Reduces C1q binding.	Combines Homsap 6-G1v14 (CH2 A1.3, A1.2) and G1 CH2 A1.<br>**23** *domagrozumab 10286 (114,76)...*

**025**	**Homsap**	**6,5-G1v14-1-20**	**CH2**<br>**A1.3,**<br>**A1.2,**<br>**A1,**<br>**A105**	CH2<br>L1.3>A (234),<br>L1.2>A (235),<br>G1>A (237)<br>K105>A (322)	L234A,<br>L235A,<br>G237A,<br>K322A	IGHG1 CH2<br>1.6-3 (231-239)<br>APELLGGPS ><br>APEAAGAPS<br>IGHG1 CH2<br>FG 105-117 (322-332)<br>KVSNKA..LPAPI ><br>AVSNKA..LPAPI	**ADCC reduction**.<br>Reduces FcγR binding.<br>(6-G1v14-1)	**CDC reduction**.<br>Reduces C1q<br>binding.<br>(6-G1v14-1,<br>5-G1v20).	Combines 6-G1v14-1 (CH2 A1.3, A1.2, A1) and 5-G1v20 (CH2 A105).<br>**5**<br>*emfizatamab 12023 (126,88)...*
**026**	**Homsap**	**6-G1v14-1-29**	**CH2**<br>**A1.3,**<br>**A1.2,**<br>**A1,**<br>**A84.4**	CH2<br>L1.3>A (234),<br>L1.2>A (235),<br>G1>A (237),<br>N84.4>A (297)	L234A,<br>L235A,<br>G237A,<br>N297A	IGHG1 CH2<br>1.6-3 (231-239)<br>APELLGGPS ><br>APEAAGAPS<br>83-88 (292-305)<br>REEQYNSTYRVVSV ><br>REEQYASTYRVVSV	**ADCC reduction**.<br>Reduces FcγR binding.	**CDC reduction**.<br>Reduces C1q<br>binding.	Combines Homsap 6-G1v14-1 (CH2 A1.3, A1.2, A1) and 8-G1v29 (CH2 A84.4).<br>0
**027**	**Homsap**	**6-G1v14-4**	**CH2**<br>**A1.3,**<br>**A1.2,**<br>**A114**	CH2<br>L1.3>A (234),<br>L1.2>A (235,<br>P114>A (329)	L234A,<br>L235A,<br>P329A	IGHG1 CH2<br>1.6-3 (231-239)<br>APELLGGPS ><br>APEAAGGPS<br>FG 105-117 (322-332)<br>KVSNKA..LPAPI ><br>KVSNKA..LAAPI	**ADCC reduction**.<br>Reduces FcγR binding.	**CDC reduction**.<br>Reduces C1q.<br>binding.	Combines Homsap 6-G1v14 (CH2 A1.3, 1.2) and 6-G1v4 (CH2 A114).<br>**2** *tulisokibart 12368 (127,89), opamtistomig 13330 (132,94).*
**028**	**Homsap**	**6,5-G1v14-20**	**CH2**<br>**A1.3,**<br>**A1.2,**<br>**A105**	CH2<br>L1.3>A (234),<br>L1.2>A (235),<br>K105>A (322)	L234A,<br>L235A,<br>K322A	IGHG1 CH2<br>1.6-3 (231-239)<br>APELLGGPS ><br>APEAAGGPS<br>FG 105-117 (322-332)<br>KVSNKA..LPAPI ><br>AVSNKA..LPAPI	**ADCC reduction**.<br>Reduces FcγR binding.	**CDC reduction**.<br>Reduces C1q<br>binding.	Combines Homsap 6-G1v14 (CH2 A1.3, 1.2) and 5-G1v20 (CH2 A105).<br>0
**029**	**Homsap**	**6-G1v14-48**	**CH2**<br>**A1.3,**<br>**A1.2,**<br>**R113**	CH2<br>L1.3>A (234),<br>L1.2>A (235),<br>L113>R (328)	L234A,<br>L235A,<br>L328R	IGHG1 CH2<br>1.6-3 (231-239)<br>APELLGGPS ><br>APEAAGGPS<br>FG 105-117 (322-332)<br>KVSNKA..LPAPI >	**ADCC reduction**.<br>Reduces FcγR binding.	**CDC reduction**.<br>Reduces C1q<br>binding.	Combines Homsap 6-G1v14 (CH2 A1.3, 1.2) and 6-G1v48 (CH2 R113).<br>**4** *delpasibart 12399 (127,89)...*

						KVSNKA..RPAPI			
030	**Homsap**	**6-G1v14-49**	**CH2**<br>**A1.3,**<br>**A1.2,**<br>**G114**	CH2<br>**L**1.3>**A** (234),<br>**L**1.2>**A** (235),<br>**P**114>**G** (329)<br>LALAPG	L234A,<br>L235A,<br>P329G	IGHG1 CH2<br>1.6-3 (231-239)<br>APELLGGPS ><br>APEAAGGPS<br>FG 105-117 (322-332)<br>KVSNKA..LPAPI ><br>KVSNKA..LGAPI	**ADCC reduction**.<br>Reduces FcγR binding [114].	**CDC reduction**.<br>Reduces C1q binding [114].	Combines Homsap 6-G1v14 (CH2 A1.3, A1.2) and 6-G1v49 (CH2 G114).<br>**16** *cergutuzumab amunaleukin 10080 (113,75)...*
031	**Homsap**	**6-G1v14-66**	**CH2**<br>**A1.3,**<br>**A1.2,**<br>**A27**	CH2<br>**L**1.3>**A** (234),<br>**L**1.2>**A** (235),<br>**D**27>**A** (265)	L234A,<br>L235A,<br>D265A	IGHG1 CH2<br>1.6-3 (231-239)<br>APELLGGPS ><br>APEAAGGPS<br>23-31 (261-269)<br>CVVVDVSHE ><br>CVVVAVSHE	**ADCC reduction**.<br>Reduces FcγR binding.	**CDC reduction**.<br>Reduces C1q binding.	Combines Homsap 6-G1v14 (CH2 A1.3, A1.2) and 1-G1v66 (CH2 A27).<br>0
032	**Homsap**	**6-G1v14-67**	**CH2**<br>**A1.3,**<br>**A1.2,**<br>**S27**	CH2<br>**L**1.3>**A** (234),<br>**L**1.2>**A** (235),<br>**D**27>**S** (265)	L234A,<br>L235A,<br>D265S	IGHG1 CH2<br>1.6-3 (231-239)<br>APELLGGPS ><br>APEAAGGPS<br>23-31 (261-269)<br>CVVVDVSHE ><br>CVVVSVSHE	**ADCC reduction**.<br>Reduces FcγR binding.	**CDC reduction**.<br>Reduces C1q binding.	Combines Homsap 6-G1v14 (CH2 A1.3, A1.2) and 1-G1v67 (CH2 S27).<br>**7** *reozalimab 11904 (126,88)...*
033	**Homsap**	**6-G1v14-109**	**CH2**<br>**A1.3,**<br>**A1.2,**<br>**S114**	CH2<br>**L**1.3>**A** (234),<br>**L**1.2>**A** (235),<br>**P**114>**S** (329)	L234A,<br>L235A,<br>P329S	IGHG1 CH2<br>1.6-3 (231-239)<br>APELLGGPS ><br>APEAAGGPS<br>FG 105-117 (322-332)<br>KVSNKA..LPAPI ><br>KVSNKA..LSAPI	**ADCC reduction**.<br>Reduces FcγR binding.	**CDC reduction**.<br>Reduces C1q binding.	Combines 6-G-G1v14 (CH2 A1.3, A1.2) and 1-G1v109 (G1 CH2 S114).<br>0
034	**Homsap**	**4-G1v15**	**CH2**<br>**S118**	CH2<br>**E**118>**S** (333)	E333S	IGHG1 CH2<br>G 118-125 (333-340)<br>EKTISKAK ><br>SKTISKAK	**CDC enhancement**.<br>Increases C1q binding [104].		0

**035**	**Homsap**	**4-G1v15-1**	**CH2**<br>**A109,**<br>**S118**	CH2<br>**K**109>**A** (326),<br>**E**118>**S** (333)<br>KAES	K326A,<br>E333S	IGHG1 CH2<br>FG 105-117 (322-332)<br>KVSNKA..LPAPI ><br>KVSNAA..LPAPI<br>G 118-125 (333-340)<br>EKTISKAK ><br>SKTISKAK	**CDC enhancement**.<br>Increases C1q binding [107].		0
**036**	**Homsap**	**4-G1v16**	**CH2**<br>**W109,**<br>**S118**	CH2<br>**K**109>**W** (326),<br>**E**118>**S** (333)	K326W,<br>E333S	IGHG1 CH2<br>FG 105-117 (322-332)<br>KVSNKA..LPAPI ><br>KVSNWA..LPAPI<br>G 118-125 (333-340)<br>EKTISKAK ><br>SKTISKAK	**CDC enhancement**.<br>Increases C1q binding [104].		0
**037**	**Homap**	**4-G1v17**	**CH2**<br>**E29,**<br>**F30,**<br>**T107**	CH2<br>**S**29>**E** (267),<br>**H**30>**F** (268),<br>**S1**07>**T** (324)<br>EFT	S267E,<br>H268F,<br>S324T	IGHG1 CH2<br>23-31 (261-269)<br>CVVVDVSHE ><br>CVVVDVEFE<br>FG 105-117 (322-332)<br>KVSNKA..LPAPI ><br>KVTNKA..LPAPI	**CDC enhancement.**<br>Increases C1q binding [115].		0
**038**	**Homsap**	**4,10,13-G1v18**	**CH3**<br>**R1,**<br>**G109,**<br>**Y120**	CH3<br>**E**1>**R** (345),<br>**E**109>**G** (430),<br>**S**120>**Y** (440)<br>IgG1-005-RGY	E345R,<br>E430G,<br>S440Y	IGHG1 CH3<br>1.4-3 (341-347)<br>GQPREPQ ><br>GQPRRPQ<br>FG 105-117 (426-437)<br>SVMHEA.LHNHYT ><br>SVMHGA.LHNHYT<br>G 118-125 (438-445)<br>QKSLSLSP ><br>QKYLSLSP	**CDC enhancement**.<br>Increases C1q binding [113].<br>The 4,10,13-G1v18 variant (CH3 R1, G109, Y120) forms IgG1 hexamers [116].	4,10,13-G1v18 includes 10,13-G1v98 (CH3 R1) which favors IgG1 hexamerization [116].	0
**039**	**Homsap**	**5-G1v19**	**CH2**<br>**A34**	CH2<br>**D**34>**A** (270)	D270A	IGHG1 CH2<br>34-41 (270-277)<br>DPEVKFNW ><br>APEVKFNW	**CDC reduction**.<br>Reduces C1q binding [103].		0

040	**Homsap**	**5-G1v20**	**CH2**<br>**A105**	CH2<br>K105>A (322)	K322A	IGHG1 CH2<br>FG 105-117 (322-332)<br>KVSNKA..LPAPI ><br>AVSNKA..LPAPI	**CDC reduction**.<br>Reduces C1q binding [103], [105].		8<br>*nivatrotamab 11591 (124,86)...*
041	**Homsap**	**9-G1v21**	**CH2**<br>**Y15.1,**<br>**T16,**<br>**E18**	CH2<br>M15.1>Y (252),<br>S16>T (254),<br>T18>E (256)<br>YTE	M252Y,<br>S254T,<br>T256E	IGHG1 CH2<br>13-18 (249-256)<br>DTLMI.SRT ><br>DTLYI.TRE	**Half-life increase.**<br>Enhances FCGRT binding at pH 6.0 [117], [118], [119].		**3D**<br>4n0f, 4n0 [120].<br>34<br>*netakimab 10387 (118,80)...*
042	**Homsap**	**9-G1v22**	**CH2**<br>**Y15.1,**<br>**T16,**<br>**E18**<br>**CH3**<br>**K113,**<br>**F114,**<br>**H116**	CH2<br>M15.1>Y (252),<br>S16>T (254),<br>T18>E (256)<br>CH3<br>H113>K (433),<br>N114>F (434),<br>Y116>H (436)	M252Y,<br>S254T,<br>T256E<br><br>H433K,<br>N434F,<br>Y436H	IGHG1 CH2<br>13-18 (249-256)<br>DTLMI.SRT ><br>DTLYI.TRE<br>IGHG1 CH3<br>FG 105-117 (426-437)<br>SVMHEA.LHNHYT ><br>SVMHEA.LKFHHT	**Half-life increase.**<br>Enhances FCGRT binding at pH 6.0 [117].		0
043	**Homsap**	**9-G1v22-1**	**CH2**<br>**Y15.1,**<br>**T16,**<br>**E18**<br>**CH3**<br>**K113,**<br>**F114**	CH2<br>M15.1>Y (252),<br>S16>T (254),<br>T18>E (256)<br>CH3<br>H113>K (433),<br>N114>F (434),	M252Y,<br>S254T,<br>T256E<br><br>H433K,<br>N434F	IGHG1 CH2<br>13-18 (249-256)<br>DTLMI.SRT ><br>DTLYI.TRE<br>IGHG1 CH3<br>FG 105-117 (426-437)<br>SVMHEA.LHNHYT ><br>SVMHEA.LKFHYT	**Reduces endogenous IG half-life owing to FCGRT blockade.**<br>Enhances FCGRT binding with reduced pH dependency (binds at near neutral pH and at pH 7.4 or so) [121].	FcRn blockers (or 'Abdegs,' for antibodies that enhance IgG degradation) [121].	1<br>*efgartigimod alfa 10455 (116,78).*
044	**Homsap**	**6-G1v23**	**CH2**<br>**E1.2**	CH2<br>L1.2>E (235)	L235E	IGHG1 CH2<br>1.6-3 (231-239)<br>APELLGGPS ><br>APELEGGPS	**ADCC reduction**.<br><br>Reduces FcγR binding [122], [123].	**CDC reduction**.<br>Reduces C1q binding [122], [123].	0
045	**Homsap**	**9-G1v24**	**CH3**<br>**L107,**<br>**S114**	CH3<br>M107>L (428),<br>N114>S (434)	M428L,<br>N434S	IGHG1 CH3<br>FG 105-117 (426-437)<br>SVMHEA.LHNHYT ><br>SVLHEA.LHSHYT	**Half-life increase.**<br>Enhances FCGRT binding at pH 6.0 (11-fold increase in affinity) [124].		20<br>*elipovimab 10988 (120,82)...*

**046**	**Homsap**	**9-G1v24-1**	**CH3**<br>**V107**	CH3<br>M107>V (428),	M428V	IGHG1 CH3<br>FG 105-117 (426-437)<br>SVMHEA.LHNHYT ><br>SVVHEA.LHNHYT	**Half-life increase.**<br>Enhances FCGRT binding at pH 6.0		**1**<br>*gimsilumab 10534 (117,79).*
**047**	**Homsap**	**7-G1v25**	**CH2**<br>**E29,**<br>**F113**	CH2<br>S29>E (267),<br>L113>F (328)<br>SELF	S267E,<br>L328F	IGHG1 CH2<br>23-31 (261-269)<br>CVVVDVSHE ><br>CVVVDVEHE<br>FG 105-117 (322-332)<br>KVSNKA..LPAPI ><br>KVSNKA..FPAPI	**FcγRIIB increase binding.**<br>Increases FcγRIIB binding (400-fold) [125]. Inhibits by downstream ITIM signaling in B cells [126].	Increases FcγRIIA (R131) binding affinity [125].binding affinity	**1**<br>*obexilimab 10898 (119,81).*
**048**	**Homsap**	**14-G1v26**	**CH3**<br>**Y22**	CH3<br>T22>Y (366)	T366Y	IGHG1 CH3<br>20-26 (364-370)<br>SLTCLVK ><br>SLYCLVK	**Knob (Y22)** of knobs-into-holes (KIH) which promotes the heterodimerization of the CH3 of two different H-gamma1 chains. The hole is 14-G1v31 [127].		0
**049**	**Homsap**	**16-G1v27**	**CH2**<br>**C3**	CH2<br>S3>C (329)	S239C	IGHG1 CH2<br>1.6-4 (231-240)<br>APELLGGPSV ><br>APELLGGPCV	**Conjugation site-specific.**<br>Site-specific drug attachment engineered cysteine.		**6**<br>*vadastuximab talirine 9983 (113,75)…*
**050**	**Homsap**	**16-G1v27-1**	**CH1**<br>**C1.4**	CH1<br>A1.4>C (118)	A118C	IGHG1 CH1<br>1.4-5 (118-126)<br>ASTKGPSVF ><br>CSTKGPSVF	**Conjugation site-specific.**<br>Site-specific drug attachment engineered cysteine.		**2**<br>*iladatuzumab 10646 (117,!)…*
**051**	**Homsap**	**16-G1v28**	**CH2**<br>**C(3^4)**	CH2<br>(3^4)C(239^240)	C(239^240)	IGHG1 CH2<br>1.6-4 (231-240)<br>APELLGGPSV ><br>APELLGGPSCV	**Conjugation site-specific.**<br>Site-specific drug attachment engineered cysteine.		**1**<br>*idactamab 11348 (123,85).*
**052**	**Homsap**	**8-G1v29**	**CH2**<br>**A84.4**	CH2<br>N84.4>A (297)	N297A	IGHG1 CH2<br>83-88 (292-305)<br>REEQYNSTYRVVSV ><br>REEQYASTYRVVSV	**ADCC reduction.**<br>Reduces FcγR binding [128].	Owing to the absence of N-glycosylation at CH2 84.4 [129].	**33**<br>*otelixizumab 8864 (98,60)…*
**053**	**Homsap**	**8,5-G1v29-20**	**CH2**<br>**A84.4**<br>**A105**	CH2<br>N84.4>A (297)<br>K105>A (322)	N297A<br>K322A	IGHG1 CH2<br>83-88 (292-305)<br>REEQYNSTYRVVSV ><br>REEQYASTYRVVSV<br>FG 105-117 (322-332)	**ADCC reduction.**<br>Reduces FcγR binding, owing to the absence of N-glycosylation at CH2 84.4 (8-G1v29).	**CDC reduction.**<br>Reduces C1q binding (5-G1v20).	Combines 8-G1v29 and 5-G1v20.<br>0

						KVSNKA..LPAPI > AVSNKA..LPAPI			
**054**	**Homsap**	**8-G1v30**	**CH2** **G84.4**	CH2 N84.4>G (297)	N297G	IGHG1 CH2 83-88 (292-305) REEQYNSTYRVVSV > REEQYGSTYRVVSV	**ADCC reduction**. Reduces FcγR binding [130].	Owing to the absence of N-glycosylation at CH2 84.4 [129].	**7** *mosunetuzumab 10621 (117,79)...*
**055**	**Homsap**	**8-G1v30-1**	**CH2** **H84.4**	CH2 N84.4>H (297)	N297H	IGHG1 CH2 83-88 (292-305) REEQYNSTYRVVSV > REEQYHSTYRVVSV	**ADCC reduction**. Reduces FcγR binding.	Owing to the absence of N-glycosylation at CH2 84.4 [129].	**1** *semzuvolimab 11685 (126,88).*
**056**	**Homsap**	**14-G1v31**	**CH3** **T86**	CH3 Y86>T (407)	Y407T	IGHG1 CH3 85.4-92 (402-413) GSFFLYSKLTVD > GSFFLTSKLTVD	**Hole (T86)** of knobs-into-holes (KIH) which promotes the heterodimerization of the CH3 of two different H-gamma1 chains. The knob is 14-G1v26 [127].		0
**057**	**Homsap**	**14-G1v31-1**	**CH3** **A86**	CH3 Y86>A (407)	A407T	IGHG1 CH3 85.4-92 (402-413) GSFFLYSKLTVD > GSFFLASKLTVD	**Hole (A86)** of knobs-into-holes (KIH) promotes the heterodimerization of the CH3 of two different H-gamma1 chains. The knob is 14-G1v32 [131].		**1** *zeclantamig 13865 (134,).*
**058**	**Homsap**	**14-G1v32**	**CH3** **W22**	CH3 T22>W (366)	T366W	IGHG1 CH3 20-26 (364-370) SLTCLVK > SLWCLVK	**Knob (W22)** of knobs-into-holes (KIH) which promotes the heterodimerization of the CH3 of two different H-gamma1 chains. The hole is 14-G1v33 or 14-G1v31-1 [131].		**60** *onartuzumab 9368 (104,66)…*
**059**	**Homsap**	**14-G1v32-1**	**CH3** **M3,** **F5,** **D6,** **W22,** **M24**	CH3 Q3>M (347), Y5>F (349), T6>D (350), T22>W (366), L24>M (368)	Q347M, Y349F, T350D, T366W, L368M	IGHG1 CH3 1.4-7 (341-351) GQPREPQVYTL > GQPREPMVFDL > 20-26 (364-370) SLTCLVK > SLWCMVK	**Knob** (W22) of knobs-into-holes (KIH) including associated aa changes (M3, F5, D6, M24) which can form a **salt bridge** to enhance the heterodimerization of the CH3 of two different H-gamma1 chains. The hole is 14-G1v33-1.		**1** *remigromig 13973 (134,).*
**060**	**Homsap**	**14-G1v33**	**CH3** **S22,** **A24,** **V86**	CH3 T22>S (366), L24>A (368), Y86>V (407)	T366S, L368A, Y407V	IGHG1 CH3 20-26 (364-370) SLTCLVK > SLSCAVK 85.4-92 (402-413) GSFFLYSKLTVD > GSFFLVSKLTVD	**Hole (SAV)** of knobs-into-holes (KIH) which promotes the heterodimerization of the CH3 of two different H-gamma1 chains. The knob is 14-G1v32 [131].		**59** *onartuzumab 9368 (104,66)…*

**061**	**Homsap**	**14-G1v33-1**	**CH3**<br>**I10,**<br>**L13 ,**<br>**S16,**<br>**S22,**<br>**A24,**<br>**V86**	CH3<br>S10>I (354),<br>E13>L (357),<br>K16>S (360),<br>T22>S (366),<br>L24>A (368),<br>Y86>V (407)	S354I,<br>E357L,<br>K360S,<br>T366S,<br>L368A,<br>Y407V	IGHG1 CH3<br>8-19 (352-363)<br>PPSRDELT…KNQV ><br>PPIRDLLT…SNQV><br>20-26 (364-370)<br>SLTCLVK ><br>SLSCAVK<br>85.4-92 (402-413)<br>GSFFLYSKLTVD ><br>GSFFLVSKLTVD	**Hole** (SAV) of knobs-into-holes (KIH) and associated amino acid changes (I10, L13, S16) which can form a **salt bridge** to enhance the heterodimerization of the CH3 of two different H-gamma1 chains. The knob and associated aa changes is 14-G1v32-1.		**1**<br>*remigromig 13973 (134,).*
**062**	**Homsap**	**10,13-G1v34**	**CH3**<br>**G109**	CH3<br>E109>G (430)	E430G	IGHG1 CH3<br>FG 105-117 (426-437)<br>SVMHEA.LHNHYT ><br>SVMHGA.LHNHYT	**Hexamerization**.<br>Favors IgG1 hexamerization by increased intermolecular Fc-Fc interactions after antigen binding on the cell surface.		**4**<br>*benufatamab 11063 (121,83)…*
**063**	**Homsap**	**4-G1v35**	**CH2**<br>**E29**	CH2<br>S29>E (267)<br>SE	S267E	IGHG1 CH2<br>23-31 (261-269)<br>CVVVDVSHE ><br>CVVVDVEHE	**CDC enhancement**.<br>Increases C1q binding [115].	Binds to FCGRT and FcγRIIB, but not to other FcγR in a mouse model [132].	**2**<br>*sotigalimab 11472 (123,85)*<br>*exlinkibart 12376 (127,89).*
**064**	**Homsap**	**8-G1v36**	**CH2**<br>**Q84.4**	CH2<br>N84.4>Q (297)	N297Q	IGHG1 CH2<br>83-88 (292-305)<br>REEQYN..STYRVVSV ><br>REEQYQ..STYRVVSV	**ADCC reduction**.<br>Reduces FcγR binding [129], [130], [133].	Owing to the absence of N-glycosylation at CH2 84.4 [129], [130], [133].	**4**<br>*mupadolimab 11944 (125,87)…*
**065**	**Homsap**	**8-G1v36-1**	**CH2**<br>**S84.4**	CH2<br>N84.4>S (297)	N297S	IGHG1 CH2<br>83-88 (292-305)<br>REEQYN..STYRVVSV ><br>REEQYS..STYRVVSV	**ADCC reduction**.<br>Reduces FcγR binding [130].	Owing to the absence of N-glycosylation at CH2 84.4.	**1**<br>*eftozanermin alfa* (FPIA) *10838 (119,81).*
**066**	**Homsap**	**15-G1v37**	**hinge**<br>**S5**	hinge<br>C5>S (220)	C220S	IGHG1 hinge<br>1-15 (216-230)<br>EPKSCDKTHTCPPCP ><br>EPKSSDKTHTCPPCP	No disulfide bridge inter H-L.		**46**<br>*placulumab 9567 (107,69)…*
**067.1**	**Homsap**	**15-G1v37-1**	**hinge**<br>**G5**	hinge<br>C5>G (220)	C220G	IGHG1 hinge<br>1-15 (216-230)<br>EPKSCDKTHTCPPCP ><br>EPKSGDKTHTCPPCP	No disulfide bridge inter H-L.		**1**<br>*tuvonralimab 11841 (125,87).*

**067.2**	**Homsap**	**15-G1v37-2**	**hinge**<br>**L5**	hinge<br>C5>L (220)	C220L	IGHG1 hinge<br>1-15 (216-230)<br>EPKSCDKTHTCPPCP ><br>EPKSLDKTHTCPPCP	No disulfide bridge inter H-L.		1<br>*efgivanermin alfa* (FPIA) *10695 (120,82)*
**068**	**Homsap**	**6-G1v38**	**CH2**<br>**S108,**<br>**F113**	CH2<br>N108>S (325),<br>L113>F (328)	N325S,<br>L328F	IGHG1 CH2<br>FG 105-117 (322-332)<br>KVSNKA..LPAPI ><br>KVSSKA..FPAPI	**ADCC reduction**. Abrogates FcγRIII binding, increases FcγRII binding, retains FcγRI high affinity binding [134].	**CDC reduction**. Abrogates C1q binding.	1 *paridiprubart 12233 (126,88)*<br>Increased potency of **neutralizing antibody** by coengagement of target and FcγRIIA [134].
**069**	**Homsap**	**6-G1v39**	**CH2**<br>**F1.3,**<br>**E1.2,**<br>**S116**	CH2<br>L1.3>F (234),<br>L1.2>E (235),<br>P116>S (331)<br>FES, TM,<br>LFLE-PS	L234F,<br>L235E,<br>P331S	IGHG1 CH2<br>1.6-3 (231-239)<br>APELLGGPS ><br>APEFEGGPS<br>FG 105-117 (322-332)<br>KVSNKA..LPAPI ><br>KVSNKA..LPASI	**ADCC reduction**. Reduces FcγR effector properties [135].	**CDC reduction.** Reduces C1q binding, P116>S [136].	**3D**<br>3c2s [135].<br>22<br>*anifrolumab 9800 (109,71)…*
**070**	**Homsap**	**6-G1v40**	**CH2**<br>**A1.3,**<br>**A1.2,**<br>**S116**	CH2<br>L1.3>A (234),<br>L1.2>A (235),<br>P116>S (331)	L234A,<br>L235A,<br>P331S	IGHG1 CH2<br>1.6-3 (231-239)<br>APELLGGPS ><br>APEAAGGPS<br>FG 105-117 (322-332)<br>KVSNKA..LPAPI ><br>KVSNKA..LPASI	**ADCC reduction**. Reduces FcγR binding.	**CDC reduction.** Reduces C1q binding, P116>S [136].	3<br>*amtabafusp alfa 13207 (131,93)...*
**071.1**	**Homsap**	**6-G1v41**	**CH2**<br>**F1.3,**<br>**E1.2**	CH2<br>L1.3>F (234),<br>L1.2>E (235)<br>FE	L234F,<br>L235E	IGHG1 CH2<br>1.6-3 (231-239)<br>APELLGGPS ><br>APEFEGGPS	**ADCC reduction**. Reduces FcγR binding [137].	**CDC reduction.** Reduces C1q binding [122].	0<br>part of 6-G1v41-66.
**071.2**	**Homsap**	**6-G1v41-1**	**CH2**<br>**F1.3**	CH2<br>L1.3>F (234)	L234F	IGHG1 CH2<br>1.6-3 (231-239)<br>APELLGGPS ><br>APEFLGGPS	**ADCC reduction**. Reduces FcγR binding.	**CDC reduction**. Reduces C1q binding.	0

**072**	**Homsap**	**6-G1v41-66**	**CH2**<br>**F1.3,**<br>**E1.2,**<br>**A27**	CH2<br>**L**1.3>**F** (234),<br>**L**1.2>**E** (235),<br>**D**27>**A** (265)<br>FEA	L234F,<br>L235E,<br>D265A	IGHG1 CH2<br>1.6-3 (231-239)<br>APELLGGPS ><br>APEFEGGPS<br>IGHG1 CH2<br>23-31 (269)<br>CVVVDVSHE ><br>CVVVAVSHE	**ADCC reduction**.<br>Reduces FcγR binding.	**CDC reduction**.<br>Reduces C1q binding.	Combines 6-G1v41 and 1-G1v66 (CH2 A27).<br>**4**<br>*epcoritamab 11078 (121,83)...*
**073**	**Homsap**	**9-G1v42**	**CH2**<br>**Q14**<br><br>**CH3**<br>**L107**	CH2<br>**T**14>**Q** (250)<br><br>CH3<br>**M**107>**L** (428)	T250Q<br><br><br>M428L	IGHG1 CH2<br>13-18 (249-256)<br>DTLMISRT ><br>DQLMISRT<br>CH3<br>FG 105-117 (426-437)<br>SVMHEA.LHNHYT ><br>SVLHEA.LHNHYT	**Half-life increase.**<br>Enhances FCGRT binding at pH 6.0 [117].		**3**<br>*elezanumab 10344 (115,77)...*
**074**	**Homsap**	**6-G1v43**	**CH2**<br>**A1.3,**<br>**E1.2,**<br>**A1**	CH2<br>**L**1.3>**A** (234),<br>**L**1.2>**E** (235),<br>**G**1>**A** (237)	L234A,<br>L235E,<br>G237A	IGHG1 CH2<br>1.6-3 (231-239)<br>APELLGGPS ><br>APEAEGAPS	**ADCC reduction**.<br>Reduces FcγR binding.	**CDC reduction**.<br>Reduces C1q binding.	**5**<br>*olamkicept 10311 (116,78)...*
**075**	**Homsap**	**6-G1v43-1**	**CH2**<br>**A1.3,**<br>**A1**	CH2<br>**L**1.3>**A** (234),<br>**G**1>**A** (237)<br>LAGA	L234A,<br>G237A	IGHG1 CH2<br>1.6-3 (231-239)<br>APELLGGPS ><br>APEALGAPS	**ADCC reduction**.<br>Reduces FcγR binding.	**CDC reduction**.<br>Reduces C1q binding.	**3**<br>*torapsel 8337 (91,53)…*
**076**	**Homsap**	**6-G1v43-2**	**CH2**<br>**A1.3,**<br>**E1.2**	CH2<br>**L**1.3>**A** (234),<br>**L**1.2>**E** (235)<br>LALE	L234A,<br>L235E	IGHG1 CH2<br>1.6-3 (231-239)<br>APELLGGPS ><br>APEAEGGPS	**ADCC reduction**.<br>Reduces FcγR binding.	**CDC reduction**.<br>Reduces C1q binding.	**1**<br>*foralumab 9309 (103,65)*
**077**	**Homsap**	**6-G1v43-2-49**	**CH2**<br>**A1.3,**<br>**E1.2,**<br>**G114**	CH2<br>**L**1.3>**A** (234),<br>**L**1.2>**E** (235),<br>**P**114>**G** (329)<br>LALEPG	L234A,<br>L235E,<br>P329G	IGHG1 CH2<br>1.6-3 (231-239)<br>APELLGGPS ><br>APEAEGGPS<br>FG 105-117 (322-332)<br>KVSNKA..LPAPI ><br>KVSNKA..LGAPI	**ADCC reduction**.<br>Reduces FcγR binding.	**CDC reduction**.<br>Reduces C1q binding.	Combines 6-G1v43-2 and 6-G1v49 (CH2 G114).<br>0

**078**	**Homsap**	**6-G1v43-60**	**CH2**<br>**A1.3,**<br>**E1.2,**<br>**A1,**<br>**S115,**<br>**S116**	CH2<br>**L**1.3>**A** (234),<br>**L**1.2>**E** (235),<br>**G**1>**A** (237),<br>**A**115>**S** (330),<br>**P**116>**S** (331)	L234A,<br>L235E,<br>G237A,<br>A330S,<br>P331S	IGHG1 CH2<br>1.6-3 (231-239)<br>APELLGGPS ><br>APEAEGAPS<br>FG 105-117 (322-332)<br>KVSNKA..LPAPI ><br>KVSNKA..LPSSI	**ADCC reduction**.<br>Reduces FcγR binding.	**CDC reduction**.<br>Reduces C1q binding.	Combines 6-G1v43 and 6-G1v60 (CH2 S115, S116).<br>**6**<br>*atacicept*<br>*8669 (95,57)…*
**079**	**Homsap**	**16-G1v44**	**CH3**<br>**C122**	CH3<br>**S**122>C (442)	S442C	IGHG1 CH3<br>118-125 (438-445)<br>QKSLSLSP ><br>QKSLCLSP	**Conjugation site-specific**.<br>Site-specific drug attachment engineered cysteine.		**4**<br>*pivekimab*<br>*11693 (125,87)...*
**080**	**Homsap**	**3-G1v45**	**CH2**<br>**A1.1,**<br>**L115,**<br>**E117**	CH2<br>**G**1.1>**A** (236),<br>**A**115>**L** (330),<br>**I**117>**E** (332)<br>GAALIE	G236A,<br>A330L,<br>I332E	IGHG1 CH2<br>1.6-3 (231-239)<br>APELLGGPS ><br>APELLAGPS<br>FG 105-117 (322-332)<br>KVSNKA..LPAPI ><br>KVSNKA..LPLPE	**ADCC enhancement**.<br>Increases FcγIIIA binding [138].	**ADCP enhancement**.<br>NK cell activation [138].	**2**<br>*beludavimab 11961 (125,87).*<br>*tobevibart*<br>*12316 (127,89)*
**081**	**Homsap**	**9-G1v46**	**CH3**<br>**K113,**<br>**F114**	CH3<br>**H**113>**K** (433),<br>**N**114>**F** (434)	H433K,<br>N434F	IGHG1 CH3<br>FG 105-117 (426-437)<br>SVMHEA.LHNHYT ><br>SVMHEA.LKFHYT	**Half-life increase.**<br>Enhances FCGRT binding at pH 6.0 (with very little binding at pH7.4) [139], [140].		**1**<br>*empasiprubart 12277 (127,89).*
**082**	**Homsap**	**1-G1v47**	**CH2**<br>**delG1.1**	CH2<br>**G**1.1>del (236)	G236del	IGHG1 CH2<br>1.6-3 (231-239)<br>APELLGGPS ><br>APELL - GPS	**ADCC reduction**.<br>Eliminates binding to FcγRI, FcγRIIA, FcγRIIIA [141].		**2** *exlinkibart 12376 (127,89), dalnicastobart 12377 (127,89).*
**083**	**Homsap**	**6-G1v48**	**CH2**<br>**R113**	CH2<br>**L**113>**R** (328)	L328R	IGHG1 CH2<br>FG 105-117 (322-332)<br>KVSNKA..LPAPI ><br>KVSNKA..RPAPI	**ADCC reduction**.<br>Reduces FcγR binding.	**CDC reduction**.<br>Reduces C1q binding.	0
**084**	**Homsap**	**6-G1v49**	**CH2**<br>**G114**	CH2<br>**P**114>**G** (329)	P329G	IGHG1 CH2<br>FG 105-117 (322-332)<br>KVSNKA..LPAPI ><br>KVSNKA..LGAPI	**ADCC reduction**.<br>Reduces FcγR binding [114].	**CDC reduction**.<br>Reduces C1q binding [114].	0

**085**	**Homsap**	**1-G1v50**	**CH2**<br>**delE1.4**<br>**P1.3,**<br>**V1.2,**<br>**A1.1**	CH2<br>E1.4>del (233),<br>L1.3>P (234),<br>L1.2>V (235),<br>G1.1>A (236)	E233del,<br>L234P,<br>L235V,<br>G236A	IGHG1 CH2<br>1.6-3 (231-239)<br>APELLGGPS ><br>AP-PVAGPS<br>(G2-like)	**ADCC reduction**.<br>Decreases FcgammaR binding.	In literature, search for :<br>E233P,L234V,L235 A,G236del	4<br>*precemtabart tocentecan 13008 (130,92)...*
**086**	**Homsap**	**1-G1v50-1**	**CH2**<br>**delE1.4**<br>**P1.3,**<br>**A1.2,**<br>**A1.1**	CH2<br>E1.4>del (233),<br>L1.3>P (234),<br>L1.2>A (235),<br>G1.1>A (236)	E233del,<br>L234P,<br>L235A,<br>G236A	IGHG1 CH2<br>1.6-3 (231-239)<br>APELLGGPS ><br>AP-PAAGPS	**ADCC reduction**.<br>Decreases FcgammaR binding.		0
**087**	**Homsap**	**6-G1v50-1-4**	**CH2**<br>**delE1.4**<br>**P1.3,**<br>**A1.2,**<br>**A1.1**<br>**A114**	CH2<br>E1.4>del (233),<br>L1.3>P (234),<br>L1.2>A (235),<br>G1.1>A (236)<br>P114>A (329)	E233del,<br>L234P,<br>L235A,<br>G236A<br>P329A	IGHG1 CH2<br>1.6-3 (231-239)<br>APELLGGPS ><br>AP-PAAGPS<br>FG 105-117 (322-332)<br>KVSNKA..LPAPI ><br>KVSNKA..LAAPI	**ADCC reduction**.<br>Decreases FcgammaR binding.	**CDC reduction**.<br>Reduces C1q binding.	Combines 1-G1v50-1 (CH2 delE1.4, P1.3, A1.2, A1.1) and 6-G1v4 (CH2 A114).<br>2 *garivulimab 11451 (123,85), surzebiclimab 11453 (124,86).*
**088**	**Homsap**	**6-G1v50-4**	**CH2**<br>**delE1.4**<br>**P1.3,**<br>**V1.2,**<br>**A1.1,**<br>**A114**	CH2<br>E1.4>del (233),<br>L1.3>P (234),<br>L1.2>V (235),<br>G1.1>A (236),<br>P114>A (329)	E233del,<br>L234P,<br>L235V,<br>G236A,<br>P329A	IGHG1 CH2<br>1.6-3 (231-239)<br>APELLGGPS ><br>AP-PVAGPS (G2-like)<br>FG 105-117 (322-332)<br>KVSNKA..LPAPI ><br>KVSNKA..LAAPI	**ADCC reduction**.<br>Decreases FcgammaR binding.	**CDC reduction**.<br>Reduces C1q binding.	Combines 1-G1v50 (CH2 delE1.4, P1.3, V1.2, A1.1) (G2-like) and 6-G1v4 (CH2 A114).<br>0
**089**	**Homsap**	**6-G1v50-51**	**CH2**<br>**delE1.4**<br>**P1.3,**<br>**V1.2,**<br>**A1.1,**<br>**K29**	CH2<br>E1.4>del (233),<br>L1.3>P (234),<br>L1.2>V (235),<br>G1.1>A (236),<br>S29>K (267)	E233del,<br>L234P,<br>L235V,<br>G236A,<br>S267K	IGHG1 CH2<br>1.6-3 (231-239)<br>APELLGGPS ><br>AP-PVAGPS (G2-like)<br>23-31 (261-269)<br>CVVVDVSHE ><br>CVVVDVKHE	**ADCC reduction**.<br>Decreases FcgammaR binding.	**CDC reduction**.<br>Reduces C1q binding.	Combines 1-G1v50 (CH2 delE1.4, P1.3, V1.2, A1.1) (G2-like) and 6-G1v51 (CH2 K29).<br>9 *plamotamab 11020 (120,82)...*

**090**	**Homsap**	**6-G1v50-79**	**CH2**<br>**delE1.4**<br>**P1.3,**<br>**V1.2,**<br>**A1.1**<br>**G27**<br>**Q110**<br>**S115**	CH2<br>**E**1.4>**del** (233),<br>**L**1.3>**P** (234),<br>**L**1.2>**V** (235),<br>**G**1.1>**A** (236)<br>**D**27>**G** (265)<br>**A**110>**Q** (327)<br>**A**115>**S** (330)	E233del,<br>L234P,<br>L235V,<br>G236A<br>D265G<br>A327Q<br>A330S	IGHG1 CH2<br>1.6-3 (231-239)<br>APELLGGPS ><br>AP-PVAGPS (G2-like)<br>23-31 (261-269)<br>CVVVDVSHE ><br>CVVVGVSHE<br>FG 105-117 (322-332)<br>KVSNKA..LPAPI ><br>KVSNKQ..LPSPI	**ADCC reduction.**<br>Decreases FcgammaR binding.	**CDC reduction.**<br>Reduces C1q binding.	Combines 1-G1v50 (G2-like) and 1-G1v79 (CH2 G27, Q110, S115) [142].<br>**1**<br>*timcevibart 12929 (129,91)*
**091**	**Homsap**	**6-G1v51**	**CH2**<br>**K29**	CH2<br>**S**29>**K** (267)	S267K	IGHG1 CH2<br>23-31 (261-269)<br>CVVVDVSHE ><br>CVVVDVKHE	**ADCC reduction.**<br>Reduces FcγR binding.	**CDC reduction.**<br>Reduces C1q binding.	0<br>part of 6-G1v50-51.
**092**	**Homsap**	**1-G1v52**	**CH2**<br>**R1.1,**<br>**R113**	**G**1.1>**R** (236)<br>**L**113>**R** (328)<br>RR,<br>GRLR	G236R,<br>L328R	IGHG1 CH2<br>1.6-3 (231-239)<br>APELLGGPS ><br>APELLRGPS<br>FG 105-117 (322-332)<br>KVSNKA..LPAPI ><br>KVSNKA..RPAPI	**ADCC reduction.**<br>Abrogates FcγR binding.		0
**093**	**Homsap**	**6-G1v53**	**CH2**<br>**F1.3,**<br>**Q1.2,**<br>**Q105**	CH2<br>**L**1.3>**F** (234),<br>**L**1.2>**Q** (235),<br>**K**105> **Q** (322)<br>FQQ	L234F,<br>L235Q,<br>K322Q,	IGHG1 CH2<br>1.6-3 (231-239)<br>APELLGGPS ><br>APEFQGGPS<br>FG 105-117 (322-332)<br>KVSNKA..LPAPI ><br>QVSNKA..LPAPI	**ADCC reduction.**<br>Reduces FcγR binding [143].	**CDC reduction.**<br>Reduces C1q binding [143].	Associated with 9-G1v21 (CH2 Y15.1, T16, E18) in FQQ-YTE.<br>0
**094**	**Homsap**	**6-G1v53-1**	**CH2**<br>**A1.3,**<br>**Q1.2,**<br>**Q105**	**CH2**<br>L1.3>A (234),<br>L1.2>Q (235),<br>K105>Q (322)<br>AQQ	L234A,<br>L235Q,<br>K322Q	IGHG1 CH2<br>1.6-3 (231-239)<br>APELLGGPS ><br>APEAQGGPS<br>FG 105-117 (322-332)<br>KVSNKA..LPAPI ><br>QVSNKA..LPAPI	**ADCC reduction.**<br>Reduces FcγR binding.	**CDC reduction.**<br>Reduces C1q binding.	Associated with 9-G1v21 (CH2 Y15.1, T16, E18) in AQQ-YTE [144].<br>**1** *barzolvolimab 11842 (125,87)...*

**095**	**Homsap**	**11-G1v54**	**CH2**<br>**C83,**<br>**C85**	CH2<br>R83>C (292),<br>V85>C (302)	R292C,<br>V302C	IGHG1 CH2<br>83-88 (292-305)<br>REEQYN..STYRVVSV ><br>CEEQYN..STYRCVSV	Stabilizes CH2 in the absence of N84.4 (297) glycosylation owing to the expression system) [145].		1<br>*emzotamig 13697 (133,95).*
**096**	**Homsap**	**11,8-G1v54-29**	**CH2**<br>**C83,**<br>**A84.4,**<br>**C85**	CH2<br>R83>C (292),<br>N84.4>A (297),<br>V85>C (302)	R292C,<br>N297A,<br>V302C	IGHG1 CH2<br>83-88 (292-305)<br>REEQYN..STYRVVSV ><br>CEEQYA..STYRCVSV	Stabilizes CH2 in the absence of N84.4 (297) glycosylation (11-G1v54).	**ADCC reduction**. Reduces FcγR binding (8-G1v29) [130].	Combines Homsap 11-G1v54 and 8-G1v29.<br>0
**097**	**Homsap**	**11,8-G1v54-30**	**CH2**<br>**C83,**<br>**G84.4,**<br>**C85**	CH2<br>R83>C (292),<br>N84.4>G (297),<br>V85>C (302)	R292C,<br>N297G,<br>V302C	IGHG1 CH2<br>83-88 (292-305)<br>REEQYN..STYRVVSV ><br>CEEQYG..STYRCVSV	Stabilizes CH2 in the absence of N84.4 (297) glycosylation (11-G1v54) [145].	**ADCC reduction**. Reduces FcγR binding (8-G1v30) [130].	Combines Homsap 11-G1v54 and 8-G1v30.<br>**14** *emerfetamab 11239 (123,85)...*
**098**	**Homsap**	**11,8-G1v54-36**	**CH2**<br>**C83,**<br>**Q84.4,**<br>**C85**	CH2<br>R83>C (292),<br>N84.4>Q (297),<br>V85>C (302)	R292C,<br>N297Q,<br>V302C	IGHG1 CH2<br>83-88 (292-305)<br>REEQYN..STYRVVSV ><br>CEEQYQ..STYRCVSV	Stabilizes CH2 in the absence of N84.4 (297) glycosylation (11-G1v54).	**ADCC reduction**. Reduces FcγR binding (8-G1v36) [130].	Combines Homsap 11-G1v54 and 8-G1v36.<br>0
**099**	**Homsap**	**16-G1v55**	**CH3**<br>**C123**	CH3<br>L123>C (443)	L443C	IGHG1 CH3<br>118-125 (438-445)<br>QKSLSLSP ><br>QKSLSCSP	**Conjugation site-specific**.<br>Site-specific drug attachment engineered cysteine.		**4**<br>*tarcocimab 11770 (129,90)...*
**100**	**Homsap**	**16-G1v56**	**CH1**<br>**F85.2(pAMF)**<br><br>**CH3**<br>**F85.2(pAMF)**	CH1<br>Y85.2>F(pAMF) (180)<br><br>CH3<br>F85.2>F(pAMF) (404)	Y180F(pAMF)<br><br>F404F(pAMF)	IGHG1 CH1<br>84.1-85.1 (174-181)<br>LQSS..GLYS ><br>LQSS..GLFS (F=pAMF)<br>CH3<br>84.1-85.1 (398-405)<br>LDSD..GSFF ><br>LDSD..GSFF (F=pAMF)	**Modified phenylalanine for conjugation.**<br>Non-natural amino acid, *para*-azidomethyl-L-phenylalanine (pAMF) for conjugation [146] (the antibody is produced in *Escherichia coli*, non glycosylated).		**4**<br>*luveltamab tazevibulin 12040 (126,88)...*
**101**	**Homsap**	**16-G1v56-1**	**CH1**<br>**F1.4(pAF)**	CH1<br>A1.4>F(pAF) (118)	A118F(pAF)	IGHG1 CH1<br>1.4-15 (118-136)<br>ASTKGPSVFPLAPSSKSTS ><br>FSTKGPSVFPLAPSSKSTS (F=pAF)	**Modified phenylalanine for conjugation.**<br>Non-natural amino acid, para-acetyl-phenylalanine (two acetylgroups on C-4 of Phe) (pAF) for conjugation.		**2** *anvatabart pactil 12271 (127,89), anvatabart opadotin 12272 (127,89).*

**102**	**Homsap**	**16-G1v56-2**	**CH3**<br>**F85.2(pAMF)**	CH3<br>F85.2>F(pAMF)<br>(404)	F404F(pAMF)	CH3<br>84.1-85.1 (398-405)<br>LDSD..GSFF ><br>LDSD..GSFF (F=pAMF)	**Modified phenylalanine for conjugation.** Non-natural amino acid, para-azidomethyl-l-phenylalanine (pAMF) for conjugation	2 *bezetabart 12653 (129,91), bezetabart debotansine 12652 (129,91).*
**103**	**Homsap**	**17-G1v57**	**CH1**<br>**E26,**<br>**E119**	CH1<br>K26>E (147),<br>K119>E (213)	K147E,<br>K213E	IGHG1 CH1<br>23-30 (144-151)<br>CLVKDYFP><br>CLVEDYFP<br>118-121 (212-215)<br>DKKV ><br>DEKV	Enhances, with 17-KCv57, the heteropairing H-L of bispecific G1-kappa antibodies.	9 *glofitamab 11145 (121,83)...*
**104**	**Homsap**	**17-G1v57-1**	**CH1**<br>**K84.2**	CH2<br>Q84.2>K	Q175K	IGHG1 CH1<br>83-88 (172-185)<br>AVLQSS..GLYSLSSV ><br>AVLKSS..GLYSLSSV	Enhances, with 17-KCv57-1 (S20>E, T90>E), the heteropairing H-L-kappa of bispecific G1-kappa/lambda antibodies.	0
**105**	**Homsap**	**17-G1v57-2**	**CH1**<br>**E26,**<br>**E84.2**	CH1<br>K26>E,<br>Q84.2>E	K147E<br>Q175E	IGHG1 CH1<br>23-30 (144-151)<br>CLVKDYFP ><br>CLVEDYFP<br>IGHG1 CH1<br>83-88 (172-185)<br>AVLQSS..GLYSLSSV ><br>AVLESS..GLYSLSSV	Enhances, with 17-LC2v57-2 (T20>K, S90>E), the heteropairing H-L-lambda of bispecific G1-kappa/lambda antibodies.	0
**106**	**Homsap**	**17-G1v57-3**	**CH1**<br>**D26**	CH1<br>K26>D (147)	K147D	IGHG1 CH1<br>23-30 (144-151)<br>CLVKDYFP><br>CLVDDYFP	Enhances, with 17-KCv57-3 (K20), the heteropairing H-L of bispecific G1-kappa antibodies.	1 *tuvonralimab 11841 (125,87).*
**107**	**Homsap**	**17-G1v58**	**CH1**<br>**C5**<br><br>**hinge**<br>**V5**	CH1<br>F5>C (126),<br><br>hinge<br>C5>V (220)	F126C,<br><br><br>C220V	IGHG1 CH1<br>1.4-15 (118-136)<br>ASTKGPSVFPLAPSSKSTS ><br>ASTKGPSVCPLAPSSKSTS<br>IGHG1 hinge<br>1-15 (216-230)<br>EPKSCDKTHTCPPCP ><br>EPKSVDKTHTCPPCP	Promotes, with 17-LC2v58 C10, the heteropairing H-L of bispecific G1-lambda antibodies by an alternative interchain cysteine mutations which create a novel disulfide bridge H CH1 C5 – L C10 instead of hinge C5 – L C126) [147].	5 *volrustomig 12168 (127,89)...*
**108**	**Homsap**	**17-G1v58-1**	**CH1**<br>**C5,**	CH1<br>F5>C (126),	F126C,	IGHG1 CH1<br>1.4-15 (118-136)	Promotes, with 17-LC2v58-1 R5, the heteropairing H-L of bispecific G1-lambda2	8tjf<br>0

			D20,<br>**hinge**<br>V5	A20>D (141),<br>hinge<br>C5>V (220)	A141D,<br><br>C220V	ASTKGPSVFPLAPSSKSTS ><br>ASTKGPSVCPLAPSSKSTS<br>CH1 16-26 (137-147)<br>GGTAALGCLVK ><br>GGTADLGCLVK<br>IGHG1 hinge<br>1-15 (216-230)<br>EPKSCDKTHTCPPCP ><br>EPKSVDKTHTCPPCP	antibodies by an alternative interchain cysteine mutations (see 17-G1v58) [147], which is enhanced by the 17-G1v58-1 CH1 D20 electrostatic steering interaction with 17-LC2v58-1 R5 [148].		
**109**	**Homsap**	**17-G1v58-2**	**CH1**<br>**C5,**<br>**E20,**<br>**hinge**<br>**V5**	CH1<br>F5>C (126),<br>A20>E (141),<br>hinge<br>C5>V (220)	F126C,<br>A141E,<br><br>C220V	IGHG1 CH1<br>1.4-15 (118-136)<br>ASTKGPSVFPLAPSSKSTS ><br>ASTKGPSVCPLAPSSKSTS<br>CH1 16-26 (137-147)<br>GGTAALGCLVK ><br>GGTAELGCLVK<br>IGHG1 hinge<br>1-15 (216-230)<br>EPKSCDKTHTCPPCP ><br>EPKSVDKTHTCPPCP	Promotes, with 17-LC2v58-1 R5, the heteropairing H-L of bispecific G1-lambda2 antibodies by an alternative interchain cysteine mutations (see 17-G1v58) [147], which is enhanced by the 17-G1v58-2 CH1 E20 electrostatic steering interaction with 17-LC2v58-1 R5 [148].		8tiu<br>0
110	**Homsap**	**6-G1v59**	**CH2**<br>**S1.3**<br>**T1.2,**<br>**R1.1**	CH2<br>L1.3>S (234),<br>L1.2>T (235),<br>G1.1>R (236)<br>STR	L234S,<br>L235T,<br>G236R	IGHG1 CH2<br>1.6-3 (231-239)<br>APELLGGPS ><br>APESTRGPS	**ADCC undetectable.**<br>Abrogates FcγR binding [149].	**CDC undetectable.**<br>Abrogates C1q binding [149].	0
111	**Homsap**	**6-G1v59-1**	**CH2**<br>**G1.2,**<br>**R1.1**	CH2<br>L1.2>G (235),<br>G1.1>R (236)<br>GR	L235G,<br>G236R	IGHG1 CH2<br>1.6-3 (231-239)<br>APELLGGPS ><br>APELGRGPS	**ADCC reduction.**<br>Reduces FcγR binding.	**CDC reduction.**<br>Reduces C1q binding.	2 *tepoditamab* 10766 *(118,80), besufetamig 13363 (132,94).*
112	**Homsap**	**6-G1v59-2**	**CH2**<br>**R1.2,**<br>**R1.1,**<br>**K3**	CH2<br>L1.2>R (235),<br>G1.1>R (236),<br>S3>K (239)<br>RRK	L235R,<br>G236R,<br>S239K	IGHG1 CH2<br>1.6-3 (231-239)<br>APELLGGPS ><br>APELRRGPK	**ADCC reduction.**<br>Reduces FcγR binding.	**CDC reduction.**<br>Reduces C1q binding.	1<br>*crovalimab 10882 (119,81).*

**113**	**Homsap**	**6-G1v60**	CH2<br>**S115,**<br>**S116**	CH2<br>A115>S (330)<br>**P**116>S (331)	A330S<br>P331S	IGHG1 CH2<br>FG 105-117 (322-332)<br>KVSNKA..LPAPI ><br>KVSNKA..LPSSI	**ADCC reduction**.<br>Reduces FcγR binding.	**CDC reduction**.<br>Reduces C1q binding, P116>S [136].	**4**<br>*precemtabart tocentecan 13008 (130,92)...*
**114**	**Homsap**	**6-G1v60-1**	CH2<br>**G110,**<br>**S115,**<br>**S116**	CH2<br>A110>G (327)<br>A115>S (330)<br>**P**116>S (331)	A327G<br>A330S<br>P331S	IGHG1 CH2<br>FG 105-117 (322-332)<br>KVSNKA..LPAPI ><br>KVSNKG..LPSSI	**ADCC reduction**.<br>Reduces FcγR binding.	**CDC reduction**.<br>Reduces C1q binding, P116>S [136].	**1**<br>*crovalimab 10882 (119,81).*
**115**	**Homsap**	**15-G1v61**	**hinge**<br>**S11**	hinge<br>C11>S (226)	C226S	IGHG1 hinge<br>1-15 (216-230)<br>EPKSCDKTHTCPPCP ><br>EPKSCDKTHTSPPCP	No disulfide bridge inter H-H h 11.		0
**116**	**Homsap**	**15-G1v62**	**hinge**<br>**S14**	hinge<br>C14>S (229)	C229S	IGHG1 hinge<br>1-15 (216-230)<br>EPKSCDKTHTCPPCP ><br>EPKSCDKTHTCPPSP	No disulfide bridge inter H-H h 14.		0
**117**	**Homsap**	**6-G1v63**	**CH2**<br>**S2**	CH2<br>**P**2>S (238)	P238S	IGHG1 CH2<br>1.6-3 (231-239)<br>APELLGGPS ><br>APELLGGSS	**ADCC reduction**.<br>Reduces FcγR binding.	**CDC reduction**.<br>Reduces C1q binding.	**4**<br>*abatacept 8495 (91,53)…*
**118**	**Homsap**	**6,7-G1v63-2**	**CH2**<br>**D2**	CH2<br>**P**2>D (238)	P238D	IGHG1 CH2<br>1.6-3 (231-239)<br>APELLGGPS ><br>APELLGGDS	**ADCC reduction**.<br>Reduces binding to activating FcγR [150].	**CDC reduction**.<br>Reduces C1q binding [150].	**FcγRIIB increase binding.**<br>Inhibits by downstream ITIM signaling in B cells [150].<br>**3D** 3wjj [151]<br>**1** *emugrobart 13238 (131,93).*
**119**	**Homsap**	**6,7-G1v63-3**	**CH2**<br>**D1.4,**<br>**D1,**<br>**D2,**	CH2<br>E1.4>D (233)<br>G1>D (237)<br>**P**2>D (238),	E233D,<br>G237D<br>P238D,	IGHG1 CH2<br>1.6-3 (231-239)<br>APELLGGPS ><br>APDLLGDDS	**ADCC reduction**.<br>Reduces binding to activating FcγR [150].	**CDC reduction**.<br>Reduces C1q binding [150].	**FcγRIIB increase binding.**<br>Inhibits by downstream ITIM

			**D30,**<br>**G35,**<br>**R115**	H30>D (268),<br>P35>G (271),<br>A115>R (330)<br>V12	H268D,<br>P271G,<br>A330R	23-31 (261-269)<br>CVVVDVSHE ><br>CVVVDVSDE<br>34-41 (270-277)<br>DPEVKFNW<br>DGEVKFNW<br>FG 105-117 (322-332)<br>KVSNKA..LPAPI ><br>KVSNKA..LPRPI			signaling in B cells [150].<br>**3D**<br>3wjl [151].<br>0
**120**	**Homsap**	**16-G1v64**	**CH2**<br>**C36**	CH2<br>E36>C (272)	E272C	IGHG1 CH2<br>34-41 (270-277)<br>DPEVKFNW ><br>DPCVKFNW	**Conjugation site-specific.**<br>Site-specific drug attachment engineered cysteine.		**2**<br>*maridebart 12601 (128,90), maridebart cafraglutide 12602 (128,90).*
**121**	**Homsap**	**6-G1v65**	**CH2**<br>**delE1.4,**<br>**delL1.3,**<br>**delL1.2**	CH2<br>E1.4>**del** (233),<br>L1.3>del (234),<br>L1.2>del (235)	E233del,<br>L234del,<br>L235del	IGHG1 CH2<br>1.6-3 (231-239)<br>APELLGGPS ><br>AP- - - GGPS	**ADCC reduction.**<br>Reduces FcγR binding.	**CDC reduction.**<br>Reduces C1q binding.	**2**<br>*enristomig 12416 (129,91), ozekibart 12417 (128,90).*
**122**	**Homsap**	**1-G1v66**	**CH2**<br>**A27**	CH2<br>D27>A (265)	D265A	IGHG1 CH2<br>23-31 (261-269)<br>CVVVDVSHE ><br>CVVVAVSHE	**ADCC reduction.**<br>Reduces FcγR binding.		**4**<br>*benmelstobart 12205 (128,90)...*
**123**	**Homsap**	**1-G1v66-80**	**CH2**<br>**A27,**<br>**G116**	CH2<br>D27>A (265),<br>P116>G (331)	D265A,<br>P331G	IGHG1 CH2<br>23-31 (261-269)<br>CVVVDVSHE ><br>CVVVAVSHE<br>FG 105-117 (322-332)<br>KVSNKA..LPAPI ><br>KVSNKA..LPAGI	**ADCC reduction.**<br>Reduces FcγR binding.		Combines 1-G1v66 (CH2 A27) and G1v80 (CH2 G116).<br>**1** *envafolimab 10930 (120,82).*
**124**	**Homsap**	**1-G1v67**	**CH2**<br>**S27**	CH2<br>D27>S (265)	D265S	IGHG1 CH2<br>23-31 (261-269)<br>CVVVDVSHE ><br>CVVVSVSHE	**ADCC reduction.**<br>Reduces FcγR binding.		0

**125**	**Homsap**	**14-G1v68**	**CH3**<br>**V6,**<br>**L22,**<br>**L79,**<br>**W81**	CH3<br>**T**6>**V** (350),<br>**T**22>**L** (366),<br>**K**79>**L** (392),<br>**T**81>**W** (394)	T350V,<br>T366L,<br>K392L,<br>T394W	IGHG1 CH3<br>3-9 (347-353)<br>QVYTLPP ><br>QVYVLPP<br>20-26 (364-370)<br>SLTCLVK ><br>SLLCLVK<br>77-84 (390-397)<br>NYKTTPPV ><br>NYLTWPPV	Enhances, with 14-G1v69, the heterodimerization H-H of bispecific G1 antibodies [152].	Previously G1v46.	**5**<br>*zanidatamab 10997 (121,83)...*
**126**	**Homsap**	**17-G1v68-1**	**CH1**<br>**E7,**<br>**T26,**<br>**E84.2**	CH1<br>**L**7>**E** (128),<br>**K**26>**T** (147),<br>**Q**84.2>**E** (175)	L128E,<br>K147T,<br>Q175E	IGHG1 CH1<br>1.4-15 (118-136)<br>ASTKGPSVFPLAPSSKSTS ><br>ASTKGPSVFPEAPSSKSTS<br>23-30 (144-151)<br>CLVKDYFP ><br>CLVTDYFP<br>IGHG1 CH1<br>83-88 (172-185)<br>AVLQSS..GLYSLSSV ><br>AVLESS..GLYSLSSV	Enhances the heteropairing H_L-kappa of 17-G1v68-1 CH1 with 17-KCv68 (R20, G22, R86) L-kappa in bispecific G1-kappa/lambda antibodies.	Previously G1v44.	**1**<br>*reozalimab 11904 (126,88).*
**127**	**Homsap**	**14-G1v69**	**CH3**<br>**V6,**<br>**Y7,**<br>**A85.1,**<br>**V86**	CH3<br>**T**6>**V** (350),<br>**L**7>**Y** (351),<br>**F**85.1>**A** (405),<br>**Y**86>**V** (407)	T350V,<br>L351Y,<br>F405A,<br>Y407V	IGHG1 CH3<br>3-9 (347-353)<br>QVYTLPP ><br>QVYVYPP<br>85.4-92 (402-413)<br>GSFFLYSKLTVD ><br>GSFALVSKLTVD	Enhances, with 14-G1v68, the heterodimerization H-H of bispecific G1 antibodies [152].	Previously G1v47.	**5**<br>*zanidatamab 10997 (121,83)...*
**128**	**Homsap**	**17-G1v69-1**	**CH1**<br>**K86**	CH1<br>**S**86>**K** (183)	S183K	IGHG1 CH1<br>85.4-89 (178-186)<br>GLYSLSSVV ><br>GLYSLKSVV	Enhances the heteropairing of 17-G1v69-1 CH1 with 17-LC2v69 L-lambda in bispecific G1-kappa/lambda.	Previously G1v45.	**1**<br>*reozalimab 11904 (126,88).*

**129**	**Homsap**	**15-G1v70**	**hinge**<br>**S5,**<br>**S11,**<br>**S14**	hinge<br>C5>S (220),<br>C11>S (226),<br>C14>S (229)	C220S,<br>C226S,<br>C229S	IGHG1 hinge<br>1-15 (216-230)<br>EPKSCDKTHTCPPCP ><br>EPKSSDKTHTSPPSP	Combines 15-G1v37 (no H-L), 15-G1v61 (no H-H h11) and 15-G1v62 (no H-H h14).		4<br>*abatacept*<br>*8495 (91,53)…*
**130**	**Homsap**	**14-G1v72**	**CH3**<br>**D7,**<br>**E24**	CH3<br>L7>D (351),<br>L24>E (368)<br>DE	L351D,<br>L368E	IGHG1 CH3<br>3-9 (347-353)<br>QVYTLPP ><br>QVYTDPP<br>20-26 (364-370)<br>SLTCLVK ><br>SLTCEVK	Enhances, with 14-G1v73, the heterodimerization H-H (dubbed 'DEKK') of bispecific G1 antibodies [153].		5 *zenocutuzumab*<br>*10687*<br>*(117/118,79)…*
**131**	**Homsap**	**14-G1v72-1**	**CH3**<br>**E7,**<br>**L86,**<br>**V88**	CH3<br>L7>E (351),<br>Y86>L (407),<br>K88>V (409)	L351E,<br>Y407L,<br>K409V	IGHG1 CH3<br>3-9 (347-353)<br>QVYTLPP ><br>QVYTEPP<br>85.4-92 (402-413)<br>GSFFLYSKLTVD ><br>GSFFLLSVLTVD	Enhances, with 14-G1v73-1, the heterodimerization H-H of bispecific G1 antibodies.		1<br>*fidasimtamab*<br>*11670 (125,87).*
**132**	**Homsap**	**14-G1v72-2**	**CH3**<br>**D79,**<br>**D88**	CH3<br>K79>D (392),<br>K88>D (409)	K392D,<br>K409D	IGHG1 CH3<br>77-84 (390-397)<br>NYKTTPPV ><br>NYDTTPPV<br>85.4-92 (402-413)<br>GSFFLYSKLTVD ><br>GSFFLYSDLTVD	Enhances, with (a) 14-G1v73-2, or (b) 14-G1v73-5, the heterodimerization H-H of bispecific G1 antibodies [154].		**(a)**<br>1 *latikafusp*<br>*12098 (126,88).*<br>**(b)**<br>1 *zeclantamig 13865*<br>*(134,).*
**133**	**Homsap**	**14-G1v72-3**	**CH1**<br>**D114,**<br>**CH2**<br>**E84.2,**<br>**CH3**<br>**D44,**<br>**E97,**<br>**D100**	**CH1**<br>N114>D (208)<br>**CH2**<br>Q84.2>E (295)<br>**CH3**<br>N44>D (384)<br>Q97>E (418)<br>N100>D (421)	N208D,<br><br><br>Q295E,<br><br>N384D,<br>Q418E,<br>N421D	IGHG1 CH1<br>FG 105-117 (201-211)<br>NVNHKP..SNTKV ><br>NVNHKP..SDTKV<br>IGHG1 CH2<br>84.1-85.1 (294-301)<br>EQYNSTYR ><br>EEYNSTYR<br>IGHG1 CH3	Enhances, with a native G1 chain (CH1 N114, CH2 Q84.2, CH3 N44, Q97, N100), the heterodimerization H-H of bispecific G1 antibodies.		9<br>*plamotamab 11020*<br>*(120,82)…*

						39-45.4 (379-389)<br>VEWESNGQPEN ><br>VEWESDGQPEN<br>97-104 (418-425)<br>QQGNVFSC ><br>EQGDVFSC			
**134**	**Homsap**	**14-G1v72-4**	**CH3**<br>**H20**<br>**A85.1**	CH3<br>**S**20>**H** (364),<br>**F**85.1>**A** (405)	S364H,<br>F405A	IGHG1 CH3<br>20-26 (364-370)<br>SLTCLVK ><br>HLTCLVK<br>85.4-92 (402-413)<br>GSFFLYSKLTVD ><br>GSFALYSKLTVD	Enhances, with 14-G1v73-4, the heterodimerization H-H of bispecific G1 antibodies [155].		0
**135**	**Homsap**	**14-G1v72-5**	**CH3**<br>**E26,**<br>**D88,**<br>**E119**	CH3<br>**K**26>**E** (370)<br>**K**88>**D** (409)<br>**K**119>**E** (439)	K370E,<br>K409D,<br>K439E	IGHG1 CH3<br>20-26 (364-370)<br>SLTCLVK ><br>SLTCLVE<br>85.4-92 (402-413)<br>GSFFLYSKLTVD ><br>GSFFLYSDLTVD<br>118-125 (438-445)<br>QKSLSLSP ><br>QKSLSLSL	Enhances, with 14-G1v73-6, the heterodimerization H-H of bispecific G1 antibodies.		**1**<br>*aveltamig*<br>*13944* (134,)
**136**	**Homsap**	**14-G1v73**	**CH3**<br>**K7,**<br>**K22**	CH3<br>**L**7>**K** (351),<br>**T**22>**K** (366)<br>KK	L351K,<br>T366K	IGHG1 CH3<br>3-9 (347-353)<br>QVYTLPP ><br>QVYTKPP<br>20-26 (364-370)<br>SLTCLVK ><br>SLKCLVK	Enhances, with 14-G1v72, the heterodimerization H-H (dubbed 'DEKK') of bispecific G1 antibodies [153].		**5** *zenocutuzumab*<br>*10687*<br>*(117/118,79)…*
**137**	**Homsap**	**14-G1v73-1**	**CH3**<br>**L22,**<br>**R84.2**	CH3<br>**T**22>**L** (366),<br>**D**84.2>**R** (399)	T366L,<br>D399R	IGHG1 CH3<br>20-26 (364-370)<br>SLTCLVK ><br>SLLCLVK<br>83-88 (396-409)<br>PVLDSD..GSFFLYSK >	Enhances, with 14-G1v72-1, the heterodimerization H-H of bispecific G1 antibodies.		**1**<br>*fidasimtamab*<br>*11670 (125,87).*

						PVLRSD..GSFFLYSK			
**138**	**Homsap**	**14-G1v73-2**	**CH3**<br>**K12,**<br>**K84.2**	CH3<br>E12>K (356),<br>D84.2>K (399)	E356K,<br>D399K	IGHG1 CH3<br>7-15 (351-359)<br>LPPSREEMT ><br>LPPSRKEMT<br>83-88 (396-409)<br>PVLDSD..GSFFLYSK ><br>PVLKSD..GSFFLYSK	Enhances, with 14G1v72-2, the heterodimerization H-H of bispecific G1 antibodies [154].		**1**<br>*latikafusp 12098 (126,88).*
**139**	**Homsap**	**10-G1v73-3**	**linker**<br>**K2,**<br>**K7,**<br>**K12,**<br>**K17**			Linker 20-mer (1-20)<br>GKPGSGKPGSGKPGSGKPGS	Facilitates the purification of bispecific G1 antibodies with heterodimeric H-H having one scFv.		**1**<br>*emaretamig 13773 (133,95).*
**140**	**Homsap**	**14-G1v73-4**	**CH3**<br>**T5,**<br>**F81**	CH3<br>Y5>T (349),<br>T81>F (394)	Y349T,<br>T394F	IGHG1 CH3<br>3-9 (347-353)<br>QVYTLPP ><br>QVTTLPP<br>77-84 (390-397)<br>NYKTTPPV ><br>NYKTFPPV	Enhances, with 14-G1v72-4, the heterodimerization H-H of bispecific G1 antibodies [155].		0
**141**	**Homsap**	**14-G1v73-5**	**CH3**<br>**R24,**<br>**K84.2**	CH3<br>L24>R (368),<br>D84.2>K (399)	L368R,<br>D399K	IGHG1 CH3<br>20-26 (364-370)<br>SLTCLVK ><br>SLTCRVK<br>83-88 (396-409)<br>PVLDSD..GSFFLYSK ><br>PVLKSD..GSFFLYSK	Enhances, with 14-G1v72-2, the heterodimerization H-H of bispecific G1 antibodies.		**1**<br>*zeclantamig 13865 (134,)*
**142**	**Homsap**	**14-G1v73-6**	**CH3**<br>**K12,**<br>**K13,**<br>**K84.2**	CH3<br>D12>K (356),<br>E13>K (357),<br>D84.2>K (399)	D356K,<br>E357K,<br>D399K	IGHG1 CH3<br>7-15 (351-359)<br>LPPSRDELT ><br>LPPSRKKLT<br>83-88 (396-409)<br>PVLDSD..GSFFLYSK ><br>PVLKSD..GSFFLYSK	Enhances, with 14-G1v72-5, the heterodimerization H-H of bispecific G1 antibodies.		**1**<br>*aveltamig 13944 (134,)*

**143**	**Homsap**	**14-G1v74**	**CH3**<br>**C10**	CH3<br>S10>C (354)	S354C	IGHG1 CH3<br>1.4-12 (341-356)<br>GQPREPQVYTLPPSRD><br>GQPREPQVYTLPPCRD >	Promotes with 14-G1v75, by a disulfide bridge, the heterodimerization H-H of bispecific G1 antibodies [156].		44<br>*vanucizumab 9950 (111,73)…*
**144**	**Homsap**	**14-G1v75**	**CH3**<br>**C5**	CH3<br>Y5>C (349)	Y349C	IGHG1 CH3<br>1.4-12 (341-356)<br>GQPREPQVYTLPPSRD ><br>GQPREPQVCTLPPSRD >	Promotes with 14-G1v74, by a disulfide bridge, the heterodimerization H-H of bispecific G1 antibodies [156].		44<br>*vanucizumab 9950 (111,73)…*
**145**	**Homsap**	**16-G1v76**	**CH2**<br>**C27**	CH2<br>D27>C (265)	D265C	IGHG1 CH2<br>23-31 (261-269)<br>CVVVDVSHE ><br>CVVVCVSHE	**Conjugation site-specific**.<br>Site-specific drug attachment engineered cysteine.		4<br>*opelkibart 12810 (129,91)...*
**146**	**Homsap**	**9-G1v77**	**CH3**<br>**A115**	CH3<br>H115>A (435)	H435A	IGHG1 CH3<br>FG 105-117 (426-437)<br>SVMHEA.LHNHYT ><br>SVMHEA.LHNAYT	**Half-life decrease.**<br>Reduces FCGRT binding at pH 6.0.<br>Faster *in vivo* clearance [157].		2<br>*opelkibart 12810 (129,91), opelkibart elmanitin 12811 (129,91).*
**147**	**Homsap**	**9-G1v77-1**	**CH2**<br>**A15.2,**<br>**A93**<br>**CH3**<br>**A115**	CH2<br>I15.2>A (253),<br>H93>A (310),<br>CH3<br>H115>A (435)	I253A,<br>H310A,<br><br>H435A	IGHG1 CH2<br>13-18 (249-256)<br>DTLMISRT ><br>DTLMASRT<br>89-96 (306-313)<br>LTVLHQDW ><br>LTVLAQDW<br>IGHG1 CH3<br>FG 105-117 (426-437)<br>SVMHEA.LHNHYT ><br>SVMHEA.LHNAYT	**Half-life decrease.**<br>Reduces FCGRT binding at pH 6.0.<br>Faster *in vivo* clearance		1<br>*faricimab 10563 (118,80).*
**148**	**Homsap**	**9-G1v77-2**	**CH2**<br>**K17**	CH2<br>R17>K (255)	R255K	IGHG1 CH2<br>13-18 (249-256)<br>DTLMISRT ><br>DTLMISKT	**Half-life decrease.**<br>Reduces FCGRT binding at pH 6.0.<br>Faster *in vivo* clearance		1<br>*tuvonralimab 11841 (125,87).*

**149**	**Homsap**	**9-G1v78**	**CH3**<br>**L107**<br>**A114**	CH3<br>**M**107>**L** (428),<br>**N**114>**A** (434)	M428L,<br>N434A	IGHG1 CH3<br>FG 105-117 (426-437)<br>SVMHEA.LHNHYT ><br>SVLHEA.LHAHYT	**Half-life increase.**<br>Enhances FCGRT binding at pH 6.0.	**6**<br>*crovalimab 10882 (119,81)...*
**150**	**Homsap**	**9-G1v78-1**	**CH3**<br>**L107**<br>**A114**<br>**T116**	CH3<br>**M**107>**L** (428),<br>**N**114>**A** (434)<br>**Y**116>**T** (436)	M428L,<br>N434A,<br>Y436T	IGHG1 CH3<br>FG 105-117 (426-437)<br>SVMHEA.LHNHYT ><br>SVLHEA.LHAHTT	**Half-life increase.**<br>Enhances FCGRT binding at pH 6.0 [158], [159].	**1**<br>*emugrobart 13238 (131,93).*
**151**	**Homsap**	**9-G1v78-2**	**CH3**<br>**A114**	CH3<br>**N**114>**A** (434)	N434A	IGHG1 CH3<br>FG 105-117 (426-437)<br>SVMHEA.LHNHYT ><br>SVMHEA.LHAHYT	**Half-life increase.**<br>Enhances FCGRT binding at pH 6.0 [159].	0
**152**	**Homsap**	**9-G1v78-3**	**CH3**<br>**K115**	CH3<br>**H**115>**K** (435)	H435K	IGHG1 CH3<br>FG 105-117 (426-437)<br>SVMHEA.LHNHYT ><br>SVMHEA.LHNKYT<br>FG 105-117 (426-437)<br>SVMHEA.LHNHYT ><br>SVMHEA.LHNKYT	**Half-life increase.**<br>Enhances FCGRT binding at pH 6.0	**1**<br>*bapineuzumab 8624 (93,55).*
153	**Homsap**	**9-G1v78-4**	**CH3**<br>**C112,**<br>**S113,**<br>**Y114,**<br>**L116,**<br>**C117**	CH3<br>**L**112>**C** (432),<br>**H**113>**S** (433),<br>**N**114>**Y** (434),<br>**Y**116>**L** (443),<br>**T**117>**C** (444)<br>N3Y	L432C,<br>H433S,<br>N434Y,<br>Y436L,<br>T437C	IGHG1 CH3<br>FG 105-117 (426-437)<br>SVMHEA.LHNHYT ><br>SVMHEA.CSYHLC	**Half-life increase.**<br>Enhances FCGRT binding at pH 6.0	**1**<br>*tepsababart 13902 (134,)*
**154**	**Homsap**	**1-G1v79**	**CH2**<br>**G27**<br>**Q110**<br>**S115**	CH2<br>**D**27>**G** (265),<br>**A**110>**Q** (327),<br>**A**115>**S** (330)	D265G,<br>A327Q,<br>A330S	IGHG1 CH2<br>23-31 (261-269)<br>CVVVDVSHE ><br>CVVVGVSHE<br>FG 105-117 (322-332)<br>KVSNKA..LPAPI ><br>KVSNKQ..LPSPI	**ADCC reduction**.<br>Decreases FcgammaR binding.	0<br>part of G1v50-79.

**155**	**Homsap**	**1-G1v80**	**CH2**<br>**G116**	CH2<br>**P**116>**G** (331)	P331G	IGHG1 CH2<br>FG 105-117 (322-332)<br>KVSNKA..LPAPI ><br>KVSNKA..LPAGI	**ADCC reduction**.<br>Decreases FcgammaR binding.		0<br>part of G1v66-80.
**156**	**Homsap**	**16-G1v81**	**CH2**<br>**C81**	CH2<br>**K**81>**C** (290)	K290C	IGHG1 CH2<br>77- 84.1 (286-294)<br>NAKTKPREE ><br>NAKTCPREE	**Conjugation site-specific**.<br>Site-specific drug attachment engineered cysteine.		with 16-KCv93 :<br>**2** *micvotabart pelidotin 13003 (130,92), micvotabart 13004 (130,92).*
**157**	**Homsap**	**18-G1v82-1**	**CH3**<br>**L85.1**	CH3<br>**F**85.1>**L** (405)	F405L	IGHG1 CH3<br>85.4-92 (402-413)<br>GSFFLYSKLTVD ><br>GSFLLYSKLTVD	Enhances the formation of stable bispecific IgG1 by control of half-IG exchange, with 18-G1v82-2 on the other chain [160].		6wvz<br>**6**<br>*amivantamab 11030 (121,83)...*
**158**	**Homsap**	**18-G1v82-2**	**CH3**<br>**R88**	CH3<br>**K**88>**R** (409)	K409R	IGHG1 CH3<br>85.4-92 (402-413)<br>GSFFLYSKLTVD ><br>GSFFLYSRLTVD	Enhances the formation of stable bispecific IgG1 by control of half-IG exchange, with 18-G1v82-1 on the other chain [160].		6wvz<br>**6**<br>*amivantamab 11030 (121,83)...*
**159**	**Homsap**	**18-G1v82-3**	**CH3**<br>**K85.1**	CH3<br>**F**85.1>**K** (405)	F405K	IGHG1 CH3<br>85.4-92 (402-413)<br>GSFFLYSKLTVD ><br>GSFKLTVD	Enhances the heterodimerization of bispecific IgG1 with 18-G1v82-4 on the other chain [161].		**3**<br>*anbenitamab 11611 (124,86)...*
**160**	**Homsap**	**18-G1v82-4**	**CH3**<br>**A88**	CH3<br>**K**88>**A** (409)	K409A	IGHG1 CH3<br>85.4-92 (402-413)<br>GSFFLYSKLTVD ><br>GSFFLYSALTVD	Enhances the heterodimerization of bispecific IgG1 with 18-G1v82-3 on the other chain [161].		**3**<br>*anbenitamab 11611 (124,86)...*

**161**	**Homsap**	**18-G1v82-5**	**CH3**<br>**D24,**<br>**S26**	L24>D (368)<br>K26>S (370)	L368D,<br>K370S	IGHG1 CH3<br>12-26 (356-370)<br>DELT…KNQVSLTCLVK ><br>DELT…KNQVSLTCDVS	Enhances the heterodimerization of bispecific IgG1 with 18-G1v82-6 on the other chain.<br>Associated with 14-G1v72-3 (lower pI chain of the heterodimer) [162].		9<br>*plamotamab 11020 (120,82)…*
**162**	**Homsap**	**18-G1v82-6**	**CH3**<br>**Q13,**<br>**K20**	E13>Q (357)<br>S20>K (364)	E357Q,<br>S364K	IGHG1 CH3<br>12-26 (356-370)<br>DELT…KNQVSLTCLVK ><br>DQLT…KNQVKLTCLVK	Enhances the heterodimerization of bispecific IgG1 with 18-G1v82-5 on the other chain [162].		9<br>*plamotamab 11020 (120,82)…*
**163**	**Homsap**	**18-G1v82-7**	**CH3**<br>**R84.2,**<br>**E88**	D84.2>R (399)<br>K88>E (409)	D399R,<br>K409E	IGHG1 CH3<br>83-88 (396-409)<br>PVLDSD..GSFFLYSK ><br>PVLRSD..GSFFLYSE	Control of the expression of two antibodies in a single cell.	AA changes on the two H-gamma1 chains of one of the two antibodies.	1<br>*tuvonralimab 11841 (125,87).*
**164**	**Homsap**	**18,10-G1v82-8**<br>*chimerisotype G1[8aaTRAC,7aaG3,1aa°]*	**CH3**<br>**A3,**<br>**E12,**<br>**M14,**<br>**K20,**<br>**V22,**<br>**T26,**<br>**S44,**<br>**Y79°,**<br>**M84,**<br>**S85.1,**<br>**V86,**<br>**W88,**<br>**N90,**<br>**I101,**<br>**R115,**<br>**F116**	CH3<br>Q3>A (347),<br>D12>E (356),<br>L14>M (358),<br>S20>K (364),<br>T22>V (366),<br>K26>T (370),<br>N44>S (384),<br>K79>Y (392),<br>V84>M (397),<br>F85.1>S (405),<br>Y86>V (407),<br>K88>W (409),<br>T90>N (411),<br>V101>I (422),<br>H115>R (435),<br>Y116>F (436).	Q347A,<br>D356E,<br>L358M,<br>S364K,<br>T366V,<br>K370T,<br>N384S,<br>K392Y,<br>V397M,<br>F405S,<br>Y407V,<br>K409W,<br>T411N,<br>V422I,<br>H435R,<br>Y436F.	IGHG1 CH3<br>1.4-11 (341-355)<br>GQPREPQVYTLPPSR ><br>GQPREPAVYTLPPSR<br>12-26 (356-370)<br>DELT…KNQVSLTCLVK ><br>EEMT…KNQVKLVCLVT<br>39-45.4 (379-389)<br>VEWESNGQPEN ><br>VEWESSGQPEN<br>79-90 (397-413)<br>KTTPPVLDSD..GSFFLYSKLT ><br>YTTPPMLDSD..GSFSLVSWLN<br>97-104 (418-425)<br>QQGNVFSC ><br>QQGNIFSC<br>FG 105-117 (426-437)<br>SVMHEA.LHNHYT >	Enhances the heterodimerization of bispecific IgG1 with 18,10-G1v82-9 on the other chain [163], [164].<br><br>Includes 10-G1v83 (CH3 R115, F116) which abrogates binding to Protein A [165].	The backbone is IGHG1*01 with eight aa from TRAC (A3, K20, V22, T26, S85.1, V86, W88, N90), seven aa from G3 (E12, M14, S44, M84, I101, R115, F116) and one aa of unspecified origin (Y79°). *CH3 E12 and M14 are isoallotypic aa present in IGHG1*03 and in G2, G3 and G4.*	0

						SVMHEA.LHNRFT			
**165**	**Homsap**	**18,10-G1v82-9** *chimerisotype G1[11aaTRBC,3aa°]*	**CH3** **E3,** **A5,** **F7,** **T20,** **V22,** **T26,** **D81,** **L84°,** **E84.2°,** **Q84.4,** **A85.1°,** **S86,** **R88,** **R90**	CH3 Q3>E (347), Y5>A (349) L7>F (351) S20>T (364), T22>V (366), K26>T (370), T81>D (394), V84>L (397), D84.2>E (399), D84.4>Q (401), F85.1>A (405), Y86>S (407), K88>R (409), T90>R (411)	Q347E, Y349A, L351F, S364T, T366V, K370T, T394D, V397L, D399E, D401Q, F405A, Y407S, K409R, T411R	IGHG1 CH3 1.4-11 (341-355) GQPREPQVYTLPPSR > GQPREPEVATFPPSR 12-26 (356-370) DELT…KNQVSLTCLVK > DELT…KNQVTLVCLVT 79-90 (397-413) KTTPPVLDSD..GSFFLYSKLT > YTDPPVLESQ..GSFALSSRLR	Enhances the heterodimerization of bispecific IgG1 with 18,10-G1v82-8 on the other chain [163], [164].	The backbone is IGHG1*01 with eleven aa from TRBC and three aa of unspecified origin (L84°, E84.2° and A85.1°).	0
**166**	**Homsap**	**10-G1v83**	**CH3** **R115,** **F116**	CH3 H115>R (435), Y116>F (436) RF	H435R, Y436F	IGHG1 CH3 FG 105-117 (426-437) SVMHEA.LHNHYT > SVMHEA.LHNRFT 118-125 (438-445) QKSLSLSP	Abrogates binding to Protein A with G1 CH3 P125 [165] (in G4v8, a mutation CH3 L125>P is required). CH3 F116 (instead or Y) to minimize potential immunogenicity, R coexists with F in G3 [147].		8 *obrixtamig 12889 (130,92)…*
**167**	**Homsap**	**10-G1v83-1**	**CH3** **R115**	CH3 H115>R (435)	H435R	IGHG1 CH3 FG 105-117 (426-437) SVMHEA.LHNHYT > SVMHEA.LHNRYT	Abrogates binding to Protein A (facilitates the removal of homodimers from bispecific heterodimers).		4 *duvortuxizumab 10506 (116,78)…*
**168**	**Homsap**	**17-G1v84**	**CH1** **C81,** **C84**	CH1 F81>C (170), V84>C (173)	F170C, V173C	IGHG1 CH1 77-84.4 (166-177) GVHTFPAVLQSS > GVHTCPACLQSS >	Promotes the heteropairing of 17-G1v84 CH1 with 17-KCv84 L-kappa of a G1-kappa antibody.	Antibody expressed with another antibody (without AA changes) in the same cell.	1 *tuvonralimab 11841 (125,87).*
**169**	**Homsap**	**17-G1v84-1**	**VH** **E44**	VH Q44>E	Q44E	VH 39-46 *MIWVRQAP* >	Enhances the heteropairing of 17-		1

			**CH1**<br>**K86**	CH1<br>S86>K (183)	S183K	*MIWVREAP*<br>IGHG1 CH1<br>85.4-89 (178-186)<br>GLYSLSSVV ><br>GLYSLKSVV	G1v84-1 VH-CH1 with 17-Kv84-1 L-kappa in bispecific G1-kappa (the other chain is 17-G1v84-2).		*linclatamig 13026 (130,92).*
**170**	**Homsap**	**17-G1v84-2**	**VH**<br>**K44**<br>**CH1**<br>**E86**	VH<br>Q44>K,<br>CH1<br>S86>E (183)	Q44K,<br><br>S183E	VH 39-46<br>*MIWVRQAP* ><br>*MIWVRKAP*<br>IGHG1 CH1<br>85.4-89 (178-186)<br>GLYSLSSVV ><br>GLYSLESVV	Enhances the heteropairing of 17-G1v84-2 VH-CH1 with 17-Kv84-2 L-kappa in bispecific G1-kappa (the other chain is 17-Kv84-2).		**1**<br>*linclatamig 13026 (130,92).*
**171**	**Homsap**	**7-G1v85**	**CH2**<br>**W1.2**<br>**N1.1**<br>**D30**<br>**K115**	**CH2**<br>L1.2>W (235),<br>G1.1>N (236),<br>H30>D (268),<br>A115>K (330),	L235W,<br>G236N,<br>H268D,<br>A330K,	IGHG1 CH2<br>1.6-3 (231-239)<br>APELLGGPS ><br>APELWNGPS<br>23-31 (261-269)<br>CVVVDVSHE ><br>CVVVDVSDE<br>FG 105-117 (322-332)<br>KVSNKA..LPAPI ><br>KVSNKA..LPKPI	**FcγRIIB increase binding**.<br>Increases FcγRIIB binding affinity. Inhibits B cell activation by downstream ITIM signaling [150], L235W not quoted in [159].		**1**<br>*atenastobart 12848 (131,93).*
**172**	**Homsap**	**7-G1v86**	**CH2**<br>**Y1.3**<br>**D2**<br>**I26**<br>**K115**	**CH2**<br>L1.3>Y (234),<br>P2>D (238),<br>V26>I (264),<br>A115>K (330)	L234Y,<br>P238D,<br>V264I,<br>A330K	IGHG1 CH2<br>1.6-3 (231-239)<br>APELLGGPS ><br>APEYLGGDS<br>23-31 (261-269)<br>CVVVDVSHE ><br>CVVIDVSHE<br>FG 105-117 (322-332)<br>KVSNKA..LPAPI ><br>KVSNKA..LPKPI	**FcγRIIB increase binding**.<br>Increases FcγRIIB binding affinity. Inhibits B cell activation by downstream ITIM signaling [150].	Includes P2>D (238) (G1v63-2) which decreases binding to activating FcγR and to C1q [150].	**1**<br>*emugrobart 13238 (131,93).*
**173**	**Homsap**	**10-G1v87**	**CH2**<br>**V14,**<br>**P90**	**CH2**<br>T14>V (250),<br>T90>P (307)	 T250V,<br>T307P	IGHG1 CH2<br>13-18 (249-256)<br>DTLMISRT >	Improves CH2 thermal stability [158], [159].		**1**<br>*emugrobart 13238 (131,93).*

						DVLMISRT<br>89-96 (306-313)<br>LTVLHQDW ><br>LPVLHQDW			
**174**	**Homsap**	**10-G1v88**	**CH2**<br>**R94,**<br><br>**CH3**<br>**R1.2**	**CH2**<br>Q94>R (311),<br><br>**CH3**<br>P1.2>R (343)	Q311R,<br><br>P343R	IGHG1 CH2<br>89-96 (306-313)<br>LTVLHQDW ><br>LTVLHRDW<br>IGHG1 CH3<br>1.4-3 (341-347)<br>GQPREPQ ><br>GQRREPQ	Increases pI for enhanced uptake of immune complexes [158].		2<br>*atenastobart 12848 (131,93), emugrobart 13238 (131,93).*
**175**	**Homsap**	**10-G1v88-1**	**CH2**<br>**R94,**<br><br>**CH3**<br>**K92**	**CH2**<br>Q94>R (311),<br><br>**CH3**<br>D92>K (413)	Q311R,<br><br>D413K	IGHG1 CH2<br>89-96 (306-313)<br>LTVLHQDW ><br>LTVLHRDW<br>IGHG1 CH3<br>89-96 (410-417)<br>LTVDKSRW ><br>LTVKKSRW	Increases pI for enhanced uptake of immune complexes [158], [159].		0
**176**	**Homsap**	**10-G1v89**	**CH3**<br>**R118,**<br>**E120**	**CH3**<br>Q118>R (438)<br>S440>E (440)	Q438R<br>S440E	IGHG1 CH3<br>118-125 (438-445)<br>QKSLSLSP ><br>RKELSLSP	Suppresses rheumatoid factor binding [158].		3<br>*crovalimab 10882 (119,81)...*
**177**	**Homsap**	**14-G1v90**	**CH3**<br>**E16**<br>**W88**	**CH3**<br>K16>E (360)<br>K88>W (409)<br>EW	K360E<br>K409W	IGHG1 CH3<br>12-26 (356-370)<br>DELTKNQVSLTCLVK ><br>DELTENQVSLTCLVK<br>IGHG1 CH3<br>85.4-92 (402-413)<br>GSFFLYSKLTVD ><br>GSFFLYSWLTVD	Enhances with 14-G1v91 the heterodimerization H-H of bispecific G1 antibodies [166].		3<br>*ingitamig 12958 (131,93)..*
**178**	**Homsap**	**14-G1v91**	**CH3**<br>**R3**<br>**V84 .2**<br>**T85.1**	**CH3**<br>Q3>R (347)<br>D84.2>V (399),<br>F85.1>T (405)	Q347R,<br>D399V,<br>F405T	IGHG1 CH3<br>1.4-11 (341-355)<br>GQPREPQVYTLPPSR ><br>GQPREPRVYTLPPSR	Enhances with 14-G1v90 the heterodimerization H-H of bispecific G1		3<br>*ingitamig 12958 (131,93)...*

				RVT		84.1-92 (398-413)<br>LDSD..GSFFLYSKLTVD ><br>LVSD..GSFTLYSKLTVD	antibodies [166].		
**179**	**Homsap**	**17-G1v92-1**	**VH**<br>**E44**<br>**CH1**<br>**E77**	**VH**<br>Q44>E,<br>**CH1**<br>G77>E (166)	Q44E,<br><br><br>G166E	VH 39-46<br>*MHWVRQAP* ><br>*MHWVREAP*<br>CH1 45-45.3,77-80 (162-169)<br>ALTS....GVHT ><br>ALTS....EVHT	Enhances the H-L pairing of 17-G1v92-1 VH-CH1 with 17-Kv92-1 L-kappa.		1<br>*spevatamig 13260 (131,93).*
**180**	**Homsap**	**17-G1v92-2**	**VH**<br>**K44**<br>**CH1**<br>**K77**	**VH**<br>Q44>K,<br>**CH1**<br>G77>K (166)	Q44K,<br><br><br>G166K	VH 39-46<br>*MHWVRQAP* ><br>*MHWVRKAP*<br>CH1 45-45.3,77-80 (162-169)<br>ALTS....GVHT ><br>ALTS....KVHT	Enhances the H-L pairing of 17-G1v92-2 VH-CH1 with 17-Kv92-2 L-kappa.		1<br>*spevatamig 13260 (131,93).*
**181**	**Homsap**	**17-G1v93**	**CH1**<br>**C12**	**CH1**<br>K12>C (133)	K133C	IGHG1 CH1<br>1.4-15 (118-136)<br>ASTKGPSVFPLAPSSKSTS ><br>ASTKGPSVFPLAPSSCSTS	Promotes the H-L pairing of 17-G1v93 (C12) CH1 with 17-KCv37 (C121, S126) C-kappa.		1<br>*spevatamig 13260 (131,93).*
**182**	**Homsap**	**14-G1v94**	**CH1**<br>**C94**	CH1<br>S94>C (191)	S191C	IGHG1 CH1 85.4-96 (178-193)<br>GLYSLSSVVTVPSSSL ><br>GLYSLSSVVTVPSCSL	H-H bridge C94-C94'' restricts the flexibility between the two Fab arms.		1<br>*clesitamig 13237 (131,93).*
**183**	**Homsap**	**14-G1v95**	**CH1**<br>**E16,**<br>**D107,**<br>**T120,**<br>**CH2**<br>**Q30,**<br>**Q38,**<br>**CH3**<br>**Q11,**<br>**E98**	**CH1**<br>G16>E (137)<br>N107>D (203)<br>R120>T (214)<br>**CH2**<br>H30>Q (268)<br>K38>Q (274)<br>**CH3**<br>R11>Q (355)<br>Q98>E (419)	G137E,<br>N203D, R214T,<br>H268Q,<br>K274Q,<br>R355Q, Q419E	IGHG1 CH1 16-26 (137-147)<br>GGTAALGCLVK ><br>EGTAALGCLVK<br>104-121 (200-215)<br>CNVNHKPSNTKVDKRV ><br>CNVDHKPSNTKVDKTV ><br>CH2 27-41 (265-277)<br>DVSHEDPEVKFNW ><br>DVSQEDPEVQFNW<br>CH3 7-15 (351-359)<br>LPPSREEMT >	Enhances, with the other G1 chain (without AA changes at these 7 positions), the heterodimerization H-H, via electrostatic steering interactions, of bispecific G1 antibodies.		2<br>*velinotamig 13145 (131,93),*<br>*tepilukimig 13604 (133,95).*

						LPPSQEEMT<br>95-104 (416-425)<br>RWQQGNVFSC ><br>RWQEGNVFSC			
**184**	**Homsap**	**14-G1v96**	**hinge**<br>**R2**<br>**del6-8**<br><br>**V9**<br>**E10**	hinge<br>**P**2>**R** (217)<br>**DKT**6-8>**del** (221-223)<br>**H**9>**V** (224)<br>**T**10>**E** (225)	P217R<br>DKT221-223del<br>H224V<br>T225E	IGHG1 hinge<br>1-15 (216-230)<br>EPKSCDKTHTCPPCP ><br>ERKSC - - - VECPPCP	Limits the flexibility of the Fab arms and narrows the distance between effector cells and target cells in bispecific IgG1.		**1**<br>*velinotamig 13145 (131,93).*
**185**	**Homsap**	**14-G1v96-1**	**hinge**<br>**C7**<br>**del8**<br>**del10**	hinge<br>**K**7>**C**<br>**T**8>**del**<br>**T**10>**del**	K222C<br>T223del<br>T225del	IGHG1 hinge<br>1-15 (216-230)<br>EPKSCDKTHTCPPCP ><br>EPKSCDC - H - CPPCP	Increases the structural rigidity of the antibody.		**5**<br>*telisotuzumab vedotin 10365 (115,77)...*
**186**	**Homsap**	**6-G1v97**	**CH2**<br>**A1.3**<br>**A1.2,**<br>**A1,**<br>**S2,**<br>**A30,**<br>**S115,**<br>**S116**	CH2<br>**L**1.3>**A** (234),<br>**L**1.2>**A** (235),<br>**G**1>**A** (237),<br>**P**2>**S** (238),<br>**H**30>**A** (268),<br>**A**115>**S** (330),<br>**P**116>**S** (331)<br>G1sigma	L234A,<br>L235A,<br>G237A,<br>P238S,<br>H268A,<br>A330S,<br>P331S	IGHG1 CH2<br>1.6-3 (231-239)<br>APELLGGPS ><br>APEAAGASS<br>23-31 (261-269)<br>CVVVDVSHE ><br>CVVVDVSAE<br>FG 105-117 (322-332)<br>KVSNKG..LPAPI ><br>KVSNKA..LPSSI	**ADCC reduction**.<br>Reduces FcγR binding. Undetectable ADCC and ADCP [167].	**CDC reduction**.<br>Reduces C1q binding. Undetectable CDC [167].	**3D**<br>5w5l [167][186]<br>0
**187**	**Homsap**	**10,13-G1v98**	**CH3**<br>**R1**	CH3<br>**E**1>**R** (345)	E345R	IGHG1 CH3<br>1.4-3 (341-347)<br>GQPREPQ ><br>GQPRRPQ	Favors IgG1 hexamerization [116].		**1**<br>*tixestobart 13358 (132,94).*
**188**	**Homsap**	**10,13-G1v98-1**	**CH3**<br>**K1**	CH3<br>**E**1>**K** (345)	E345K	IGHG1 CH3<br>1.4-3 (341-347)<br>GQPREPQ ><br>GQPRKPQ	Favors IgG1 hexamerization by increased intermolecular Fc-Fc interactions after antigen binding on the cell surface [168].		0
**189**	**Homsap**	**14-G1v99-1**	**hinge**<br>**E6**<br>**CH3**	hinge<br>**D**6>**E**<br>CH3	D221E	IGHG1 hinge<br>1-15 (216-230)<br>EPKSCDKTHTCPPCP >	Enhances with 14-G1v99-2 the heterodimerization of		**2**<br>*ompekimig 13515 (132,94), tilrekimig*

			**E24**	L24>E	L368E	EPKSCEKTHTCPPCP<br>CH3 20-26 (364-370)<br>SLTCLVK ><br>SLTCEVK	bispecific antibodies.		*13516 (132,94).*
**190**	**Homsap**	**14-G1v99-2**	**hinge**<br>**R6**<br>**CH3**<br>**R88**	hinge<br>D6>R<br>CH3<br>K88>R	D221R<br><br>K409R	IGHG1 hinge<br>1-15 (216-230)<br>EPKSCDKTHTCPPCP ><br>EPKSCRKTHTCPPCP<br>CH3 85.4-92 (402-413)<br>GSFFLYSKLTVD ><br>GSFFLYSRLTVD	Enhances with 14-G1v99-1 the heterodimerization of bispecific antibodies.		**2**<br>*ompekimig 13515 (132,94), tilrekimig 13516 (132,94).*
**191**	**Homsap**	**14-G1v99-3**	**hinge**<br>**E6,**<br>**E13,**<br>**CH3**<br>**E24**	hinge<br>D6>E (221),<br>P13>E (228),<br>CH3<br>L24>E (368)	D221E,<br>P228E,<br><br>L368E	IGHG1 hinge<br>1-15 (216-230)<br>EPKSCDKTHTCPPCP ><br>EPKSCEKTHTCPECP<br>CH3 20-26 (364-370)<br>SLTCLVK ><br>SLTCEVK	Enhances with 14-G1v99-4 the heterodimerization of bispecific antibodies [169].		0
**192**	**Homsap**	**14-G1v99-4**	**hinge**<br>**R6,**<br>**R13,**<br>**CH3**<br>**R88**	hinge<br>D6>R,<br>P13>R,<br>CH3<br>K88>R	D221R,<br>P228R,<br><br>K409R	IGHG1 hinge<br>1-15 (216-230)<br>EPKSCDKTHTCPPCP ><br>EPKSCRKTHTCPRCP<br>CH3 85.4-92 (402-413)<br>GSFFLYSKLTVD ><br>GSFFLYSRLTVD	Enhances with 14-G1v99-3 the heterodimerizationof bispecific antibodies [169].		0
**193**	**Homsap**	**17-G1v100-1**	**CH1**<br>**E16**	CH1<br>G16>E	G137E	IGHG1 CH1 16-26 (137-147)<br>GGTAALGCLVK ><br>EGTAALGCLVK	Enhances with 17-KCv100-1 (N87) the heteropairing of G1 CH1 with C-KAPPA in Fab		**1**<br>*velaprumig 13342 (132,94).*
**194**	**Homsap**	**1-G1v101**	**CH2**<br>**R31**	CH2<br>E31>R	E269R	IGHG1 CH2<br>23-31 (261-269)<br>CVVVDVSHE ><br>CVVVDVSHR	**ADCC reduction**.<br>Reduces FcγR binding.	.	0
**195**	**Homsap**	**1,5-G1v101-20**	**CH2**<br>**R31,**<br>**A105**	E31>R,<br>K105>A	E269R,<br>K322A	IGHG1 CH2<br>23-31 (261-269)<br>CVVVDVSHE ><br>CVVVDVSHR	**ADCC reduction**.<br>Reduces FcγR binding.<br>(1-G1v101)	**CDC reduction**.<br>Reduces C1q binding.<br>(5-G1v20)	Combines 1-G1v101 (CH2 R1) and 5-G1v20 (CH2 A105).

						FG 105-117 (322-332)<br>KVSNKA..LPAPI ><br>AVSNKA..LPAPI			1<br>*frexalimab*<br>*11963 (126,88)*
196	Homsap	2-G1v102	**CH3**<br>**M81**	CH3<br>**T**81>**M**	T394M	IGHG1 CH3<br>77-84 (390-397)<br>NYKTTPPV ><br>NYKTMPPV	**ADCC enhancement**.<br>Increases FcγIIIA binding.		1<br>*ensituximab*<br>*9300 (103,65).*
197	Homsap	2-G1v103	**CH2**<br>**I11,**<br>**Q124**	CH2<br>**P**11>**I**,<br>**A**124>**Q**	P247I,<br>A339Q	IGHG1 CH2<br>4-12 (240-248)<br>VFLFPPKPK ><br>VFLFPPKIK<br>118-125 (333-340)<br>EKTISKAK ><br>EKTISKQK	**ADCC enhancement**.<br>Increases FcγIIIA binding. 20-fold increase in binding to both major alleles of FcγIIIA.		1<br>*ocaratuzumab*<br>*9590 (107,69).*
198	Homsap	3-G1v104	**CH2**<br>**A85.3**	CH2<br>**T**85.3>**A**	T299A	IGHG1 CH2<br>83-88 (292-305)<br>REEQYN..STYRVVSV ><br>REEQYN..SAYRVVSV	**ADCC and ADCP enhancement** in aglycosylated antibodies. Increases FcγRIIa binding and FcγRIIIa moderate binding.	Absence of N-glycosylation owing to CH2 A85.3 [170].	1<br>*opicinumab*<br>*10090 (113,75).*
199	Homsap	6-G1v104-1	**CH2**<br>**N85.4,**<br>**A85.3,**<br>**S85.2**	CH2<br>**S**85.4>**N**,<br>**T**85.3>**A**,<br>**Y**85.2>**S**<br>NNAS	S298N,<br>T299A,<br>Y300S	IGHG1 CH2<br>83-88 (292-305)<br>REEQYN..STYRVVSV ><br>REEQYN..NASRVVSV	**Eliminates effector function** owing to alternate engineered glycosylation site (N298 instead of N297) [171]		**3D**<br>6x3i<br>0
700	Homsap	7-G1v105	**CH3**<br>**P85.3**	CH3<br>**S**85.3>**P**	S403P	IGHG1 CH3<br>85.4-92 (402-413)<br>GSFFLYSKLTVD ><br>GPFFLYSKLTVD	**FcγRIIB increase binding**.<br>Increases FcγRIIB binding affinity. Inhibits B cell activation by downstream ITIM signaling.		1<br>*dalantercept* (FPIA)<br>*9427 (105,67).*
701	Homsap	7-G1v106	**CH2**<br>**E37**	CH2<br>**V**37>**E**	V273E	CH2 34-41 (270-277)<br>DPEVKFNW ><br>DPEEKFNW	**FcγRIIB increase binding.**	Increases FcγRIIA binding affinity.	1<br>*giloralimab*<br>*11241 (122,84).*

							Increases FcγRIIB binding. Inhibits by downstream ITIM signaling in B cells.		
**702**	**Homsap**	**4-G1v107**<br>*chimerisotype G1[10aaG3]*	**CH2**<br>**Q38,**<br>**K40,**<br>**F85.2,**<br>**T124,**<br>**CH3**<br>**E12,**<br>**M14,**<br>**S44,**<br>**N79,**<br>**M84,**<br>**I101.**	CH2<br>**K**38>**Q** (274),<br>**N**40>**K** (276),<br>**Y**85.2>**F** (300),<br>**A**124>**T** (339),<br><br>**D**12>**E** (356),<br>**L**14>**M** (358),<br>**N**44>**S** (384),<br>**K**79>**N** (392),<br>**V**84>**M** (397),<br>**V**101>**I** (422).<br>G1G3 113F<br>(3)	K274Q,<br>N276K,<br>Y300F,<br>A339T,<br><br>D356E,<br>L358M,<br>N384S,<br>K392N,<br>V397M,<br>V422I.	IGHG1 CH2<br>34-41 (270-277)<br>DPEVKFNW ><br>DPEVQFKW<br>83-88 (292-305)<br>REEQYN..STYRVVSV ><br>REEQYN..STFRVVSV<br>118-125 (333-340)<br>EKTISKAK ><br>EKTISKTK<br>IGHG1 CH3<br>39-45.4 (379-389)<br>VEWESNGQPEN ><br>VEWESSGQPEN<br>77-84 (390-397)10-G1<br>NYKTTPPV ><br>NYNTTPPM<br>97-104 (418-425)<br>QQGNVFSC ><br>QQGNIFSC	**CDC enhancement**.<br>Increases C1q binding [172].	Facilitates binding to Protein A<br><br>CH3 102-125 (423-445) are from G1 with H115 (435) and Y116 (436) to keep binding to Protein A [172].	IGHG1*01 backbone with ten G3 aa, four in CH2 (Q38, K40, F85.2, T124) and six in CH3 (E12, M14, S44, N79, M84, I101).<br>*CH3 E12 and M14 are isoallotypic aa present in IGHG1*03 and in G2, G3 and G4.*<br>0
**703**	**Homsap**	**5-G1v108**	**CH2**<br>**V115**	CH2<br>**A**115>**V** (330)	A330V	IGHG1 CH2<br>FG 105-117 (322-332)<br>KVSNKA..LPAPI ><br>KVSNKA..LPVPI		**CDC reduction**.<br>Reduces C1q binding.	4<br>*torapsel* (CPCA)<br>*8337 (91,53)…*
**704**	**Homsap**	**1-G1v109**	**CH2**<br>**S114**	CH2<br>**P**114>**S** (329)	P329S	IGHG1 CH2<br>FG 105-117 (322-332)<br>KVSNKA..LPAPI ><br>KVSNKA..LSAPI	**ADCC reduction**.<br>Reduces FcγR binding.		0
**705**	**Homsap**	**10-G1v110**	**CHS**<br>**delK2**	CHS<br>**K**2>del (447)	K447del	IGHG1 CHS<br>1-2 (446,447)<br>GK ><br>G -	Homogeneity of the IGHG1 C-terminal end.		nd

**706**	**Homsap**	**10-G1v111**	**CHS**<br>**delG1,**<br>**delK2**	CHS<br>G1>del (446),<br>K2>del (447)	G446del,<br>K447 del	IGHG1 CHS<br>1-2 (446,447)<br>GK ><br>- -	Homogeneity of the IGHG1 C-terminal end.		nd
**707**	**Homsap**	**6-G1v112**<br>*chimerisotype G1[12aaG4]*	**CH2**<br>**Q30,**<br>**Q38,**<br>**F84.3,**<br>**G110,**<br>**S115,**<br>**S116,**<br>**CH3**<br>**Q11,**<br>**E12,**<br>**M14,**<br>**R88,**<br>**E98,**<br>**L125.**	CH2<br>H30>Q (268),<br>K38>Q (274),<br>Y84.3>F (296),<br>A110>G (327),<br>A115>S (330),<br>P116>S (331)<br>CH3<br>R11>Q (355)<br>D12>E (356),<br>L14>M (358),<br>K88>R (409),<br>Q98>E (419),<br>P125>L (445).	H268Q,<br>K274Q,<br>Y296F,<br>A327G,<br>A330S,<br>P331S,<br><br>R355Q,<br>D356E,<br>L358M,<br>K409R, Q419E,<br>P445L.	IGHG1 CH2<br>23-38 (261-274)<br>CVVVDVSHEDPEVK ><br>CVVVDVSQEDPEVQ<br>83-88 (292-305)<br>REEQYN..STYRVVSV ><br>REEQFN..STYRVVSV<br>FG 105-117 (322-332)<br>KVSNKA..LPAPI ><br>KVSNKG..LPSSI<br>IGHG1 CH3<br>7-15 (351-359)<br>LPPSRDELT ><br>LPPSQEEMT<br>83-89 (396-410)<br>PVLDSD..GSFFLYSKL<br>PVLDSD..GSFFLYSRL<br>97-104 (418-425)<br>QQGNVFSC<br>QEGNVFSC<br>118-125 (438-445)<br>QKSLSLSP ><br>QKSLSLSL	**ADCC reduction.**<br>Reduces FcγR binding.	**CDC reduction**.<br>Reduces C1q binding.	**1** *cabotamig 12632 (129,91).*<br>IGHG1*01 backbone with twelve G4 aa, six in the CH2 (Q30, Q38, F84.3, G110, S115, S116) and six in the CH3 (Q11, E12, M14, R88, E98, L125), making the CH2 starting from Q30 and the CH3 identical to those of IGHG4*01.<br>*CH3 E12 and M14 are isoallotypic aa present in IGHG1*03 and in G2, G3 and G4.*
**708**	**Homsap**	**18-G1v113**<br>*chimerisotype G1_AG SEED CH3[26aaA1]*	**CH3**<br>**F1.1,**<br>**R1,**<br>**E3,**<br>**H5,**<br>**L6,**<br>**E12,**<br>**M14,**<br>**A25,**	CH3<br>R1.1>F (344),<br>E1>R (345),<br>Q3>E (347),<br>Y5>H (349)<br>T6>L (350),<br>D12>E (356),<br>L14>M (358),<br>V25>A (369),	R344F,<br>E345R,<br>Q347E,<br>Y349H,<br>T350L,<br>D356E,<br>L358M,<br>V369A,	IGHG1 CH3<br>1.4-15 (341-359)<br>GQPREPQVYTLPPSRDELT ><br>GQPFRPEVHLLPPSREEMT<br><br>23-38 (367-378)	Promotes with 18-G1v114, by CH3 complementary assymetric strand exchange engineered domain (SEED) composed of alternative IGHA1 and IGHG1 motifs, the		0

			**R26,**<br>**K35,**<br>**S83,**<br>**R84,**<br>**Q84.1,**<br>**E84.2,**<br>**P84 .3,**<br>**S84.4,**<br>**Q84.5,**<br>**G84.6,**<br>**T85.5,**<br>**T85.4,**<br>**T85.3,**<br>**A85.1,**<br>**V85,**<br>**T86,**<br>**T120,**<br>**I121.**	K26>R (370),<br>S35>K (375),<br>P83>S (396),<br>V84>R (397),<br>L84.1>Q (398),<br>D84.2>E (399),<br>S84.3>P (400),<br>D84.4>S (401),<br>ga84.5>Q (401a),<br>ga84.6>G (401b),<br>ga85.5 >T (402a),<br>G85.4 >T (402),<br>S85.3>T (403),<br>F85.1>A (405),<br>L85>V (406),<br>Y86>T (407),<br>S120>T (440),<br>L121>I (441).<br>AG SEED	K370R,<br>S375K,<br>P396S,<br>V397R,<br>L398Q,<br>D399E,<br>S400P,<br>D401S,<br>gap401aQ,<br>gap401bG,<br>gap402aT,<br>G402T,<br>S403T,<br>F405A,<br>L406V,<br>Y407T,<br>S440T,<br>L441I.	CLVKGFYP..SDIA ><br>CLARGFYP..KDIA<br>83-87 (396-408)<br>PVLDSD . . .GSFFLYS ><br>SRQEPSQGTTTFAVTS<br><br>118-125 (438-445)<br>QKSLSLSP ><br>QKTISLSP	heterodimerization H-H of bispecific G1 antibodies [173].		
**709**	**Homsap**	**18-G1v114**<br>*chimerisotype G1_GA SEED CH3[34aaA1]*	**CH3**<br>**P10,**<br>**S11,**<br>**E12,**<br>**A15,**<br>**L15.1,**<br>**N16,**<br>**E17,**<br>**L18,**<br>**T20,**<br>**L42,**<br>**Q43,**<br>**G44,**<br>**S45,**<br>**E45.2,**<br>**L45.3,**<br>**P45.4,**	CH3<br>S10>P (354),<br>R11>S (355),<br>D12>E (356),<br>T15>A (359),<br>gap15.1>L (359a)<br>K16>N (360),<br>N17>E (361),<br>Q18>L (362),<br>S20>T (364),<br>E42>L (382),<br>S43>Q (383),<br>N44>G (384),<br>G45>S (385),<br>P45.2>E (387),<br>E45.3>L (388),<br>N45.4>P (389),	S354P,<br>R355S,<br>D356E,<br>T359A,<br>gap359aL,<br>K360N,<br>N361E,<br>Q362L,<br>S364T,<br>E382L,<br>S383Q,<br>N384G,<br>G385S,<br>P387E,<br>E388L,<br>N389P,	IGHG1 CH3<br>7-23 (351-367)<br>LPPSRDELT. . . KNQVSLTC ><br>LPPPSEELAL . . NELVTLTC<br><br>39-45.7,77-82 (379-395)<br>VEWESNGQPEN. . .NYKTTP ><br>VEWLQGSQELPRE.KYLTWA	Promotes with 18-G1v113, by CH3 complementary assymetric strand exchange engineered domain (SEED) composed of alternative IGHG1 and IGHA1 motifs, the heterodimerization H-H of bispecific G1 antibodies [173].		0

			**R45.5,**<br>**E45.6,**<br>**K77,**<br>**L79,**<br>**W81,**<br>**A82,**<br>**I88,**<br>**R90,**<br>**A92,**<br>**A93,**<br>**E94,**<br>**D95,**<br>**K97,**<br>**K98,**<br>**D100,**<br>**T101,**<br>**D122,**<br>**R123.**	**ga**45.5>**R** (389a)<br>**ga**45.6>**E** (389b)<br>**N**77>**K** (390),<br>**K**79>**L** (392),<br>**T**81>**W** (394),<br>**P**82>**A** (395),<br>**K**88>**I** (409),<br>**T**90>**R** (411),<br>**D**92>**A** (413),<br>**K**93>**A** (414),<br>**S**94>**E** (415),<br>**R**95>**D** (416),<br>**Q**97>**K** (418),<br>**Q**98>**K** (419),<br>**N**100>**D** (421),<br>**V**101>**T** (422),<br>**S**122>**D** (442),<br>**L**123>**R** (443).<br>GA SEED	gap389aR,<br>gap389bE,<br>N390K,<br>K392L,<br>T394W,<br>P395A,<br>K409I,<br>T411R,<br>D413A,<br>K414A,<br>S415E,<br>R416D,<br>Q418K,<br>Q419K,<br>N421D,<br>V422T,<br>S442D,<br>L443R.	88-104 (409-425)<br>KLTVDKSRW..QQGNVFSC ><br>ILRVAAEDW..KKGDTFSC<br><br>118-125 (438-445)<br>QKSLSLSP ><br>QKSLDRSP			
**710**	**Homsap**	**3,4-G1A1v1**<br>*chimerisotype G1-CH1hCH2[10aaA1] CH3[70aaA1].*<br><br>CH2 G20.1 and CH3 S1.5 are two experimental insertions.	**IGHG1**<br>**CH2**<br>**A10,**<br>**L11,**<br>**E12,**<br>**L14,**<br>**L15.1,**<br>**del15.2**<br>**G16,**<br>**S17,**<br>**E18,**<br>**A19,**<br>**N20**<br>**G20.1.**<br>**CH3**<br>**S1.5**<br>**N1.3,**	 CH2<br>**K**10>**A** (246)<br>**P**11>**L** (247)<br>**K**12>**E** (248)<br>**T**14>**L** (250)<br>**M**15.1>**L** (252)<br>**I**15.2 (253)> **-**<br>**S**16>**G**(254)<br>**R**17>**S** (255)<br>**T**18>**E** (256)<br>**P**19>**A** (257)<br>**E**20>**N** (258)<br>ins**G** (258a)<br>CH3<br>ins**S** (340a)<br>**Q**1.3>**N** (342)	 <br>K246A,<br>P247L,<br>K248E,<br>T250L,<br>M252L,<br>I253del,<br>S254G,<br>R255S,<br>T256E,<br>P257A,<br>E258N.<br>ins258aG<br><br>ins340aS<br>Q342N,	IGHG1 CH2 (10aa A1)<br>9-23 (245-261) (10A1,5com,1i,C)<br>PKPKDTLMI.SRTPE .VTC ><br>PALEDLL L. .GSEANGVTC<br><br>CH3 (70aa A1 (green), 37aa common to G1 (black), 4 conserved C23, C104 (pink), W41, L89 (blue)).	**ADCC enhancement.** Displays high affinity to FcαRI on neutrophils. Retains binding to FcγRI and FcγRIIA<br>**ADCP enhancement.** High affinity to FcαRI on macrophages. [174].	**CDC enhancement.** Binds C1q with high affinity. Exhibits a 3-fold lower dissociation constant with C1q as compared to IgG1, which translates to improved CDC activity [174].	trastuzumab-IgGA [174]<br><br>The backbone is IGHG1*01 for the CH1-hinge-CH2 with ten IGHA1 aa in the CH2 and sixty-eight specific IGHA1 aa in the CH3.

			**T1.2,**	P1.2>T (343)	P343T,	1.5-15.1 (340a-359a) (1i,12A1,8com)			
			**F1.1,**	R1.1>F (344)	R344F,	.GQPREPQVYTLPPSRDELT. >			
			**R1,**	E1>R (345)	E345R,	SGNTFRPEVHLLPPSEELAL			
			**E3**	Q3>E (347)	Q347E,				
			**H5,**	Y5>H (349)	Y349H,				
			**L6,**	T6>L (350)	T350L,				
			**P10,**	S10>P (354)	S354P,				
			**S11,**	R11>S (355)	R355S,				
			**E12,**	D12>E (356)	D356E,				
			**A15,**	T15>A (359)	T359A,	16-38 (360-378) (10A1,10com,C)			
			**L15.1**,	ins15.1 L (359a)	ins359aL	KNQVSLTCLVKGFYP..SDIA >			
			**N16,**	K16>N (360)	K360N,	NELVTLTCLARGFSP..KDVL			
			**E17,**	N17>E (361)	N361E,				
			**L18,**	Q18>L (362)	Q362L,				
			**T20,**	S20>T (364)	S364T,				
			**A25,**	V25>A (369)	V369A,				
			**R26,**	K26>R (370)	K370R,				
			**S29,**	Y29>S (373)	Y373S,				
			**K35,**	S35>K (375)	S375K,	39-45.7,77-82 (379-395) (14A1,4com,W)			
			**V37,**	I37>V (377)	I377V,	VEWESNGQPEN. . . NYKTTP >			
			**L38**	A38>L(378)	A378L,	VRWLQGSQELPRE.KYLTWA			
			**R40,**	E40>R (380)	E380R,				
			**L42,**	E42>L (382)	E382L,				
			**Q43,**	S43>Q (383)	S383Q,				
			**G44,**	N44>G (384)	N384G,				
			**S45,**	G45>S (385)	G385S,				
			**E45.2,**	P45.2>E (387)	P387E,				
			**L45.3,**	E45.3>L (388)	E388L,				
			**P45.4,**	N45.4>P (389)	N389P,				
			**R45.5,**	ins45.5 R (389a)	ins389aR,				
			**E45.6,**	ins45.6 E (389b)	ins389bE,				
			**K77,**	N77>K (390)	N390K	83-87 (396-408) (14A1,2com)			
			**L79,**	K79>L (392)	K392L,	PVLDSD . . . GSFFLYS >			
			**W81,**	T81>W (394)	T394W,	SRQEPSQGTTTFAVTS			
			**A82**	P82>A (395)	P395A,				
			**S83,**	P83>S (396)	P396S,				

			**R84,**<br>**Q84.1,**<br>**E84.2,**<br>**P84.3,**<br>**S84.4,**<br>**Q84.5,**<br>**G84.6,**<br>**T85.5,**<br>**T85.4,**<br>**T85.3,**<br>**A85.1,**<br>**V85,**<br>**T86**<br>**I88,**<br>**R90,**<br>**A92,**<br>**A93,**<br>**E94,**<br>**D95,**<br>**K97,**<br>**K98,**<br>**D100,**<br>**T101**<br>**M105,**<br>**G107,**<br>**P113,**<br>**L114,**<br>**A115,**<br>**F116,**<br>**T120,**<br>**I121,**<br>**D122,**<br>**R123.**	V84>R (397)<br>L84.1>Q (398)<br>D84.2>E (399)<br>S84.3>P (400)<br>D84.4>S (401)<br>ins84.5 Q (401a)<br>ins84.6 G (401b)<br>ins85.5 T (402a)<br>G85.4>T (402)<br>S85.3>T (403)<br>F85.1>A (405)<br>L85>V (406)<br>Y86>T (407)<br>K88>I (409)<br>T90>R (411)<br>D92>A (413)<br>K93>A (414)<br>S94>E (415)<br>R95>D (416)<br>Q97>K (418)<br>Q98>K (419)<br>N100>D (421)<br>V101>T (422)<br>S105>M (426)<br>M107>G (428)<br>H113>P (433)<br>N114>L (434)<br>H115>A (435)<br>Y116>F (436)<br>S120>T (440)<br>L121>I (441)<br>S122>D (442)<br>L123>R (443)	V397R,<br>L398Q,<br>D399E,<br>S400P,<br>D401S,<br>ins401aQ,<br>ins401bG,<br>ins402aT,<br>G402T,<br>S403T,<br>F405A,<br>L406V,<br>Y407T,<br>K409I,<br>T411R,<br>D413A,<br>K414A,<br>S415E<br>R416D<br>Q418K,<br>Q419K,<br>N421D,<br>V422T,<br>S426M,<br>M428G,<br>H433P,<br>N434L,<br>H435A,<br>Y436F,<br>S440T,<br>L441I,<br>S442D,<br>L443R.	88-104 (409-425) (10A1,5com,L,C)<br>KLTVDKSRW..QQGNVFSC ><br>ILRVAAEDW..KKGDTFSC<br><br>105-123 (426-443) (10A1,8com)<br>SVMHEA.LHNHYTQKSLSLSP ><br>MVGHEA.LPLAFTQKTIDR			
**711**	**Homsap**	**10-G1v115**	**IGHG1**<br>**CH3**<br>**Y44,**	 CH3<br>N44>Y	 <br>N384Y,	IGHG1 CH3<br>39-45.4 (379-389)<br>VEWESNGQPEN >	Promotes the binding to the ***Homo sapiens*** **transferrin receptor**		1<br>*tividenofusp alfa*<br>*12197 (128,90)*

			T45.1,<br>E45.2,<br>W45.3,<br>A45.4,<br>T92,<br>E94,<br>E95,<br>F100.	Q45.1>T<br>P45.2>E<br>E45.3>W<br>N45.4>A<br>D92>T<br>S94>E<br>R95>E<br>N100>F	Q386T,<br>P387E,<br>E388W,<br>N389A<br>D413T,<br>S415E,<br>R416E,<br>N421F.	VEWESYGTEWA<br>92-104 (418-425)<br>DKSRW..QQGNVFSC ><br>TKEEW..QQGFVFSC	**type 1 (TfR1)** on blood-brain barrier (BBB) endothelial cells**.**<br>(Enzyme TransportVehicle™ (ETV)).		
**793**<br>°	**Homsap**	**G1 CH1 A121**	**CH1**<br>**A121**		V215A		no property or function linked to A121 (V215A).		Not in the engineered variant list.
**794**<br>°°	**Homsap**	**G1CH1_10-17_3giz**	**CH1**<br>**G10*,**<br>**S12*,**<br>**K13*,**<br>**S14*,**<br>**T15*,**<br>**S16*.**	CH1<br>S10>G (131)*,<br>K12>S (133)*,<br>S13>K (134)*,<br>T14>S (135)*,<br>S15>T (136)*,<br>G16>S (137)*.	S131G*,<br>K133S*,<br>S134K*,<br>T135S*,<br>S136T*,<br>G137S*.	IGHG1 CH1<br>9-18 (130-139)<br>PSSKSTS…GGT: correct sequence<br>PGSSKST…SGT: *wrong sequence	***Wrong sequence** of *ofalimumab* in 3giz_H (3D) due to the displacement of a G from position 17 to 10 (resulting in G17 deletion and G10 insertion).	**The Fab sequence** of *ofalimumab* 6y9a_H (3D) is correct.	Not in the engineered variant list.
**795***	**Homsap**	**2,7-G1v7,**<br>**10-G1v7-1**<br>*G1[8aaG2]*	**CH2**<br>**D3,**<br>**Q38,**<br>**F84.3,**<br>**F85.2,**<br>**V92,**<br>**E117,**<br>**T124,**<br>**CH3**<br>**E12,**<br>**M14,**<br>**M84.**	CH2<br>S3>D (239),<br>K38>Q (274),<br>Y84.3>F (296),<br>Y85.2>F (300),<br>L92>V (309),<br>I117>E (332),<br>A124>T (339),<br>CH3<br>D12>E (356),<br>L14>M (358),<br>V84>M (332)	S239D,<br>K274Q,<br>Y296F, Y300F,<br>L309V,<br>I332E, A339T,<br><br>D356E,<br>L358M,<br>V397M	IGHG1 CH2<br>1.6-3 (231-239)<br>APELLGGPS ><br>APELLGGPD<br>34-41 (270-277)<br>DPEVKFNW ><br>DPEVQFNW<br>83-93 (292-310)<br>REEQYN..STYRVVSVLTVLH ><br>REEQFN..STFRVVSVLTVVH<br>FG 105-117 (322-332)<br>KVSNKA..LPAPI ><br>KVSNKA..LPAPE<br>G 118-125 (333-340)<br>EKTISKAK ><br>EKTISKTK<br>CH3 7-15 (351-359)	**ADCC enhancement**.<br>Increases FcγRIIIA binding [108].<br>Particularly the 'lower-affinity' FcγRIIIA F158 allele (2,7-G1v7, CH2 D3 and E117).	**FcγRIIB.**<br>Strong binding to inhibitory FcγRIIB [108].<br>(2,7G1v7, CH2 D3 and E117).	IGHG1*01 backbone with eight G2 aa, five in CH2 (Q38, F84.3, F85.2, V92, T124) and three in CH3 (E12, M14, M84).<br>*CH3 E12 and M14 are isoallotypic aa present in IGHG1*03 and in G2, G3 and G4.*<br>*(2) talacotuzumab 10508 (117,79), tafasitamab 10835 (119,81).*

						LPPSRDELT ><br>LPPSREEMT<br>77-84 (390-397)<br>NYKTTPPV ><br>NYKTTPPM			
**796***	**Homsap**	**14-G1v90,**<br>**14-G1v75**	**CH3**<br>**C5**<br>**E16**<br>**W88**	**CH3**<br>**Y**5>**C** (349)<br>**K16>E** (360)<br>**K88>W** (409)	Y349C,<br>K360E,<br>K409W	IGHG1 CH3<br>1.4-11 (341-355)<br>GQPREPQVYTLPPSR ><br>GQPREPQVCTLPPSR<br>12-26 (356-370)<br>DELTKNQVSLTCLVK ><br>DELTENQVSLTCLVK<br>85.4-92 (402-413)<br>GSFFLYSKLTVD ><br>GSFFLYSWLTVD	14-G1v90 (CH3 E16,W88) enhances with 14-G1v91 the heterodimerization H-H of bispecific G1 antibodies [166].	14-G1v75 (CH3 C5) promotes, with 14-G1v74 by a disulfide bridge, the heterodimerization H-H of bispecific G1 antibodies [156].	[175]<br>(3)<br>*ingitimab 12958 (131,93)…*
**797 ***	**Homsap**	**14-G1v91,**<br>**14-G1v74**	**CH3**<br>**R3,**<br>**C10,**<br>**V84 .2,**<br>**T85.1**	**CH3**<br>**Q**3>**R** (347)<br>**S**10>**C** (354)<br>**D**84.2>**V** (399),<br>**F**85.1>**T** (405)	Q347R,<br>S354C,<br>D399V,<br>F405T	IGHG1 CH3<br>1.4-11 (341-355)<br>GQPREPQVYTLPPSR ><br>GQPREPRVYTLPPCR<br>84.1-92 (398-413)<br>LDSD..GSFFLYSKLTVD ><br>LVSD..GSFTLYSKLTVD	14-G1v91 (CH3 R3, V84.2, T85.1) enhances with 14-G1v90 the heterodimerization H-H of bispecific G1 antibodies [166].	14-G1v74 (CH3 C10) promotes with 14-G1v75, by a disulfide bridge, the heterodimerization H-H of bispecific G1 antibodies [156].	[175]<br>(3)<br>*ingitimab 12958 (131,93)…*
**798 ***	**Homsap**	**6-G1v53, 9-G1v21**	**CH2**<br>**F1.3,**<br>**Q1.2,**<br>**Q105**<br>**Y15.1,**<br>**T16,**<br>**E18**	CH2<br>**L**1.3>**F** (234),<br>**L**1.2>**Q** (235),<br>**K**105> **Q** (322)<br>**M**15.1>**Y** (252),<br>**S**16>**T** (254),<br>**T**18>**E** (256)<br>FQQ-YTE	L234F,<br>L235Q,<br>K322Q,<br>M252Y,<br>S254T,<br>T256E	IGHG1 CH2<br>1.6-3 (231-239)<br>APELLGGPS ><br>APEFQGGPS<br>13-18 (249-256)<br>DTLMLSRT ><br>DTLYITRE<br>FG 105-117 (322-332)<br>KVSNKA..LPAPI ><br>QVSNKA..LPAPI	**ADCC reduction.**<br>Reduces FcγR binding (6-G1v53)<br>[143].	**CDC reduction.**<br>Reduces C1q binding (6-G1v53)<br>[143].	**Half-life increase.**<br>Enhances FCGRT binding at pH 6.0 (9-G1v21) [117].<br><br>0

**799** *	**Homsap**	**6-G1v63,** **15-G1v70**	**hinge** **S5,** **S11,** **S14,** **CH2** **S2**	hinge C5>S (220), C11>S (226), C14>S (229), CH2 P2>S (238)	C220S, C226S, C229S, P238S	IGHG1 hinge 1-15 (216-230) EPKSCDKTHTCPPCP > EPKSSDKTHTSPPSP IGHG1 CH2 1.6-3 (231-239) APELLGGPS > APELLGGSS	**ADCC reduction**. Reduces FcγR binding (6-G1v63).	**CDC reduction.** Reduces C1q binding (6-G1v63).	4 Comprises 6-G1v63 (CH2 S2) and 15-G1v70 [a combination of 15-G1v37 (h S5, no H-L), 15-G1v61 (h S11, no inter H-H h11) and 15-G1v62 (h S14, no inter H-H h14)].
**201**	**Homsap**	**2-G2v1**	**CH2** **P1.4** **L1.3,** **L1.2,** **G1.1**	CH2 gap1.4>P (233), P1.3>L (234), V1.2>L (235), A1.1>G (236) (G1-like)	gap233P, P234L, V235L, A236G	IGHG2 CH2 1.6-3 (231-239) AP.PVAGPS > APPLLGGPS (G1-like)	**ADCC enhancement**. Confers FcγRI binding (WT does not show any binding capacity) [101].		0
**202**	**Homsap**	**6-G2v2**	**CH2** **Q30,** **L92,** **S115,** **S116**	CH2 H30>Q (268), V92>L (309), A115>S (330), P116>S (331) IgG2m4	H268Q, V309L, A330S, P331S	IGHG2 CH2 23-31 (261-269) CVVVDVSHE > CVVVDVSQE 89-96 (306-313) LTVVHQDW > LTVLHQDW FG 105-117 (322-332) KVSNKG..LPAPI > KVSNKG..LPSSI	**ADCC reduction**. Reduces FcγR binding [176].	**CDC reduction**. Reduces C1q binding [176].	0
**203**	**Homsap**	**6-G2v3**	**CH2** **A1.2,** **A1,** **S2,** **A30,** **L92,** **S115,** **S116**	CH2 V1.2>A (235), G1>A (237), P2>S (238), H30>A (268), V92>L (309), A115>S (330), P116>S (331) G2sigma	V235A, G237A, P238S, H268A, V309L, A330S, P331S	IGHG2 CH2 1.6-3 (231-239) AP.PVAGPS > AP.PAAASS 23-31 (261-269) CVVVDVSHE > CVVVDVSAE 89-96 (306-313) LTVVHQDW >	**ADCC reduction**. Reduces FcγR binding. Undetectable ADCC and ADCP [137].	**CDC reduction**. Reduces C1q binding. Undetectable CDC [137].	**3D** 4l4j [137] 2 *denrikibart 13177 (131,93), melrilimab 11351 (123,85).*

						LTVLHQDW<br>FG 105-117 (322-332)<br>KVSNKG..LPAPI ><br>KVSNKG..LPSSI			
**204**	**Homsap**	**6-G2v3-1**	**CH2**<br>**A1.2,**<br>**A1**	CH2<br>V1.2>A (235),<br>G1>A (237)	V235A,<br>G237A	IGHG2 CH2<br>1.6-3 (231-239)<br>AP.PVAGPS ><br>AP.PAAAPS	**ADCC reduction**.<br>Reduces FcγR binding.	**CDC reduction**.<br>Reduces C1q binding.	2 *visilizumab 8054 (84,46), quetmolimab 11040 (120,82).*
**205**	**Homsap**	**9-G2v4**	**CH2**<br>**Q14**	CH2<br>T14>Q (250)	T250Q	IGHG2 CH2<br>13-18 (249-256)<br>DTLMISRT ><br>DQLMISRT	**Half-life increase.**<br>Enhances FCGRT binding at pH 6.0 [177].		0
**206**	**Homsap**	**9-G2v5**	**CH3**<br>**L107**	CH3<br>M107>L (428)	M428L	IGHG2 CH3<br>FG 105-117 (426-437)<br>SVMHEA.LHNHYT ><br>SVLHEA.LHNHYT	**Half-life increase.**<br>Enhances FCGRT binding at pH 6.0 [177].		0
**207**	**Homsap**	**9-G2v6**	**CH2**<br>**Q14**<br><br>**CH3**<br>**L107**	CH2<br>T14>Q (250)<br><br>CH3<br>M107>L (428)	T250Q<br><br>M428L	IGHG2 CH2<br>13-18 (249-256)<br>DTLMISRT ><br>DQLMISRT<br>CH3<br>FG 105-117 (426-437)<br>SVMHEA.LHNHYT ><br>SVLHEA.LHNHYT	**Half-life increase.**<br>Enhances FCGRT binding at pH 6.0 [177].		0
**208**	**Homsap**	**10,6-G2v7**	**CH2**<br>**Y85.2,**<br>**L92,**<br>**A339**	**CH2**<br>F85.2>Y (300),<br>V92>L (309),<br>T339>A (339)	F300Y,<br>V309L,<br>T339A	IGHG2 CH2<br>85.4-88 (298-305)<br>STFRVVSV ><br>STYRVVSV<br>89-96 (306-313)<br>LTVVHQDW ><br>LTVLHQDW<br>118-125 (333-340)<br>EKTISKTK ><br>EKTISKAK	Reduces acid-induced aggregation [178].	**Low ADCC.** Low FcγR binding [178].	**Low CDC.**<br>Low C1q binding [178].<br><br>0
**209**	**Homsap**	**9-G2v8-1**	**CH2**<br>**A93**	**CH2**<br>H93>A (310)	H310A	IGHG2 CH2<br>89-96 (306-313)<br>LTVVHQDW >	**Abrogates FCGRT binding** at pH 6.0 (G2v8 for any amino acid replacement of H93 except cystein) [120]. Number 1 of G2v8-1 is for A.		1 *vamikibart 12560 (128,90).*

						LTVVAQDW			
210	Homsap	5-G2v20	CH2<br>A105	CH2<br>K105>A	K322A	IGHG2 CH2<br>FG 105-117 (322-332)<br>KVSNKG..LPAPI ><br>AVSNKG..LPAPI	**CDC reduction**.<br>Reduces C1q binding.		2 *crizanlizumab 10316 (115,77), cepeprubart 12715 (129,91).*
211	Homsap	9-G2v21	CH2<br>Y15.1,<br>T16,<br>E18	CH2<br>M15.1>Y (252),<br>S16>T (254),<br>T18>E (256)<br>YTE	M252Y,<br>S254T,<br>T256E	IGHG1 CH2<br>13-18 (249-256)<br>DTLMI.SRT ><br>DTLYI.TRE	**Half-life increase.**<br>Enhances FCGRT binding at pH 6.0.		1<br>*omoprubart 12638 (129,91).*
212	Homsap	9-G2v24	CH3<br>L107,<br>S114	CH3<br>M107>L (428),<br>N114>S (434)	M428L,<br>N434S	IGHG2 CH3<br>FG 105-117 (426-437)<br>SVMHEA.LHNHYT ><br>SVLHEA.LHSHYT	**Half-life increase.**<br>Enhances FCGRT binding at pH 6.0.		1<br>*ravulizumab 10659 (117,79).*
213	Homsap	8-G2v36	CH2<br>Q84.4	CH2<br>N84.4>Q (297)	N297Q	IGHG2 CH2<br>83-88 (292-305)<br>REEQFN..STFRVVSV ><br>REEQFQ..STFRVVSV	**ADCC reduction**.<br>Reduces FcγR binding.	Owing to the absence of N-glycosylation at CH2 84.4.	0
214	Homsap	8,10-G2v36-7	CH2<br>A84.3,<br>Q84.4	CH2<br>F84.3>A (296)<br>N84.4>Q (297)	F296A,<br>N297Q	IGHG2 CH2<br>83-88 (292-305)<br>REEQFN..STFRVVSV ><br>REEQAQ..STFRVVSV	**ADCC reduction**.<br>Reduces FcγR binding.	Owing to the absence of N-glycosylation at CH2 84.4.	Eliminates a T cell epitope generated by the Q84.4 aa change.<br>1 *abituzumab 9509 (109,71).*
215	Homsap	15-G2v37	hinge<br>S4	hinge<br>C4>S (219)	C219S	IGHG2 hinge 1-12 (216-230)<br>ERKCCVECPPCP ><br>ERKSCVECPPCP	No disulfide bridge inter H-L		3 *nemolizumab 10064 (112,74)...*

216	Homsap	15-G2v37-1 *chimerisotype G2[hG1S5]*	hinge<br><br>P2,<br>S4,<br>S5,<br>D6,<br>K7,<br>T8,<br>H9,<br>T10	hinge G2 > hinge G1 S5<br>R>P2,<br>C>S4,<br>C>S5,<br>gap>D6,<br>V>K7,<br>gap>T8,<br>E>H9,<br>gap>T10	 <br>R217P,<br>C219S,<br>C220S,<br>gap221D,<br>V222K,<br>gap223T,<br>E224H,<br>gap225T	IGHG2 hinge 1-12 (216-230) > IGHG1 hinge S5 1-15 (216-230)<br>ERKCC .V . E . CPPCP ><br>EPKSCDKTHTCPPCP G1 hinge<br>EPKSSDKTHTCPPCP G1 hinge S5	IGHG2*01 backbone with a G1 hinge S5 (instead of a G2 hinge). No disulfide bridge inter H-L.		1 *abituzumab 9509 (109,71).*
217	Homsap	6-G2v58	CH2<br>S115	CH2<br>A115>S	 A330S	IGHG2 CH2<br>FG 105-117 (322-332)<br>KVSNKG..LPAPI ><br>KVSNKG..LPSPI	**ADCC reduction**.<br>Reduces FcγR binding.	**CDC reduction**.<br>Reduces C1q binding.	0
218	Homsap	6-G2v59	CH2<br>S1.3,<br>T1.2,<br>R1.1	CH2<br>P1.3>S<br>V1.2>T<br>A1.1>R<br>STR	 P234S,<br>V235T,<br>A236R	IGHG2 CH2<br>1.6-3 (231-239)<br>AP.PVAGPS ><br>AP.STRGPS	**ADCC undetectable.**<br>Abrogates FcγR binding [149].	**CDC undetectable.**<br>Abrogates C1q binding [149].	0
219	Homsap	6-G2v60	CH2<br>S115,<br>S116	CH2<br>A115>S (330),<br>P116>S (331)	 A330S,<br>P331S	IGHG2 CH2<br>FG 105-117 (322-332)<br>KVSNKG..LPAPI ><br>KVSNKG..LPSSI	**ADCC reduction**.<br>Reduces FcγR binding.	**CDC reduction**.<br>Reduces C1q binding.	7<br>*tanezumab 8941 (99,61)…*
220	Homsap	6-G2v60-1	CH2<br>S116	CH2<br>P116>S	 P331S	IGHG2 CH2<br>FG 105-117 (322-332)<br>KVSNKG..LPAPI ><br>KVSNKG..LPASI	**ADCC reduction**.<br>Reduces FcγR binding.	**CDC reduction**.<br>Reduces C1q binding.	0
221	Homsap	6-G2v60-66	CH2<br>A27,<br>S115,<br>S116	CH2<br>D27>A (265),<br>A115>S (330),<br>P116>S (331)	 D265A,<br>A330S,<br>P331S	IGHG2 CH2<br>23-31 (261-269)<br>CVVVDVSHE ><br>CVVVDVSAE<br>FG 105-117 (322-332)<br>KVSNKG..LPAPI ><br>KVSNKG..LPSSI	**ADCC reduction**.<br>Reduces FcγR binding.	**CDC reduction**.<br>Reduces C1q binding.	Combines 6-G2v60 and 1-G2v66 (CH2 A27).<br>1<br>*elranatamab 11838 (125,87).*

**222**	**Homsap**	**1-G2v66**	**CH2**<br>**A27**	CH2<br>D27>A (265)	D265A	IGHG2 CH2<br>23-31 (261-269)<br>CVVVDVSHE ><br>CVVVDVSAE	**ADCC reduction**.<br>Reduces FcγR binding.		0
**223**	**Homsap**	**14-G2v72-1**	**hinge**<br>**E5,**<br>**E10**<br>**CH3**<br>**E24**	hinge<br>C5>E,<br>P10>E,<br>CH3<br>L24>E	C220E,<br>P228E<br>L368E	IGHG2 hinge 1-12 (216-230)<br>ERKCCVECPPCP ><br>ERKCEVECPECP<br>IGHG2 CH3<br>12-26 (356-370)<br>EEMT…KNQVSLTCLVK ><br>EEMT…KNQVSLTCEVK	Enhances with 14-G2v73-1 the heterodimerization H-H of bispecific G2 antibodies, by electrostatic steering effects.		1<br>*elranatamab 11838 (125,87).*
**224**	**Homsap**	**14-G2v72-2**	**CH3**<br>**E26,**<br>**E88**	CH3<br>K26>E (370),<br>K88>E (409)	K370E,<br>K409E	IGHG2 CH3<br>12-26 (356-370)<br>EEMT…KNQVSLTCLVK ><br>EEMT…KNQVSLTCLVE<br>83-88 (396-409)<br>PMLDSD..GSFFLYSK ><br>PMLDSD..GSFFLYSE	Enhances with 14-G2v73-2 the heterodimerization H-H of bispecific G2 antibodies, by electrostatic steering effects.		1<br>*navicixizumab 10220 (114,76).*
**225**	**Homsap**	**14-G2v73-1**	**hinge**<br>**R5,**<br>**R7,**<br>**R10,**<br>**CH3**<br>**R88**	hinge<br>C5>R (220),<br>E7>R (224),<br>P10>R (228),<br>CH3<br>K88>R (409)	C220R,<br>E224R,<br>P228R,<br>K409R	IGHG2 hinge 1-12 (216-230)<br>ERKCCVECPPCP ><br>ERKCRVRCPRCP<br>IGHG2 CH3<br>83-88 (396-409)<br>PMLDSD..GSFFLYSK ><br>PMLDSD..GSFFLYSR	Enhances with 14-G2v72-1 the heterodimerization H-H of bispecific G2 antibodies, by electrostatic steering effects.		1<br>*elranatamab 11838 (125,87).*
**226**	**Homsap**	**14-G2v73-2**	**CH3**<br>**K13,**<br>**K84.2**	CH3<br>E13>K (357),<br>D84.2>K (399)	E357K,<br>D399K	IGHG2 CH3<br>12-26 (356-370)<br>EEMT…KNQVSLTCLVK ><br>EEMT…KNQVSLTCLVK<br>83-88 (396-409)<br>PMLDSD..GSFFLYSK ><br>PMLKSD..GSFFLYSK	Enhances with 14-G2v72-2 the heterodimerization H-H of bispecific G2 antibodies, by electrostatic steering effects.		1<br>*navicixizumab 10220 (114,76).*
**227**	**Homsap**	**9-G2v78-2**	**CH3**<br>**A114**	CH3<br>N114>A (434)	N434A	IGHG2 CH3<br>FG 105-117 (426-437)<br>SVMHEA.LHNHYT ><br>SVMHEA.LHAHYT	**Half-life increase.**<br>Enhances FCGRT binding at pH 6.0		1<br>*satralizumab 10065 (114/116,77/78).*

**228**	**Homsap**	**6-G2v94**<br>*chimerisotype G2[11aaG4]*	**CH2**<br>**Q30,**<br>**Y85.2,**<br>**L92,**<br>**S115,**<br>**S116,**<br>**A124**<br>**CH3**<br>**Q11,**<br>**V84,**<br>**R88,**<br>**E98,**<br>**L125.**	CH2<br>H30>Q (268),<br>F85.E>Y (300),<br>V92>L (309),<br>A115>S (330),<br>P116>S (331),<br>T124>A (339)<br><br>R11>Q (355),<br>M84>V (397),<br>K88>R (409),<br>Q98>E (419)<br>P125>L (445)	 H268Q,<br>F300Y,<br>V309L,<br>A115S,<br>P116S,<br>T339A,<br><br>R355Q,<br>M397V,<br>K409R,<br>Q419E,<br>P445L.	IGHG2 CH2<br>23-31 (261-269)<br>CVVVDVSHE ><br>CVVVDVSQE<br>83-88 (292-305)<br>REEQFN..STFRVVSV ><br>REEQFN..STYRVVSV<br>89-96 (306-313)<br>LTVVHQDW ><br>LTVLHQDW<br>FG 105-117 (322-332)<br>KVSNKG..LPAPI ><br>KVSNKG..LPSSI<br>118-125 (333-340)<br>EKTISKTK<br>EKTISKAK<br>IGHG2 CH3<br>7-15 (351-359)<br>LPPSREEMT ><br>LPPSQEEMT<br>83-89 (396-410)<br>PMLDSD..GSFFLYSKL<br>PVLDSD..GSFFLYSRL<br>97-104 (418-425)<br>QQGNVFSC<br>QEGNVFSC<br>118-125 (438-445)<br>QKSLSLSP ><br>QKSLSLSL	**ADCC reduction.**<br>Reduces FcγR binding [179].	**CDC reduction.**<br>Reduces C1q binding [179].	IGHG2*01 backbone with eleven G4 aa, six in the CH2, (Q30, Y85.2, L92, S115, S116, A124) and five in the CH3 (Q11, V84, R88, E98, L125) making the CH2 starting from Q30 and the CH3 identical to those of IGHG4*01.<br>6 *eculizumab 8231 (87,49), samalizumab 9307 (103,65), olendalizumab 10037 (114/116,78), ravulizumab 10659 (117,79), omoprubart 12638 (129,91), ascuprubart 13837 (134,).*
**229**	**Homsap**	**6-G2v95**<br>*chimerisotype G2[4aaG1,3aaG4]*	**CH1**<br>**S10,**<br>**K12,**<br>**G16,**<br>**G17,**<br>**CH2**<br>**Q30,**	CH1<br>C10>S (131),<br>R12>K (133),<br>E16>G (137),<br>S17>G (138),<br>CH2<br>H30>Q (268),	 C131S,<br>R133K,<br>E137G,<br>S138G,<br><br>H268Q,	IGHG2 CH1<br>7-20 (128-141)<br>LAPCSRSTS...ESTAA ><br>LAPSSKSTS...GGTAA<br>CH2<br>23-31 (261-269)<br>CVVVDVSHE >	**ADCC reduction.**<br>Reduces FcγR binding.	**CDC reduction.**<br>Reduces C1q binding.	IGHG2*01 backbone with seven aa changes, four are G1 (CH1 S10, K12, G16, G17) and three are G4

			**CH3**<br>**Q11,**<br>**E98**	CH3<br>R11>Q (355),<br>Q98>E (419)	R355Q,<br>Q419E	CVVVDVSQE<br>CH3<br>7-15 (351-359)<br>LPPSREEMT ><br>LPPSQEEMT<br>95-104 (416-425)<br>RW..QQGNVFSC ><br>RW..QEGNVFSC			(CH2 Q30, CH3 Q11 and E98).<br>2 *nemolizumab 10064 (112,74), satralizumab 10065 (114/116,77/78).*
**230**	**Homsap**	**10-G2v110**	**CHS**<br>**delK2**	CHS<br>K2>del (447)	K447del	IGHG2 CHS<br>1-2 (446,447)<br>GK ><br>G -	Homogeneity of the IGHG2 C-terminal end.		nd
**231**	**Homsap**	**10-G2v111**	**CHS**<br>**delG1,**<br>**delK2**	CHS<br>G1>del (446),<br>K2>del (447)	G446del,<br>K447 del	IGHG2 CHS<br>1-2 (446,447)<br>GK ><br>- -	Homogeneity of the IGHG2 C-terminal end.		nd *nemolizumab 10064 (112,74)...*
**299**<br>*	**Homsap**	**15-G2v37-1**<br>*G2[hG1S5]*,<br>**8,10-G2v36-7**	**G1 hinge S5**<br>**P2,**<br>**S4,**<br>**S5,**<br>**D6,**<br>**K7,**<br>**T8,**<br>**H9,**<br>**T10**<br>**CH2**<br>**A84.3,**<br>**Q84.4**	**G2 hinge > G1 hinge S5**<br>R>P2,<br>C>S4,<br>C>S5 (220),<br>gap>D6,<br>V>K7,<br>gap>T8,<br>E>H9,<br>gap>T10<br>**CH2**<br>F84.3>A (296)<br>N84.4>Q (297)	R217P,<br>C219S,<br>C220S,<br>gap221D,<br>V222K,<br>gap223T,<br>E224H,<br>gap225T<br>F296A,<br>N297Q	IGHG2 hinge 1-12 (216-230) > IGHG1 hinge S5 1-15 (216-230)<br>ERKCC .V . E . CPPCP ><br>EPKSCDKTHTCPPCP G1 hinge<br>EPKSSDKTHTCPPCP G1 hinge S5<br>IGHG2 CH2<br>83-88 (292-305)<br>REEQFN..STFRVVSV ><br>REEQAQ..STFRVVSV	**ADCC reduction.**<br>Reduces FcγR binding. Owing to the absence of N-glycosylation at CH2 84.4.<br>Eliminates a T cell epitope generated by the Q84.4 aa change. (8,10-G2v36-7)	IGHG2*01 with a G1 hinge S5, includes (15-G1v37-1). No disulfide bridge inter H-L. To improve stability.	1 *abituzumab 9509 (109,71).*<br>Association of 15-G2v37-1 *G2[hG1S5]* and 8,10-G2v36-7.
**301**	**Homsap**	**9,10-G3v1**	**CH3**<br>**H115**	CH3<br>R115>H (435)	R435H	IGHG3 CH3<br>FG 105-117 (426-437)<br>SVMHEA.LHNRFT ><br>SVMHEA.LHNHFT	**Half-life increase.**<br>Enhances FCGRT binding at pH 6.0 (half-life comparable to IgG1) [180].		Facilitates binding to protein A.<br>0
**302**	**Homsap**	**5-G3v20**	**CH2**<br>**A105**	CH2<br>K105>A (322)	K322A	IGHG3 CH2<br>FG 105-117 (322-332)<br>KVSNKA..LPAPI ><br>AVSNKA..LPAPI	**CDC reduction.**<br>Reduces C1q binding [181].		0

**303**	**Homsap**	**6-G3v59**	**CH2**<br>**S1.3,**<br>**T1.2,**<br>**R1.1**	CH2<br>L1.3>S (234),<br>L1.2>T (235),<br>G1.1>R (236)<br>STR	L234S, L235T, G236R	IGHG3 CH2<br>1.6-3 (231-239)<br>APELLGGPS ><br>APESTRGPS	**ADCC undetectable.** Abrogates FcγR binding [149].	**CDC undetectable.** Abrogates C1q binding [149].	0
**304**	**Homsap**	**10-G3v110**	**CHS**<br>**delK2**	CHS<br>K2>del (447)	K447del	IGHG3 CHS<br>1-2 (446,447)<br>GK ><br>G -	Homogeneity of the IGHG3 C-terminal end.		nd
**305**	**Homsap**	**10-G3v111**	**CHS**<br>**delG1,**<br>**delK2**	CHS<br>G1>del (446),<br>K2>del (447)	G446del, K447 del	IGHG3 CHS<br>1-2 (446,447)<br>GK ><br>- -	Homogeneity of the IGHG3 C-terminal end.		nd
**401**	**Homsap**	**2-G4v1**	**CH2**<br>**L1.3**	CH2<br>F1.3>L (234)	F234L	IGHG4 CH2<br>1.6-3 (231-239)<br>APEFLGGPS ><br>APELLGGPS	**ADCC enhancement**. Increases FcγRI affinity [101].		0
**402**	**Homsap**	**4-G4v2**	**CH2**<br>**P116**	CH2<br>S116>P (331)	S331P	IGHG4 CH2<br>FG 105-117 (322-332)<br>KVSNKG..LPSSI ><br>KVSNKG..LPSPI	**CDC enhancement** [182].<br>(G1-, G2-, G3-like).		0
**403**	**Homsap**	**6-G4v3**	**CH2**<br>**E1.2**	CH2<br>L1.2>E (235)<br>LE	L235E	IGHG4 CH2<br>1.6-3 (231-239)<br>APEFLGGPS ><br>APEFEGGPS	**ADCC reduction**. Reduces FcγR binding [122].	**CDC reduction**. Reduces C1q binding [122].	16<br>*inclacumab 9512 (106,68)…*
**404**	**Homsap**	**6-G4v3-1**	**CH2**<br>**A1.2**	CH2<br>L1.2>A (235)<br>LA	L235A	IGHG4 CH2<br>1.6-3 (231-239)<br>APEFLGGPS ><br>APEFAGGPS	**ADCC reduction**. Reduces FcγR binding.	**CDC reduction**. Reduces C1q binding.	1 *timolumab 10248 (114,76).*

**405**	**Homsap**	**6-G4v3-44**	**CH2**<br>**E1.2,**<br>**A114**	CH2<br>**L**1.2>**E** (235),<br>**P**114>**A** (329)<br>LEPA	L235E,<br>P329A	IGHG4 CH2<br>1.6-3 (231-239)<br>APEFLGGPS ><br>APEFEGGPS<br>FG 105-117 (322-332)<br>KVSNKG..LPSSI ><br>KVSNKG..LASSI	**ADCC reduction.**<br>Reduces FcγR binding.	**CDC reduction.**<br>Reduces C1q binding.	Combines G4v3 (G4 CH2 E1.2) and G4v44 (G4 CH2 A114).<br>**1** *gamgertamig 13797 (133,95).*
**406**	**Homsap**	**6-G4v3-49**	**CH2**<br>**E1.2,**<br>**G114**	CH2<br>**L**1.2>**E** (235),<br>**P**114>**G** (329)<br>LEPG	L235E,<br>P329G	IGHG4 CH2<br>1.6-3 (231-239)<br>APEFLGGPS ><br>APEFEGGPS<br>FG 105-117 (322-332)<br>KVSNKG..LPSSI ><br>KVSNKG..LGSSI	**ADCC reduction.**<br>Reduces FcγR binding [114].	**CDC reduction.**<br>Reduces C1q binding [114].	Combines 6-G4v3 (G4 CH2 E1.2) and 6-G4v49 (G4 CH2 G114).<br><br>0
**407**	**Homsap**	**6-G4v4**	**CH2**<br>**A1.3,**<br>**A1.2**	CH2<br>**F**1.3>**A** (234),<br>**L**1.2>**A** (235)<br>FALA	F234A,<br>L235A	IGHG4 CH2<br>1.6-3 (231-239)<br>APEFLGGPS ><br>APEAAGGPS	**ADCC reduction.**<br>Reduces FcγR binding [106].	**CDC reduction.**<br>Reduces C1q binding [106].	**30**<br>*dulaglutide 9164 (103,65)...*
**408**	**Homsap**	**12-G4v5**	**hinge**<br>**P10**	hinge<br>**S**10>**P** (228)	S228P	IGHG4 hinge<br>1-12 (216-230)<br>ESKYGPPCPSCP ><br>ESKYGPPCPPCP	Prevents *in vivo* and *in vitro* IgG4 half-IG exchange<br>[183].		**228**<br>*olokizumab 9333 (103,65)...*
**409**	**Homsap**	**12-G4v6**	**CH3**<br>**K88**	**CH3**<br>**R**88>**K**	R409K	IGHG1 CH3<br>85.4-92 (402-413)<br>GSFFLYSRLTVD ><br>GSFFLYSKLTVD	Reduces IgG4 half-IG exchange by stabilizing CH3 interactions [184].<br>IGHG4 CH3 K88 is an allelic polymorphism present in Homsap IGHG4*03 (CH3 codon 69).		**5**<br>*emicizumab, 10115 (113,75)...*
**410**	**Homsap**	**6-G4v7**	**CH2**<br>**delE1.4**<br>**P1.3,**<br>**V1.2,**<br>**A1.1**	CH2<br>**E**1.4>**del** (233)<br>**F**1.3>**P** (234),<br>**L**1.2>**V** (235),<br>**G**1.1>**A** (236)	E233del,<br>F234P,<br>L235V,<br>G236A	IGHG4 CH2<br>1.6-3 (231-239)<br>APEFLGGPS ><br>AP-PVAGPS<br>(G2-like)	**ADCC reduction.**<br>Reduces FcγR binding.	**CDC reduction.**<br>Reduces C1q binding.	**8**<br>*odronextamab 11035 (121,83)...*
**411**	**Homsap**	**6-G4v7-66**	**CH2**<br>**delE1.4,**<br>**P1.3,**<br>**V1.2,**<br>**A1.1,**	CH2<br>**E**1.4>**del** (233),<br>**F**1.3>**P** (234),<br>**L**1.2>**V** (235),<br>**G**1.1>**A** (236),	E233del,<br>F234P,<br>L235V,<br>G236A,	IGHG4 CH2<br>1.6-3 (231-239)<br>APEFLGGPS ><br>AP-PVAGPS<br>23-31 (261-269)	**ADCC reduction.**<br>Reduces FcγR binding.	**CDC reduction.**<br>Reduces C1q binding.	Combines 6-G4v7 and 1-G4v66 (G4 CH2 A27).<br>0

			A27	D27>A (265)	D265A	CVVVDVSQE ><br>CVVVAVSQE			
**412**	**Homsap**	**10-G4v8**	**CH3**<br>**R115,**<br>**F116,**<br>**P125**	CH3<br>H115>R (435),<br>Y116>F (436),<br>L125>P (445)	H435R,<br>Y436F,<br>L445P	IGHG4 CH3<br>FG 105-117 (426-437)<br>SVMHEA.LHNHYT ><br>SVMHEA.LHNRFT<br>118-125 (438-445)<br>QKSLSLSL ><br>QKSLSLSP	Abrogates binding to Protein A<br>(R115 and F116 like G3 alleles, P125 like G1,G2, G3).		8<br>*ubamatamab 11679 (125,87)...*
**413**	**Homsap**	**10-G4v8-1**	**CH3**<br>**P125**	CH3<br>L125>P (445)	L445P	IGHG4 CH3<br>118-125 (438-445)<br>QKSLSLSL ><br>QKSLSLSP	Reduces acid-induced aggregation (stabilizes G4 chains under acidic conditions).		4<br>*lusvertikimab 11533 (124,86)...*
**414**	**Homsap**	**6-G4v9**	**CH2**<br>**P1.4,**<br>**V1.3,**<br>**A1.2**	CH2<br>E1.4>P (233),<br>F1.3>V (234),<br>L1.2>A (235)	E233P,<br>F234V,<br>L235A	IGHG4 CH2<br>1.6-3 (231-239)<br>APEFLGGPS ><br>APPVAGGPS	**ADCC reduction**.<br>Reduces FcγR binding.	**CDC reduction**.<br>Reduces C1q binding.	0
**415**	**Homsap**	**6,1-G4v9-66**	**CH2**<br>**P1.4,**<br>**V1.3,**<br>**A1.2**<br>**A27**	CH2<br>E1.4>P (233),<br>F1.3>V (234),<br>L1.2>A (235)<br>D27>A (265)	E233P,<br>F234V,<br>L235A,<br>D265A	IGHG4 CH2<br>1.6-3 (231-239)<br>APEFLGGPS ><br>APPVAGGPS<br>23-31 (261-269)<br>CVVVDVSQE ><br>CVVVAVSQE	**ADCC reduction**.<br>Reduces FcγR binding.	**CDC reduction**.<br>Reduces C1q binding.	Combines 6-G4v9 (P1.4,V1.3,A1.2) and 1-G4v66 (CH2 A27). *CH2 V92 (309) is allele G4*02 (whereas L 92 (309) is G4*01.*<br>1 *tislelizumab 10553 (117,79).*
**416**	**Homsap**	**18-G4v10**	**CH3**<br>**L85.1,**<br>**K88**	CH3<br>F85.1>L (405),<br>R88>K (409)	F405L,<br>R409K	IGHG4 CH3<br>85.4-92 (402-413)<br>GSFFLYSRLTVD ><br>GSFLLYSKLTVD	Control of half-IG exchange of bispecific IgG4 (only one of the two G4 chains being mutated).		5<br>*teclistamab 10928 (120,82)...*
**417**	**Homsap**	**10,9-G4v11**	**CH1**<br>**Q100,**<br><br>**CH2**<br>**Y84.3**	CH1<br>K100>Q (196),<br><br>CH2<br>F84.3>Y (296)	K196Q,<br><br>F296Y	IGHG4 CH1<br>98-107 (194-203)<br>GTKTYTCNVD ><br>GTQTYTCNVD<br>CH2 84.1-85.1 (294-301)	Two G1 amino acids replacing G4 amino acids (in both chains of bispecific G4 antibodies). CH1 K100>Q reduces Pi and CH2 F84.3>Y increases binding to FCGRT.		3<br>*emicizumab 10115 (113,75)...*

						EQFNSTYR > EQYNSTYR		
**418**	**Homsap**	**14-G4v12**	**CH3** **K12**	E12>K (356)	E356K	IGHG4 CH3 7-15 (351-359) LPPSQEEMT > LPPSQKEMT	Enhances, with 14-G4v13 (CH3 E119), the heterodimerization H-H of bispecific G4 antibodies, via electrostatic interactions.	3 *emicizumab 10115 (113,75)...*
**419**	**Homsap**	**14-G4v13**	**CH3** **E119**	CH3 K119>E (439)	K439E	IGHG4 CH3 FG 105-117 (426-437), 118-119 (438-439) SVMHEA.LHNHYTQK > SVMHEA.LHNHYTQE	Enhances, with 14-G4v12 (CH3 K12), the heterodimerization H-H of bispecific G4 antibodies, via electrostatic interactions.	3 *emicizumab 10115 (113,75)...*
**420**	**Homsap**	**12-G4v17**	**CH1** **S10**	CH1 C10>S (131)	C131S	IGHG4 CH1 1.4-15 (118-136) ASTKGPSVFPLAPCSRSTS > ASTKGPSVFPLAPSSRSTS	Absence of conventional H-L G4 CH1 C10 in crossover V-kappa-G4 CH1.	2 *clesitamig 13237 (131,93).* *evabosmig 13895 (134,)*
**421**	**Homsap**	**9-G4v21**	**CH2** **Y15.1,** **T16,** **E18**	CH2 M15.1>Y (252), S16>T (254), T18>E (256) YTE	M252Y, S254T, T256E	IGHG4 CH2 13-18 (249-256) DTLMISRT > DTLYITRE	**Half-life increase.** Enhances FCGRT binding at pH 6.0 [117].	6 *semorinemab 11024 (120,82)...*
**422**	**Homsap**	**9-G4v22**	**CH2** **T16,** **P91** **CH3** **A114**	CH2 S16>T (254), V91>P (308) CH3 N114>A (434)	S254T V308P N434A	IGHG4 CH2 13-18 (249-256) DTLMISRT > DTLMITRT 89-96 (306-313) LTVLHQDW > LTPLHQDW IGHG4 CH3 FG 105-117 (426-437) SVMHEA.LHNHYT > SVMHEA.LHAHYT	**Half-life increase.** Enhances FCGRT binding at pH 6.0 [185].	1 *pucotenlimab 11561 (124,86).*
**423**	**Homsap**	**9-G4v24**	**CH3** **L107** **S114**	CH3 M107>L (428) N114>S (434)	M428L, N434S	IGHG4 CH3 FG 105-117 (426-437) SVMHEA.LHNHYT > SVLHEA.LHSHYT	**Half-life increase.** Enhances FCGRT binding at pH 6.0.	5 *vensobafusp alfa 12527 (128,90)...*
**424**	**Homsap**	**8-G4v30**	**CH2**	CH2		IGHG4 CH2	**ADCC reduction.** Owing to the	1 *efmarodocokin alfa*

			G84.4	N84.4>G (297)	N297G	83-88 (292-305)<br>REEQFN..STYRVVSV ><br>REEQFG..STYRVVSV	Reduces FcγR binding.	absence of N-glycosylation at CH2 84.4.	*11266 (123,85).*
**425**	**Homsap**	**14-G4v32**	**CH3**<br>**W22**	CH3<br>T22>W (366)	T366W	IGHG4 CH3<br>20-26 (364-370)<br>SLTCLVK ><br>SLWCLVK	**Knob (W22)** of knobs-into-holes (KIH) which promotes the heterodimerization of the CH3 of two different H-gamma4 chains. The hole is 14-G4v33.		**5**<br>*rezetamig*<br>*12767 (129,91)...*
**426**	**Homsap**	**14-G4v33**	**CH3**<br>**S22,**<br>**A24,**<br>**V86**	CH3<br>T22>S (366),<br>L24>A (368),<br>Y86>V (407)	T366S,<br>L368A,<br>Y407V	IGHG4 CH3<br>20-26 (364-370)<br>SLTCLVK ><br>SLSCAVK<br>85.4-92 (402-413)<br>GSFFLYSRLTVD ><br>GSFFLVSRLTVD	**Hole (SAV)** of knobs-into-holes (KIH) which promotes the heterodimerization of the CH3 of two different H-gamma4 chains. The knob is 14-G4v32.		**5**<br>*rezetamig*<br>*12767 (129,91)...*
**427**	**Homsap**	**6-G4v34**	**CH2**<br>**A1.3,**<br>**A1.2,**<br>**A1,**<br>**S2**	CH2<br>F1.3>A (234),<br>L1.2>A (235),<br>G1>A (237),<br>P2>S (238)<br>G4sigma1	F234A,<br>L235A,<br>G237A,<br>P238S	IGHG4 CH2<br>1.6-3 (231-239)<br>APEFLGGPS ><br>APEAAGASS	**ADCC reduction.**<br>Reduces FcγR binding. Undetectable ADCC and ADCP [167].	**CDC reduction.**<br>Reduces C1q binding.<br>Undetectable CDC [167].	**3D**<br>5w5m [167]<br>0
**428**	**Homsap**	**6-G4v35**	**CH2**<br>**A1.3,**<br>**A1.2,**<br>**delG1.1,**<br>**A1,**<br>**S2**	CH2<br>F1.3>A (234),<br>L1.2>A (235),<br>G1.1>del (236)<br>G1>A (237),<br>P2>S (238)<br>G4sigma2	F234A<br>L235A,<br>G236del<br>G237A,<br>P238S	IGHG4 CH2<br>1.6-3 (231-239)<br>APEFLGGPS ><br>APEAA-ASS	**ADCC reduction.**<br>Reduces FcγR binding. Undetectable ADCC and ADCP [167].	**CDC reduction.**<br>Reduces C1q binding.<br>Undetectable CDC [167].	**3D**<br>5w5n [167]<br>0
**429**	**Homsap**	**8-G4v36**	**CH2**<br>**Q84.4**	CH2<br>N84.4>Q (297)	N297Q	IGHG4 CH2<br>83-88 (292-305)<br>REEQFN..STYRVVSV ><br>REEQFQ..STYRVVSV	**ADCC reduction.**<br>Reduces FcγR binding.	Owing to the absence of N-glycosylation at CH2 84.4 [129]	**1**<br>*vixarelimab*<br>*11350 (123,85)*
**430**	**Homsap**	**9-G4v42**	**CH2**<br>**Q14,**<br><br><br><br>**CH3**	CH2<br>T14>Q (250),<br><br><br><br>CH3	T250Q,	IGHG4 CH2<br>13-18 (249-256)<br>DTLMISRT ><br>DQLMISRT<br>CH3	**Half-life increase.**<br>Enhances FCGRT binding at pH 6.0		**2**<br>*efdelikofusp alfa*<br>*13122 (131,93),*<br>*efzilonkofusp alfa*<br>*13123 (131,93).*

			L107	M107>L (428)	M428L	FG 105-117 (426-437)<br>SVMHEA.LHNHYT ><br>SVLHEA.LHNHYT			
431	**Homsap**	**6-G4v44**	**CH2**<br>**A114**	CH2<br>P114>A (329)	P329A	IGHG4 CH2<br>FG 105-117 (322-332)<br>KVSNKG..LPSSI ><br>KVSNKG..LASSI	**ADCC reduction.**<br>Reduces FcγR binding.	**CDC reduction.**<br>Reduces C1q binding.	0<br>part of 6-G4v3-44
432	**Homsap**	**6-G4v49**	**CH2**<br>**G114**	CH2<br>P114>G (329)	P329G	IGHG4 CH2<br>FG 105-117 (322-332)<br>KVSNKG..LPSSI ><br>KVSNKG..LGSSI	**ADCC reduction.**<br>Reduces FcγR binding [114].	**CDC reduction.**<br>Reduces C1q binding [114].	0
433	**Homsap**	**1-G4v50**	**CH2**<br>**delE1.4**<br>**P1.3,**<br>**V1.2,**<br>**A1.1**	CH2<br>E1.4>del (233),<br>F1.3>P (234),<br>L1.2>V (235),<br>G1.1>A (236)	E233del,<br>F234P,<br>L235V,<br>G236A	IGHG4 CH2<br>1.6-3 (231-239)<br>APEFLGGPS ><br>AP-PVAGPS<br>(G2-like)	**ADCC reduction.**<br>Decreases FcgammaR binding.		**1**<br>*olsutamig*<br>*13678 (133,95).*
434	**Homsap**	**17-G4v57-1**	**CH1**<br>**K84.2**	CH2<br>Q84.2>K	Q175K	IGHG4 CH1<br>23-30 (144-151)<br>CLVKDYFP (no aa change K26)<br>IGHG4 CH1<br>83-88 (172-185)<br>AVLQSS..GLYSLSSV ><br>AVLKSS..GLYSLSSV	Enhances (K26 no aa change, K84.2), with 17-KCv57-1 (S20>E, T90>E), the heteropairing H_L-kappa of bispecific G4-kappa/lambda antibodies.		**1**<br>*zemocimig*<br>*13346_F9 (132,94).*
435	**Homsap**	**17-G4v57-2**	**CH1**<br>**E26,**<br>**E84.2**	CH1<br>K26>E,<br>Q84.2>E	K147E,<br>Q175E	IGHG4 CH1<br>23-30 (144-151)<br>CLVKDYFP ><br>CLVEDYFP<br>IGHG4 CH1<br>83-88 (172-185)<br>AVLQSS..GLYSLSSV ><br>AVLESS..GLYSLSSV	Enhances, with 17-LC2v57-2 (K20, K90), the heteropairing H_L-lambda of bispecific G4-kappa/lambda antibodies.		**1**<br>*zemocimig*<br>*13346_F10 (132,94).*
436	**Homsap**	**17-G4v57-4**	**CH1**<br>**S10,**<br>**C84**	CH1<br>C10>S,<br>V84>C	C131S,<br>V173C	IGHG4 CH1<br>1.4-15 (118-136)<br>ASTKGPSVFPLAPCSRSTS ><br>ASTKGPSVFPLAPSSRSTS<br>IGHG4 CH1	Enforces, with 17-KCv57-4 (C79, S126) in a shifted CH1-CL cognate disulfide bond CH1 C84-CL79 (in place of CH1 C10-CL126), the heteropairing H_L-kappa of bispecific G4-kappa antibodies.		**1**<br>*evabosmig*<br>*13895 (134,).*

						83-88 (172-185)<br>AVLQSS..GLYSLSSV ><br>ACLQSS..GLYSLSSV			
**437**	**Homsap**	**6-G4v59**	**CH2**<br>**S1.3,**<br>**T1.2,**<br>**R1.1**	CH2<br>**F**1.3>**S**<br>**L**1.2>**T**<br>**G**1.1>**R**<br>STR	F234S,<br>L235T,<br>G236R	IGHG4 CH2<br>1.6-3 (231-239)<br>APEFLGGPS ><br>APESTRGPS	**ADCC undetectable.**<br>Abrogates FcγR binding [149].	**CDC undetectable.**<br>Abrogates C1q binding [149].	0
**438**	**Homsap**	**1-G4v66**	**CH2**<br>**A27**	CH2<br>**D**27>**A** (265)<br>DA	D265A	IGHG4 CH2<br>23-31 (261-269)<br>CVVVDVSQE ><br>CVVVAVSQE	**ADCC reduction.**<br>Reduces FcγR binding.		A27 disrupts a major FcγR contact.<br>0
**439**	**Homsap**	**14-G4v74**	**CH3**<br>**C10**	CH3<br>**S**10>**C** (354)	S354C	IGHG4 CH3<br>1.4-12 (341-356)<br>GQPREPQVYTLPPSQE ><br>GQPREPQVYTLPPCQE >	Promotes, with G4v75, the heterodimerization H-H of bispecific G4 antibodies.		**1**<br>*gamgertamig 13797 (133,95).*
**440**	**Homsap**	**14-G4v75**	**CH3**<br>**C5**	CH3<br>**Y**5>C (349)	Y349C	IGHG4 CH3<br>1.4-12 (341-356)<br>GQPREPQVYTLPPSQE ><br>GQPREPQVCTLPPSQE >	Promotes, with G4v74, the heterodimerization H-H of bispecific G4 antibodies.		**1**<br>*gamgertamig 13797 (133,95).*
**441**	**Homsap**	**9-G4v78**	**CH3**<br>**L107,**<br>**A114**	CH3<br>**M**107>L (428),<br>**N**114>A (434)	M428L,<br>N434A	IGHG4 CH3<br>FG 105-117 (426-437)<br>SVMHEA.LHNHYT ><br>SVLHEA.LHAHYT	**Half-life increase.**<br>Enhances FCGRT binding at pH 6.0.		**1**<br>*zemocimig 13346 (132,94).*
**442**	**Homsap**	**10-G4v83**	**CH3**<br>**R115,**<br>**F116,**	CH3<br>**H**115>**R** (435),<br>**Y**116>**F** (436),	H435R,<br>Y436F,	IGHG4 CH3<br>FG 105-117 (426-437)<br>SVMHEA.LHNHYT ><br>SVMHEA.LHNRFT	Abrogates binding to Protein A.		**2**<br>*odronextamab 11035 (121,83).*<br>*evabosmig 13895 (134,).*
**443**	**Homsap**	**10-G4v83-1**	**CH3**<br>**R115**	CH3<br>**H**115>**R** (435)	H435R	IGHG4 CH3<br>FG 105-117 (426-437)<br>SVMHEA.LHNHYT ><br>SVMHEA.LHNRYT	Abrogates binding to Protein A.		**2** *emicizumab 10115 (113,75), safimestomig 13102 (131,93).*

**444**	**Homsap**	**10-G4v89**	**CH3**<br>**R118,**<br>**E120**	**CH3**<br>Q118>R (438),<br>S440>E (440)	 Q438R,<br>S440E	IGHG4 CH3<br>118-125 (438-445)<br>QKSLSLSP ><br>RKELSLSP	Suppresses rheumatoid factor binding.		**1**<br>*zemocimig 13346 (132,94).*
**445**	**Homsap**	**10-G4v90**<br>*chimerisotype G4[6aaG1]*	**CH3**<br>**R11,**<br>**D12,**<br>**L14,**<br>**K88,**<br>**Q98,**<br>**P125**	**CH3**<br>Q11>R (355),<br>E12>D (356),<br>M14>L (358),<br>R88>K (409),<br>E98>Q (419),<br>L125>P (445).	 Q355R,<br>E356D,<br>M358L,<br>R409K,<br>E419Q,<br>L445P	IGHG4 CH3<br>1-15 (345-359)<br>EPQVYTLPPSQEEMT ><br>EPQVYTLPPSRDELT ><br>85-100 (406-421)<br>LYSRLTVDKSRW..QEGN ><br>LYSKLTVDKSRW..QQGN	The backbone is IGHG4*01 with six G1 aa in the CH3 (R11, D12, L14, K88, Q98, P125). CH3 D12 and L14 are allotypic aa present in IGHG1*01.	CH3 K88 (12-G4v6) reduces IgG4 half-IG exchange [141]. IGHG4 CH3 K88 is an allelic polymorphism present in Homsap IGHG4*03 (CH3 codon 69).	**1**<br>*vixarelimab 11350 (123,85).*
**446**	**Homsap**	**10-G4v110**	**CHS**<br>**delK2**	CHS<br>K2>del (447)	 K447del	IGHG4 CHS<br>1-2 (446,447)<br>GK ><br>G -	Homogeneity of the IGHG4 C-terminal end.		nd
**447**	**Homsap**	**10-G4v111**	**CHS**<br>**delG1,**<br>**delK2**	CHS<br>G1>del (446),<br>K2>del (447)	 G446del,<br>K447del	IGHG4 CHS<br>1-2 (446,447)<br>GK ><br>- -	Homogeneity of the IGHG4 C-terminal end.		nd<br>*tibulizumab 10056 (117,79)...*
**448**	**Homsap**	**12-G4v112**	**CH1**<br>**insS1.6,**<br>**insS1.5**			IGHG4<br>-- ><br>SS	2-mer diseryl insertion (1.6, 1.5) upstream of IGHG4 CH1 in crossover V-kappa-G4 CH1		**1**<br>*clesitamig 13237 (131,93).*
**496**<br>*	**Homsap**	**12-G4v5,**<br>**10-G4v90**<br>*G4[6aaG1]*,<br>**8-G4v36,**	**hinge**<br>**P10,**<br>**CH2**<br>**Q84.4,**<br>**CH3**<br>**R11,**<br>**D12,**<br>**L14,**<br>**K88,**<br>**Q98,**<br>**P125**	hinge<br>S10>P (228)<br>**CH2**<br>N84.4>Q (297)<br>**CH3**<br>Q11>R (355),<br>E12>D (356),<br>M14>L (358),<br>R88>K (409),<br>E98>Q (419),<br>L125>P (445).	 S228P,<br><br>N297Q,<br><br>Q355R,<br>E356D,<br>M358L,<br>R409K,<br>E419Q,<br>L445P	IGHG4 hinge<br>1-12 (216-230)<br>ESKYGPPCPSCP ><br>ESKYGPPCPPCP<br>CH2<br>83-88 (292-305)<br>REEQFN..STYRVVSV ><br>REEQFQ..STYRVVSV<br>CH3<br>1-15 (345-359)<br>EPQVYTLPPSQEEMT ><br>EPQVYTLPPSRDELT >	Prevents *in vivo* and *in vitro* IgG4 half-IG exchange [183]. (12-G4v5)<br>**ADCC and CDC reduction.** Reduces FcγR and C1q binding.<br>Owing to the absence of N-glycosylation at CH2 84.4 [129].<br>(8-G4v36)	IGHG4*01 backbone with six G1 aa in the CH3, making the CH3 identical to that of IGHG1*01.<br>*CH3 D12 and L14 are allotypic aa present in IGHG1*01.*<br>(10-G4v90)	**1**<br>*vixarelimab 11350 (123,85).*

						85-100 (406-421)<br>LYSRLTVDKSRW..QEGN ><br>LYSKLTVDKSRW..QQGN<br>118-125 (438-445)<br>QKSLSLSL ><br>QKSLSLSP			
**497**<br>*****	**Homsap**	**12-G4v5,**<br>**6-G4v3**	**hinge**<br>**P10,**<br>**CH2**<br>**E1.2**	hinge<br>S10>P (228),<br>CH2<br>L1.2>E (235)<br>SPLE	S228P,<br><br><br>L235E	IGHG4 hinge<br>1-12 (216-230)<br>ESKYGPPCPSCP ><br>ESKYGPPCPPCP<br>CH2<br>1.6-3 (231-239)<br>APEFLGGPS ><br>APEFEGGPS	**ADCC reduction**.<br>Reduces FcγR binding (6-G4v3) [122].	**CDC reduction**.<br>Reduces C1q binding (6-G4v3) [122].	Prevents *in vivo* and *in vitro* IgG4 half-IG exchange (12-G4v5) [183].<br>Association of 6-G4v3 and 12-G4v5.<br>(16)
**498**<br>*****	**Homsap**	**12-G4v5,**<br>**6-G4v3-49**	**hinge**<br>**P10,**<br>**CH2**<br>**E1.2,**<br>**G114**	hinge<br>S10>P (228),<br>CH2<br>L1.2>E (235),<br>P114>G (329)<br>SPLEPG	S228P,<br><br><br>L235E,<br>P329G	IGHG4 hinge<br>1-12 (216-230)<br>ESKYGPPCPSCP ><br>ESKYGPPCPPCP<br>CH2<br>1.6-3 (231-239)<br>APEFLGGPS ><br>APEFEGGPS<br>FG 105-117 (322-332)<br>KVSNKG..LPSSI ><br>KVSNKA..LGSSI	**ADCC reduction**.<br>Reduces FcγR binding (6-G4v3-49) [114].	**CDC reduction**.<br>Reduces C1q binding [121] (6-G4v3-49) [114].	Prevents *in vivo* and *in vitro* IgG4 half-IG exchange (12-G4v5) [183].<br>Association of 6-G4v3-49 and 12-G4v5.<br>(0)
**499***	**Homsap**	**12-G4v5, 6-G4v4**	**hinge**<br>**P10,**<br>**CH2**<br>**A1.3,**<br>**A1.2**	hinge<br>S10>P (228),<br>CH2<br>F1.3>A (234),<br>L1.2>A (235)<br>IgG4 ProAlaAla,<br>SPFALA	S228P,<br><br><br>F234A,<br>L235A	IGHG4 hinge<br>1-12 (216-230)<br>ESKYGPPCPSCP ><br>ESKYGPPCPPCP<br>CH2<br>1.6-3 (231-239)<br>APEFLGGPS ><br>APEAAGGPS	**ADCC reduction**.<br>Reduces FcγR binding (6-G4v4) [106].	**CDC reduction**.<br>Reduces C1q binding (6-G4v4) [106].	Prevents *in vivo* and *in vitro* IgG4 half-IG exchange (12-G4v5) [183].<br>Association of 6-G4v4 and 12-G4v5.<br>(30)

**501**	**Homsap**	**17-Kv84-1**	**V-KAPPA**<br>**K44**<br>**C-KAPPA**<br>**E22**	V-KAPPA<br>Q44>K<br>C-KAPPA<br>V22>E (133)	Q44K<br><br>V133E	VK 39-46<br>*LNWYQQKP* ><br>*LNWYQKKP*<br>IGKC 16-26 (127-137)<br>SGTASVVCLLN ><br>SGTASVECLLN	Enhances the pairing of Kv84-1 L-kappa with 17-G1v84-1 VH-CH1 in bispecific G1 kappa.	1 *linclatamig 13026 (130,92).*
**502**	**Homsap**	**17-Kv84-2**	**V-KAPPA**<br>**E44**<br>**C-KAPPA**<br>**K22**	V-KAPPA<br>Q44>E<br>C-KAPPA<br>V22>K (133)	Q44E<br><br>V133K	VK 39-46<br>*LNWYQQKP* ><br>*LNWYQEKP*<br>IGKC 16-26 (127-137)<br>SGTASVVCLLN ><br>SGTASVKCLLN	Enhances the pairing of Kv84-2 L-kappa with 17-G1v84-2 VH-CH1 in bispecific G1 kappa.	1 *linclatamig 13026 (130,92).*
**503**	**Homsap**	**17-Kv92-1**	**V-KAPPA**<br>**K44**<br>**C-KAPPA**<br>**K3**	V-KAPPA<br>Q44>K<br>C-KAPPA<br>S3>K (114)	Q44K<br><br>S114K	VK 39-46<br>*LSWYQQKP* ><br>*LSWYQKKP*<br>IGKC 1.4-6 (108-117)<br>RTVAAPSVFI ><br>RTVAAPKVFI	Enhances the pairing of 17-Kv92-1 L-kappa with<br>17-G1v92-1 VH-CH1 in bispecific G1 kappa.	1 *spevatamig 13260_L (131,93).*
**504**	**Homsap**	**17-Kv92-2**	**V-KAPPA**<br>**E44**<br>**C-KAPPA**<br>**E3**	V-KAPPA<br>Q44>E<br>C-KAPPA<br>S3>E (114)	Q44E<br><br>S114E	VK 39-46<br>*LTWYQQKP* ><br>*LTWYQEKP*<br>IGKC 1.4-6 (108-117)<br>RTVAAPSVFI ><br>RTVAAPEVFI	Enhances the pairing of 17-Kv92-2 L-kappa with<br>17-G1v92-2 VH-CH1 in bispecific G1 kappa.	1 *spevatamig 13260_N (131,93).*
**525**	**Homsap**	**15-KCv36**	**C-KAPPA**<br>**S126**	C-KAPPA<br>C126>S	C214S	IGKC<br>118-126 (206-214)<br>TKSFNRGEC ><br>TKSFNRGES	Absence of the CL126 inter H-L.	5<br>*naptumomab estafenatox 8598 (96,58)…*
**526**	**Homsap**	**17-KCv37**	**C-KAPPA**<br>**C121,**<br>**S126**	C-KAPPA<br>F121>C<br>C126>S	F209C<br>C214S	IGKC<br>118-126 (206-214)<br>TKSFNRGEC ><br>TKSCNRGES	Promotes the H-L pairing of 17-KCv37 (C121, S126) with 17-G1v93 (CH1 C12), in the absence of the CL126 inter H-L.	1<br>*spevatamig 13260 (131,93).*
**527**	**Homsap**	**17-KCv57**	**C-KAPPA**<br>**R12,**<br>**K13**	C-KAPPA<br>E12>R,<br>Q13>K	E123R,<br>Q124K	IGKC<br>10-15 (121-126)<br>SDEQLK ><br>SDRKLK	Enhances, with 17-G1v57, the heteropairing H-L of bispecific G1-kappa antibodies.	9<br>*glofitamab 11145 (121,83)…*

528	Homsap	17-KCv57-1	C-KAPPA<br>E20,<br>E90	C-KAPPA<br>S20>E,<br>E90>E	S131E,<br>T180E	IGKC<br>16-26 (127-137)<br>SGTASVVCLLN ><br>SGTAEVVCLLN<br>85.4-96 (171-186)<br>STYSLSSTLTLSKADY ><br>STYSLSSTLELSKADY	Enhances, with G4v57-1 (CH1 K84.2), the heteropairing H_L-kappa of bispecific G1-kappa/lambda antibodies.	1<br>*zemocimig 13346 (132,94).*
529	Homsap	17-KCv57-3	C-KAPPA<br>K20	S20>K (131)	S131K	IGKC<br>16-26 (127-137)<br>SGTASVVCLLN ><br>SGTAKVVCLLN	Enhances, with 17-G1v57-3 (CH1 D26), the heteropairing H_L-kappa of bispecific G1-kappa antibodies.	1<br>*tuvonralimab 11841 (125,87).*
530	Homsap	17-KCv57-4	C-KAPPA<br>C79,<br>S126	C-KAPPA<br>Q79>C (160)<br>C214>S (214)	Q160C,<br>C214S	IGKC<br>77-84 (158-165)<br>NSQESVTE ><br>NSCESVTE<br>118-126 (206-214)<br>TKSFNRGEC ><br>TKSFNRGES	Enforces, with 17-G4v57-4 (S10, C84) in a shifted CH1-CL cognate disulfide bond CH1 C84-CL79 (in place of CH1 C10-CL126), the heteropairing H_L-kappa of bispecific G4-kappa antibodies.	1<br>*evabosmig 13895 (134,).*
531	Homsap	17-KCv68	C-KAPPA<br>R20,<br>G22,<br>R86	C-KAPPA<br>S20>R (131),<br>V22>G (133),<br>S86>R (176)	S131R,<br>V133G,<br>S176R	IGKC<br>16-26 (127-137)<br>SGTASVVCLLN ><br>SGTARVGCLLN<br>85.4-96 (171-186)<br>STYSLSSTLTLSKADY ><br>STYSLRSTLTLSKADY	Enhances, with 17-G1v68-1 (CH1 E7, T26, E84.2), the heteropairing H_L-kappa of bispecific G1-kappa/lambda antibodies.	1<br>*reozalimab 11904 (126,88).*
532	Homsap	17-KCv84	C-KAPPA<br>C79,<br>C81	C-KAPPA<br>Q79>C (160),<br>S81>C (162)	Q160C,<br>S162C	IGKC<br>77-84 (158-165)<br>NSQESVTE ><br>NSCECVTE	Promotes the pairing of 17-KCv84 (C79, C81) with 17-G1v84 of a G1-kappa antibody. Antibody expressed with another antibody (without AA changes) in the same cell.	1<br>*tuvonralimab 11841 (125,87).*
533	Homsap	16-KCv93	C-KAPPA<br>C93	C-KAPPA<br>K93>C (183)	K183C	IGKC<br>85.4-96 (171-186)<br>STYSLSSTLTLSKADY ><br>STYSLSSTLTLSCADY	**Conjugation site-specific**.<br>Site-specific drug attachment engineered cysteine.	2<br>*micvotabart 13004 (130,92), micvotabart pelidotin 13003 (130,92).*

534	Homsap	16-KCv93-1	C-KAPPA C117	C-KAPPA V117>C (205)	V205C	IGKC 105-117 (195-205) EVTHQG..LSSPV > EVTHQG..LSSPC	**Conjugation site-specific**. Site-specific drug attachment engineered cysteine.		2 *zevontabart 13956 (134,), zevontabart vedotin 13957 (134,).*
535	Homsap	12-KCv96	C-KAPPA A1.4, S1.3	C-KAPPA R1.4>A (108) T1.3>S (109)	R108A T109S	IGKC 1.4-6 (108-117) RTVAAPSVFI > ASVAAPSVFI	IGKC variant in elbow of crossover VH-C-kappa.		10 *vanucizumab 9950 (111/113,73)…*
536	Homsap	17-KCv100-1	C-KAPPA N87	C-KAPPA S87>N (177)	S177N	IGKC 85.4-96 (171-186) STYSLSSTLTLSKADY > STYSLSNTLTLSKADY	Enhances with 17-G1v100-1 (CH1 E16) the heteropairing H-L-kappa of a Fab.	N87 is a polymorphism (IGKC*05, AF113887) found in an amyloidogenic kappa chain [187].	1 *velaprumig 13342_L (132).*
537	Homsap	10-KCv113	C-KAPPA InsR1.7, InsT1.6, InsV1.5			IGKC 1.7-6 (108C-117) - - - RTVAAPSVFI > RTVRTVAAPSVFI	3-mer insertion upstream of IGKC (by duplication of RTV 1.4, 1.3, 1.2).	Higher isoelectric point favors better production.	0
570	Homsap	12-KVv1	V-KAPPA T125, V126, S127, InsS128	V-KAPPA E125>T (114), I126>V (115), K127>S (116), InsS128 (117)	E114T I115V, K116S, InsS117	V-KAPPA/VH 125-128 (114-117) E I K - > TVSS	V-KAPPA variant ('TVSS' IGHJ motif instead of 'EIK' IGKJ motif) in elbow of crossover V-kappa-G1 CH1.		1 *torutamig 13997 (134,).*
575	Homsap	10-LC2v1	C-LAMBDA2 G45, K82	C-LAMBDA2 S45>G (152) T82>K (165)	S152G, T163K	IGLC2 41-84 (148-165) WKADSSPVKA..GVETTTPS > WKADGSPVKA..GVETTKPS	Improves thermal stability		1 *regdanvimab 11883 (124,85).*
576	Homsap	17-LC2v57-2	C-LAMBDA2 K20, K90	C-LAMBDA2 T20>K, S90>K	T131K, S180K	IGLC2 16-26 (127-137) ANKATLVCLIS > ANKAKLVCLIS 89-96 (179-186) LSLTPEQW > LKLTPEQW	Enhances, with G4v57-2 (CH1 E26, E84.2), the heteropairing H_L-lambda2 of bispecific G1-kappa/lambda antibodies.		1 *zemocimig 13346_F10 (132).*
577	Homsap	17-LC2v58	C-LAMBDA2 C10,	C-LAMBDA2	S121C,	IGLC2 1.5-15 (107A-126)	Promotes, with 17-G1v58 (CH1 C5), the heteropairing H-L of bispecific G1-lambda2		5

			V126	S10>C (121), C126>V (214)	C214V	GQPKAAPSVTLFPPSSEELQ ><br>GQPKAAPSVTLFPPCSEELQ<br>118-127 (206-215)<br>EKTVAPTECS ><br>EKTVAPTEVS	antibodies by an alternative interchain cysteine mutations which creates a novel disulfide bridge H CH1 C5 - L C10 instead of hinge C5- L C126) [147].	*volrustomig 12168 (127,89)...*
**578**	**Homsap**	**17-LC2v58-1**	**C-LAMBDA2**<br>**R5,**<br>**C10,**<br>**V126**	C-LAMBDA2<br>T5>R (116),<br>S10>C (121),<br>C126>V (214)	T116R,<br>S121C,<br>C214V	IGLC2<br>1.5-15 (107A-126)<br>GQPKAAPSVTLFPPSSEELQ ><br>GQPKAAPSVRLFPPCSEELQ<br>118-127 (206-215)<br>EKTVAPTECS ><br>EKTVAPTEVS	Promotes, with 17-G1v58-1 (CH1 D20) or 17-G1v58-2 (CH1 E20), the heteropairing H-L of bispecific G1-lambda2 antibodies by an alternative interchain cysteine mutations (see LC2v58) [148], which is enhanced by LC2v58-1 R5 electrostatic steering interaction with 17-G1v58-1 (CH1 D20) or 17-G1v58-2 (CH1 E20), respectively [148].	**3D**<br>8tjf, 8tiu<br>0
**579**	**Homsap**	**17-LC2v69**	**C-LAMBDA2**<br>**E86,**<br>**E88,**<br>**A124**	C-LAMBDA2<br>S86>E (176),<br>Y88>E (178),<br>T124>A (212)	S176E,<br>Y178E,<br>T212A	IGLC2<br>85.4-96 (171-186)<br>NKYAASSYLSLTPEQW<br>NKYAAESELSLTPEQW<br>118-127 (206-215)<br>EKTVAPTECS ><br>EKTVAPAECS	Enhances, with 17-G1v69-1 (CH1 K86), the heteropairing H_L-lambda of bispecific G1-kappa/lambda antibodies.	**1**<br>*reozalimab 11904 (126,88).*
**580**	**Homsap**	**10-LC3v1**	**C-LAMBDA3**<br>**S1.5,**<br>**R119,**<br>**L123,**<br>**delS127**	C-LAMBDA3<br>G1.5>S (107A),<br>K119>R (207),<br>P123>L (211),<br>S127>del(215)	G107AS,<br>K207R,<br>P211L,<br>S215del	IGLC3<br>1.5-15 (107A-126)<br>GQPKAAPSVTLFPPSSEELQ ><br>SQPKAAPSVTLFPPSSEELQ<br>118-127 (206-215)<br>EKTVAPTECS ><br>ERTVALTEC-	Increases stability of disulfide bond between lambda type light chain and heavy chain.	**1**<br>*opucolimab 11310 (122,84).*
**601**	**Canlupfam**	**6-G2v1**	**CH2**<br>**A1.3,**<br>**A1.2,**<br>**A1**	CH2<br>M1.3>A (234),<br>L1.2>A (235),<br>G1>A (237)	M234A,<br>L235A,<br>G237A	Canlupfam IGHG2 CH2<br>1.6-3 (231-239)<br>APEMLGGPS ><br>APEAAGAPS	**ADCC reduction.** Reduces FcγR binding.<br>**CDC reduction.** Reduces C1q binding.	**3**<br>*bedinvetmab 11027 (120,82)...*<br>.
**602**	**Canlupfam**	**6-G2v2**	**CH2**<br>**A1.3,**<br>**A1.2,**<br>**G114**	CH2<br>M1.3>A (234),<br>L1.2>A (235),<br>P114>G (329)	M234A,<br>L235A,<br>P329G	Canlupfam IGHG2 CH2<br>1.6-3 (231-239)<br>APEMLGGPS ><br>APEAAGGPS<br>FG 105-117 (322-332)	**ADCC reduction.** Reduces FcγR binding.<br>**CDC reduction.** Reduces C1q binding.	**1**<br>*nolavetbart 12563 (128,90).*

						KVNNKA..LPSPI ><br>KVNNKA..LGSPI			
**603**	**Canlupfam**	**8-G2v29**	**CH2**<br>**A84.4**	CH2<br>N84.4>A (297)	N297A	Canlupfam IGHG1 CH2<br>83-86 (292-303)<br>REEQFN..GTYRVV ><br>REEQFA..GTYRVV	**ADCC reduction**.<br>Reduces FcγR binding.	Owing to the absence of N-glycosylation at CH2 84.4 [129].	part of 8,6-G2v29-66.<br>0
**604**	**Canlupfam**	**8,6-G2v29-66**	**CH2**<br>**A27**<br>**A84.4**	CH2<br>D27>A (265)<br>N84.4>A (297)	D265A,<br>N297A	Canlupfam IGHG2 CH2<br>23-31 (261-269)<br>CVVVDLDPE ><br>CVVVAVSHE<br>83-86 (292-303)<br>REEQFN..GTYRVV ><br>REEQFA..GTYRVV	**ADCC reduction**.<br>Reduces FcγR binding.	**CDC reduction**.<br>Reduces C1q binding	Combines 8-G2v29 (CH2 A84.4, no N-glycosylation) and 1-G2v66 (CH2 A27).<br>**1** *gilvetmab 10528 (116,78)*.
**605**	**Canlupfam**	**1-G2v66**	**CH2**<br>**A27**	CH2<br>D27>A (265)	D265A	Canlupfam IGHG2 CH2<br>23-31 (261-269)<br>CVVVDLDPE ><br>CVVVAVSHE	**ADCC reduction**.<br>Reduces FcγR binding.		part of 8,6-G2v29-66.<br>0
**606**	**Canlupfam**	**9-G2v24**	**CH3**<br>**H114**	CH3<br>N114>H (434)	N434H	Canlupfam IGHG2 CH3<br>FG 105-117 (426-437)<br>AVMHEA.LHNHYT ><br>AVMHEA.LHHHYT	**Half-life increase.**<br>Enhances FCGRT binding at pH 6.0.		**1** *riltovetbart 12311 (127,89)*
**646**	**Musmus**	**6-G2Av1**	**CH2**<br>**A1.3,**<br>**A1.2**	CH2<br>L1.3>A (234),<br>L1.2>A (235)<br>LALA	L234A,<br>L235A	Musmus IGHG1 CH2<br>1.6-3 (231-239)<br>APNLLGGPS ><br>APEAAGGPS	**ADCC reduction**.<br>Reduces FcγR binding [188].	**CDC reduction**.<br>Reduces C1q binding [188].	
**647**	**Musmus**	**2-G2Bv1**	**CH2**<br>**L1.2**	CH2<br>E1.2>L (235)	E235L	Musmus IGHG2B CH2<br>1.6-3 (231-239)<br>APNLEGGPS ><br>APNLLGGPS	**ADCC enhancement**.<br>Increases FcγRI affinity [189].		
**648**	**Musmus**	**5-G2Bv2**	**CH2**<br>**A101**	CH2<br>E101>A (318)	E318A	Musmus IGHG2B CH2<br>100-110 (317-327)<br>KEFKCKVNNKD ><br>KAFKCKVNNKD	**CDC reduction**.<br>Reduces C1q binding [190].	Musmus IGHG2B CH2 E101, K103 and K105 form a common core in the interaction of IgG and C1q [190].	

**649**	**Musmus**	**5-G2Bv3**	**CH2** A103	CH2 K103>A (320)	K320A	Musmus IGHG2B CH2 100-110 (317-327) KEFKCKVNNKD > KEFACKVNNKD	**CDC reduction**. Reduces C1q binding [190].	Musmus IGHG2B CH2 E101, K103 and K105 form a common core in the interaction of IgG and C1q [190].	
**650**	**Musmus**	**5-G2Bv4**	**CH2** A105	CH2 K105>A (322)	K322A	Musmus IGHG2B CH2 100-110 (317-327) KEFKCKVNNKD > KEFKCAVNNKD	**CDC reduction**. Reduces C1q binding [190].	Musmus IGHG2B CH2 E101, K103 and K105 form a common core in the interaction of IgG and C1q [190].	
**651**	**any chain of any species**	**11-scFv-v1**				C49 (VH)-C120 (VL) (found in antibody intra-H chain, or intra-L chain *galvokimig 12378 (129,91)*)	Engineered additional disulfide bond to stabilize the scFv.		13 *emerfetamab 11239 (123,85)...*
**652**	**any chain of any species**	**11-scFv-v2**				C120 (VL)-C49 (VH) (found in antibody intra-H chain)	Engineered additional disulfide bond to stabilize the scFv.		8 *givastomig 12650 (129,91)...*
**653**	**any chain of any species**	**11-scFv-v3**				C48 (VL)-C10 linker, C13 linker-C120 (VH)	Engineered additional disulfide bonds to stabilize the scFv.		1 *nelvutamig 13774 (133,95).*
**654**	**any chain of any species**	**11-scFv-v4**				C48 (VH)-C10 linker, C13 linker-C120 (VL)	Engineered additional disulfide bonds to stabilize the scFv.		0
**690**	**Homsap**	**9-Jchain-v1**	**JCHAIN** A103	JCHAIN Y103>A (103)	Y103A	JCHAIN 97-110 (97-110) SATETCYTYDRNKC > SATETCATYDRNKC	**Half-life decrease.** Reduces IgM binding to IgM receptors. Faster clearance.		1 *aplitabart 12990 (130,92)*
