# Supplementary material for: IMGT-NC Engineered Variants of INN Therapeutic IG or Antibodies and Related IgSF Proteins (TR, FPIA and CPCA): Bridging Sequences, Structures and Functions for AI
Source: Biomolecules. 2026 Sep 1;16(9):1264. doi: 10.3390/biom16091264 (PMC13604298; doi:10.3390/biom16091264)
Supplement: Supplementary file 1 [file biomolecules-16-01264-s001.zip › Table S2_biomolecules-44933254_Lefranc_260826.pdf]

Table S2. The 228 IMGT-NC engineered variants assigned to INN, up to lists R95 and PL134, included.

The first (one or two) INN name(s), INN number(s) and, between parentheses, proposed (PL) and recommended (RL) list numbers, are displayed in green italics. (Author and Editor Marie-Paule Lefranc, marie-paule.lefranc@umontpellier.fr, 14/07/2026). (With permission from M-P. Lefranc and G. Lefranc, LIGM, Founders and Authors of IMGT®, the international ImMunoGeneTics information system®, https://www.imgt.org).

ID[a]	IMGT-NC engineered variant name	Nb[b]	INN name and INN number (proposed list, recommended list)
**002**	**1-G1v1-2-3**	**1**	*levilimab 10905 (120,82).*
**007**	**6-G1v3-1**	**5**	*vedolizumab 9093 (100,62), ozanezumab 9703 (108,70),*<br>*plozalizumab 10124 (113,75), refanezumab 10174 (114,76),*<br>*plozalizumab plevistinag 13431 (132,94).*
**008**	**6-G1v4**	**1**	*lifonebart 13851 (134,).*
**009**	**6-G1v4-2**	**1**	*tixestobart 13358 (132,94).*
**010**	**6-G1v4-66**	**1**	*abelacimab 10881 (119,81).*
**013**	**2-G1v6**	**7**	*gotistobart 12383 (128,90), amulirafusp alfa* (IG) *12963 (130/131,92),*<br>*tazlestobart 12964 (130,92), vislarafusp alfa* (IG) *12965 (130/131,92),*<br>*palverafusp alfa* (IG) *12967 (130,92), suvonstobart 13347 (132,94),*<br>*moflerafusp alfa* (IG) *13348 (132,94).*
**014**	**2,7-G1v7**	**6**	*talacotuzumab 10508 (117,79), tafasitamab 10835 (119,81),*<br>*lacutamab 10924 (120,82), porustobart 12381 (127,89),*<br>*vilastobart 12761 (129,91), tecotabart 13031 (130,92).*
**015**	**10-G1v7-1**<br>*chimerisotype G1[8aaG2]*	**2**	*talacotuzumab 10508 (117,79), tafasitamab 10835 (119,81).*
**016**	**2,5,7-G1v8**	**1**	*botensilimab 11659 (124,86).*
**018**	**2-G1v9-1**	**2**	*margetuximab 9799 (109,71), enoblituzumab 10165 (114/116, 76/77).*
**021**	**3-G1v12**	**1**	*elipovimab (120,82).*
**022**	**3-G1v13**	**2**	*ipsoprubart 13680 (133,95), emzotamig 13697 (133,95).*
**023**	**6-G1v14**	**65**	*teplizumab 8869 (87/97,59) 'hOKT3', perakizumab 9648 (107,69),*<br>*bimagrumab 9711 (108/110,70), lodelcizumab 9733 (108,70),*<br>*tesidolumab 10051 (112,74), risankizumab 10128 (113,75),*<br>*elezanumab 10344 (115,77), lutikizumab 10347 (115,77),*<br>*duvortuxizumab 10506 (116,78), ravagalimab 10750 (118,80),*<br>*imaprelimab 10753 (118,80), spesolimab 10845 (119,81),*<br>*dilpacimab 10863 (119,81), prolgolimab 10904 (119,81),*<br>*budigalimab 10908 (119,81), cendakimab 10990 (120,82),*<br>*batoclimab 11064 (121,83), manelimab 11133 (121,83),*<br>*mirzotamab 11146 (121,83), mirzotamab clezutoclax 11147 (121,83), obrindatamab 11181 (123,85),*<br>*domvanalimab 11559 (124,86),*<br>*simridarlimab 11730 (125,87), ivonescimab 11809 (125,87),*<br>*etesevimab 11873 (124,85), upanovimab 11966 (125,87),*<br>*rimteravimab 12047 (125,87), dresbuxelimab 12115 (125,87),*<br>*plutavimab 12159 (126,88), prafnosbart 12177 (127,89),*<br>*tividenofusp alfa* (CPCA) *12197 (128,90), empasiprubart 12277 (127,89/95),*<br>*sotiburafusp alfa* (IG) *12407 (128,90), eurestobart 12434 (128,90),*<br>*fanastomig 12694 (129,91), luvagrobart 12725 (129,91),*<br>*opelkibart 12810 (129,91), opelkibart elmanitin 12811 (129,91),*<br>*obrixtamig 12889 (130,92), rinatabart 13029 (130,92),*<br>*rinatabart sesutecan 13030 (130,92), efimosfermin alfa* (CPCA) *13045 (130,92), larnefpendekin alfa* (FPIA) *13051 (131,93), amostomig 13052 (130,92),*<br>*greziprubart 13120 (131,93), cizutamig 13166 (131,93),*

			imeroprubart 13167 (131,93), imneskibart 13202 (131,93), clesitamig 13237 (131,93), recibokibart 13255 (131,93), betinukibart 13289 (131,93), pumitamig 13367 (132,94), etuptamig 13402 (132,94), adimanebart 13433 (132,94), arumakimig 13493 (132,94), lumivatamig 13593 (133,95), zumilokibart 13638 (133,95) fetrastobart vedotin 13768 (133,95), fetrastobart 13769 (133,95), selcodebart 13796 (133,95), afivastobart 13876 (134,), elfetabart drozuntecan 13946 (134,), elfetabart 13947 (134,), corveldebart 13975 (134,), torutamig 13997 (134,).
**024**	**6-G1v14-1**	**23**	*domagrozumab 10286 (114,76), marstacimab 10789 (119,81), penpulimab 11497 (123,85), cadonilimab 11581 (124,86), ponsegromab 11605 (124,86), tarcocimab 11770 (125,87), tarcocimab tedromer 11774 (126/129/130,88/90), tagitanlimab 11843 (125,87), evorpacept* (FPIA) *12029 (126,88), dazukibart 12518 (128,90), ledostomig 12726 (132,), sifarobocept* (FPIA) *12787 (129,91), etakafusp alfa* (IG) *12919 (130/131,92), cugrastomig 12924 (130,92), mozistobart zoratolimod 12947 (130,92) mozistobart 12946 (130,92), mipletamig 12998 (130,92), afimkibart 13205 (131,93), tabirafusp alfa tedromer* (IG) *13242 (131/133,93/94), tabirafusp alfa* (IG) *13243 (131/133,93/94), tixentamig 13477 (132,94), ompekimig 13515 (132,94), tilrekimig 13516 (132,94).*
**025**	**6,5-G1v14-1-20**	**5**	*emfizatamab 12023 (126,88), danvilostomig 12198 (127,89), umizortamig 12292 (127,89), nebratamig 12499 (128,90), mipletamig 12998 (130,92).*
**027**	**6-G1v14-4**	**2**	*tulisokibart 12368 (127,89), opamtistomig 13330 (132,94).*
**029**	**6-G1v14-48**	**4**	*delpasibart 12399 (127,89), delpasibart etedesiran 12400 (127,89), delpasibart zotadirsen 13250 (131,93), delpasibart braxlosiran 13251 (131,93).*
**030**	**6-G1v14-49**	**16**	*cergutuzumab amunaleukin 10080 (113,75), faricimab 10563 (118,80), cibisatamab 10636 (118,80), simlukafusp alfa* (IG) *11116 (121,83), glofitamab 11145 (121,83), alnuctamab 11457 (123,85), melredableukin alfa* (IG) *11723 (125/126,87), englumafusp alfa* (IG) *11793 (125/127,89), masavibart 12234 (126,88), nepuvibart 12235 (126,88), eciskafusp alfa* (IG) *12317 (127,89), tobemstomig 12318 (127,89), lomvastomig 12319 (127,89), forimtamig 12346 (127,89), simaravibart 12423 (127,89), zareprumig 13958 (134,).*
**032**	**6-G1v14-67**	**7**	*reozalimab 11904 (126,88), obertamig 12551 (128,90), pasritamig 13153 (131,93), ramantamig 13340 (132,94), anafiltamig 13438 (133,95), nelvutamig 13774 (133,95), remigromig 13973 (134,).*
**040**	**5-G1v20**	**8**	*nivatrotamab 11591 (124,86), anzurstobart 12363 (127,89), mipletamig 12998 (130,92), donitabart 13053 (130,92), avitotamig 13055 (130,92), daretabart 13434 (132,94), gamsitabart 13908 (134,), gamsitabart tocentecan 13909 (134,).*
**041**	**9-G1v21**	**34**	*netakimab 10387 (118,80), suvratoxumab 10441 (116,78), nirsevimab 10780 (119,81), levilimab 10905 (120,82), ziltivekimab 11143 (121,83), avizakimab 11148 (121,83), depemokimab 11449 (123,85), recaticimab 11502 (123,85), tixagevimab 11776 (124,85), cilgavimab 11777 (124,85), barzolvolimab 11842 (125,87), clesrovimab 11857 (126,88), amubarvimab 11988 (125,87), romlusevimab 11992 (125,87), izeltabart (12302 (127,89), izeltabart tapatansine 12303 (127,89), gotistobart 12383 (128,90), picankibart 12419 (128,90),*

			sipavibart 12778 (129,91), ozureprubart 12977 (130,92), navenibart 12991 (130,92), donitabart 13053 (130,92), efitazanamivir alfa (CPCA) *13066 (132,), amtabafusp alfa* (IG) *13207 (131,93), vebanvibart 13272 (130,92), trovostobart 13453 (132,94), lasrekibart 13500 (132,94), elegrobart 13528 (132,94), glarivibart 13605 (133,95), zumilokibart 13638 (133,95), felcorekibart 13642 (133,95), solaprubart 13933 (134,), ombetoxabart 13952 (134,), torutamig 13997 (134,).*
**043**	**9-G1v22-1**	**1**	*efgartigimod alfa* (IG) *10455 (116,78).*
**045**	**9-G1v24**	**21**	*elipovimab 10988 (120,82), vudalimab 11464 (123,85), bavunalimab 11465 (123/*125*,87), izuralimab 11466 (123,85), sotrovimab 11766 (124,85), teropavimab 11878 (125,87), zinlirvimab 11879 (126,88), beludavimab 11961 (125,87), masavibart 12234 (126,88), nepuvibart 12235 (126,88), tobevibart 12316 (127,89), ogalvibart 12361 (126,88), crexavibart 12362 (126,88), simaravibart 12423 (127,89), efbalropendekin alfa* (FPIA) *12475 (128/*130*,90), sonavibart 13036 (130,92), lotivibart 13158 (131,93), ompekimig 13515 (132,94), tilrekimig 13516 (132,94), retavibart 13354 (132,94), lifonebart 13851 (134,).*
**046**	**9-G1v24-1**	**1**	*gimsilumab 10534 (117,79).*
**047**	**7-G1v25**	**1**	*obexilimab 10898 (119,81).*
**049**	**16-G1v27**	**6**	*vadastuximab talirine (113,75), vadastuximab 10242 (114,76), rolinsatamab talirine (119,81), rolinsatamab 10874 (119,81), serclutamab talirine 10959 (120,82), serclutamab 10960 (120,82).*
**050**	**16-G1v27-1**	**2**	*iladatuzumab 10646 (117,!), iladatuzumab vedotin 10647 (117,!).*
**051**	**16-G1v28**	**1**	*idactamab 11348 (123,85).*
**052**	**8-G1v29**	**33**	*otelixizumab 8864 (98,60), clazakizumab 9599 (107,69), atezolizumab 9814 (112,74), eptinezumab 10308 (115,77), iscalimab 10707 (118,90), nipocalimab 11215 (122,84), opucolimab 11310 (122,84), nivatrotamab 11591 (124,86), ulildlimab 11597 (124,86), sudubrilimab 11649 (124,86), rulonilimab 11710 (125,87), dalutrafusp alfa* (IG) *11814 (125,87), betifisolimab 11926 (126,88), evorpacept* (FPIA) *12029 (126,88), epacmarstobart 12164 (127,89), tuparstobart 12389 (127,89), verzistobart 12390 (128,90), givastomig 12650 (129,91), uprevstobart 12670 (129,91), ragistomig 12707 (129,91), xirestomig 12746 (129,91), efercoleukin alfa* (FPIA) *12762 (129,91), rezorstobart 12766 (129,91), lumistobart 13012 (130,92), balekafusp alfa* (IG) *13040 (132/133/*134*,95), avitotamig 13055 (130,92), invikafusp alfa* (IG) *13065 (130/*131*,92), ruxoprubart 13213 (131,93), efarindodekin alfa* (FPIA) *13222 (131,93), clesitamig 13237 (131,93), bocunebart 13356 (132,94), asedebart 13606 (133,95), olevaprubart 13949 (134,).*
**054**	**8-G1v30**	**7**	*mosunetuzumab 10621 (117,79), efavaleukin alfa* (FPIA) *10762 (118,80), zelminemab 11091 (121,83), cevostamab 11258 (122,84), runimotamab 11496 (124,86), fazpilodemab 12017 (126,88), linclatamig 13026 (130,92).*
**055**	**8-G1v30-1**	**1**	*semzuvolimab 11685 (126,88).*
**057**	**14-G1v31-1** (hole)	**1**	*zeclantamig 13865 (134,).*
**058**	**14-G1v32** (knob)	**60**	*onartuzumab 9368 (104,66), vanucizumab 9950 (111,73), cergutuzumab amunaleukin 10080 (113,75), duvortuxizumab 10506 (116,78), faricimab 10563 (118,80), mosunetuzumab 10621 (117,79),*

			cibisatamab 10636 (118,80), simlukafusp alfa (IG) *11116 (121,83),*<br>*glofitamab 11145 (121,83), obrindatamab 11181 (123,85),*<br>*cevostamab 11258 (122,84), alnuctamab 11457 (123,85),*<br>*runimotamab 11496 (124,86), anbenitamab 11611 (124,86),*<br>*simridarlimab 11730 (125,87), goflikicept* (FPIA) *11764 (124,85),*<br>*englumafusp alfa* (IG) *11793 (125/127,89), fazpilodemab 12017 (126,88),*<br>*trontinemab 12120 (126,88), volrustomig 12168 (127,89),*<br>*tividenofusp alfa* (CPCA) *12197 (128,90), eciskafusp alfa* (IG) *12317 (127,89),*<br>*tobemstomig 12318 (127,89), lomvastomig 12319 (127,89),*<br>*forimtamig 12346 (127,89), rilvegostomig 12387(127,89),*<br>*sabestomig 12457 (128,90), silevimig 12660 (129,91),*<br>*efercoleukin alfa* (FPIA) *12762 (129,91), obrixtamig 12889 (130,92),*<br>*etakafusp alfa* (IG) *12919 (130/131,92), opugotamig 12941 (130,92),*<br>*opugotamig olatansine 12957 (130,92), tilatamig 13020 (130,92),*<br>*tilatamig samrotecan 13021 (130,92), linclatamig 13026 (130,92),*<br>*amostomig 13052 (130,92), invikafusp alfa* (IG) *13065 (130/131,92),*<br>*velinotamig 13145 (131,93), amtabafusp alfa* (IG) *13207 (131,93),*<br>*efarindodekin alfa* (FPIA) *13222 (131,93), clesitamig 13237 (131,93),*<br>*spevatamig 13260 (131,93), peluntamig 13261 (131,93),*<br>*ramantamig 13340 (132,94), etuptamig 13402 (132,94),*<br>*efranarelaxin alfa* (CPCA) *13420 (132/133,94), anafiltamig 13438 (133,95),*<br>*anbenitamab repodatecan 13457 (132,94), tixentamig 13477 (132,94),*<br>*arumakimig 13493 (132,94), lumivatamig 13593 (133,95),*<br>*tepilukimig 13604 (133,95), emzotamig 13697 (133,95),*<br>*nelvutamig 13774 (133,95), zeclantamig 13865 (134,),*<br>*rolditamig deuderuxtecan 13866 (134,), zareprumig 13958 (134,),*<br>*torutamig 13997 (134,), rolditamig 14031 (134,).*
**059**	**14-G1v32-1** (knob)	**1**	*remigromig 13973 (134,).*
**060**	**14-G1v33** (hole)	**59**	*onartuzumab 9368 (104,66), vanucizumab 9950 (111,73),*<br>*cergutuzumab amunaleukin 10080 (113,75), duvortuxizumab 10506 (116,78), faricimab 10563 (118,80), mosunetuzumab 10621 (117,79),*<br>*cibisatamab 10636 (118,80), simlukafusp alfa* (IG) *11116 (121,83),*<br>*glofitamab 11145 (121,83), obrindatamab 11181 (123,85),*<br>*cevostamab 11258 (122,84), alnuctamab 11457 (123,85),*<br>*runimotamab 11496 (124,86), anbenitamab 11611 (124,86),*<br>*simridarlimab 11730 (125,87), goflikicept* (FPIA*) 11764 (124,85),*<br>*englumafusp alfa* (IG) *11793 (125/127,89), fazpilodemab 12017 (126,88),*<br>*trontinemab 12120 (126,88), volrustomig 12168 (127,89),*<br>*tividenofusp alfa* (CPCA) *12197 (128,90), eciskafusp alfa* (IG) *12317 (127,89),*<br>*tobemstomig 12318 (127,89), lomvastomig 12319 (127,89),*<br>*forimtamig 12346 (127,89), rilvegostomig 12387(127,89),*<br>*sabestomig 12457 (128,90), silevimig 12660 (129,91),*<br>*efercoleukin alfa* (FPIA) *12762 (129,91), obrixtamig 12889 (130,92),*<br>*etakafusp alfa* (IG) *12919 (130/131,92), opugotamig 12941 (130,92),*<br>*opugotamig olatansine 12957 (130,92), tilatamig 13020 (130,92),*<br>*tilatamig samrotecan 13021 (130,92), linclatamig 13026 (130,92),*<br>*amostomig 13052 (130,92), invikafusp alfa* (IG) *13065 (130/131,92),*<br>*velinotamig 13145 (131,93), amtabafusp alfa* (IG) *13207 (131,93),*<br>*efarindodekin alfa* (FPIA) *13222 (131,93), clesitamig 13237 (131,93),*<br>*spevatamig 13260 (131,93), peluntamig 13261 (131,93),*<br>*ramantamig 13340 (132,94), etuptamig 13402 (132,94),*<br>*efranarelaxin alfa* (CPCA) *13420 (132/133,94), anafiltamig 13438 (133,95),*<br>*anbenitamab repodatecan 13457 (132,94), tixentamig 13477 (132,94),*

			arumakimig 13493 (132,94), lumivatamig 13593 (133,95), tepilukimig 13604 (133,95), emzotamig 13697 (133,95), nelvutamig 13774 (133,95), rolditamig deuderuxtecan 13866 (134,), zareprumig 13958 (134,), torutamig 13997 (134,), rolditamig 14031 (134).
**061**	**14-G1v33-1** (hole)	**1**	*remigromig 13973 (134,).*
**062**	**10,13-G1v34**	**4**	*benufatamab 11063 (121,83), tilogatamab 11267 (122,84)***,** *ivicentamab 11702 (125,87), erzotabart 12562 (128,90).*
**063**	**4-G1v35**	**2**	*sotigalimab 11472 (123,85), exlinkibart 12376 (127,89).*
**064**	**8-G1v36**	**4**	*mupadolimab 11944 (125,87), briquilimab 12124 (126,88), temtokibart 12231 (128,90), trevotamig 13209 (131,93).*
**065**	**8-G1v36-1**	**1**	*eftozanermin alfa* (FPIA) *10838 (119,81).*
**066**	**15-G1v37**	**46**	*placulumab 9567(107,69), ipafricept* (FPIA) *9810 (109,71), otlertuzumab 9832 (110,72), rivabazumab pegol* (Fab) *10144 (113,75), rivabazumab* (Fab) *10197 (114,76), ontorpacept* (FPIA) *10927 (122,84), envafolimab 10930 (120,82), tamrintamab pamozirine 10961 (120,82), tamrintamab 10962 (120,82), zanidatamab 10997 (121,83), plamotamab 11020 (120,82), vibecotamab 11021 (120,82), tidutamab 11022 (120,82), acazicolcept* (FPIA) *11385 (124,86), zanidatamab zovodotin 11433 (123,85), vudalimab 11464 (123,85), bavunalimab 11465 (123,87), izuralimab 11466 (123,85), erfonrilimab 11617 (124,86), crefmirlimab 11678 (126,88), davoceticept* (FPIA) *11827 (125,87), tuvonralimab 11841 (125,87), zirconium (89Zr) crefmirlimab berdoxam 12138 (127,89), povetacicept* (FPIA) *12264 (127,89), efbalropendekin alfa* (FPIA) *12475 (128/*130*,90), obertamig 12551 (128,90), sifarobocept* (FPIA) *12787 (129,91), canvircept* (CPCA) *12882 (129,91), opugotamig 12941 (130,92), opugotamig olatansine 12957 (130,92), mipletamig 12998 (130,92), lofacimig 13117 (131,93), pasritamig 13153 (131,93), spevatamig 13260 (131,93), ramantamig 13340 (132,94), velaprumig 13342 (132,94), anafiltamig 13438 (133,95), tixentamig 13477 (132,94), turmetabart adizutecan 13671 (133,95), turmetabart 13672 (133,95), nelvutamig 13774 (133,95,), alveltamig 13944 (134,), remigromig 13973 (134,), rolditamig deuderuxtecan 13866 (134,), rolditamig 14031 (134,).* Note: variant included in 15-G2v37-1 *G2[hG1] abituzumab 9509 (109,71)*
**067.1**	**15-G1v37-1**	**1**	*tuvonralimab 11841 (125,87).*
**067.2**	**15-G1v37-2**	**1**	*efgivanermin alfa* (FP1A) *10695 (120,82).*
**068**	**6-G1v38**	**1**	*paradiprubart 12233 (126,88)*
**069**	**6-G1v39**	**22**	*anifrolumab 9800 (109,71), durvalumab 10010 (112,74), oleclumab 10545 (116,78), tixagevimab 11776 (124,85), cilgavimab 11777 (124,85), golocdacimab 12003 (126,88), camoteskimab 12062 (126,88), volrustomig 12168 (127,89), rilvegostomig 12387 (127,89), sabestomig 12457 (128,90), lixudebart 12552 (128,90), sipavibart 12778 (129,91), indenebart 13006 (130,92), ansipastobart 13011 (130,92), tilatamig 13020 (130,92), tilatamig samrotecan 13021 (130,92), telikibart 13144 (131,93), velinotamig 13145 (131,93), adezkibart 13365 (132,94), efranarelaxin alfa* (CPCA) *13420 (132/*133*,), tepilukimig 13604 (133,95), alveltamig 13944 (134,).*
**070**	**6-G1v40**	**3**	*amtabafusp alfa* (IG) *13207 (131,93), latozinemab 11584 (124,86)***,** *nivisnebart 13157 (131,93).*
**072**	**6-G1v41-66**	**4**	*epcoritamab 11078 (121,83), acasunlimab 11565 (124,86),*

			tecaginlimab 11729 (125,87), zubotamig 13007 (130,92).
**073**	**9-G1v42**	**3**	*elezanumab 10344 (115,77), ravagalimab 10750 (118,80), rulonilimab 11710 (125,87).*
**074**	**6-G1v43**	**5**	*olamkicept* (FPIA) *10311 (116,78), acazicolcept* (FPIA) *11385 (124,86), davoceticept* (FPIA) *11287 (125,87), bempikibart 12242 (127,89), povetacicept* (FPIA) *12264 (127,89).*
**075**	**6-G1v43-1**	**3**	*torapsel* (CPCA) *8337 (91,53), anrukinzumab 8942 (98,60), dalantercept* (FPIA) *9427 (105,67).*
**076**	**6-G1v43-2**	**1**	*foralumab 9309 (103,65).*
**078**	**6-G1v43-60**	**6**	*atacicept* (FPIA) *8669 (95,57), telitacicept* (FPIA) *10932 (120,82), avdoralimab 11081 (121,83), lodapolimab 11185 (121,83), renvistobart 12465 (128,90), izastobart 12721 (129,91).*
**079**	**16-G1v44**	**4**	*pivekimab 11693 (125,87), pivekimab sunirine 11695 (125,87), izeltabart 12302 (127,89), izeltabart tapatansine 12303 (127,89).*
**080**	**3-G1v45**	**2**	*beludavimab 11961 (125,87), tobevibart12316 (127,89).*
**081**	**9-G1v46**	**1**	*empasiprubart 12277 (127,89).*
**082**	**1-G1v47**	**2**	*exlinkibart 12376 (127,89), dalnicastobart 13277 (127,89).*
**085**	**1-G1v50**	**4**	*precemtabart tocentecan 13008 (130,92), precemtabart 13071 (130,92), gamsitabart 13908 (134,), gamsitabart tocentecan 13909 (134,).*
**087**	**6-G1v50-1-4**	**2**	*garivulimab 11451 (123,85), surzebiclimab 11453 (124,86).*
**089**	**6-G1v50-51**	**9**	*plamotamab 11020 (120,82), vibecotamab 11021 (120,82), tidutamab 11022 (120,82), vudalimab 11464 (123,85), bavunalimab 11465 (123/125,87), izuralimab 11466 (123,85), xaluritamig 12082 (127,89), efbalropendekin alfa* (FPIA) *12475 (128/*130*,90), emaretamig 13773 (133,95).*
**090**	**6-G1v50-79**	**1**	*timcevibart 12929 (129,91).*
**094**	**6-G1v53-1**	**1**	*barzolvolimab 11842 (125,87).*
**095**	**11-G1v54**	**1**	*emzotamig 13697 (133,95).*
**097**	**11,8-G1v54-30**	**14**	*emerfetamab 11239 (123,85), pavurutamab 11290 (123,85), zeluvalimab 11417 (124,86), tarlatamab 11420 (123,85), acapatamab 11421 (123/124,85), gresonitamab 11798 (125,87), vepsitamab 11822 (125,87), emirodatamab 12028 (126,88), xaluritamig 12082 (127,89), inezetamab 12088 (126,88), latikafusp* (IG) *12098 (126,88), iluzanebart 12479 (128,90), maridebart 12601 (128,90), maridebart cafraglutide (128,90).*
**099**	**16-G1v55**	**4**	*tarcocimab 11770 (129,90), tarcocimab tedromer 11774 (126/129/*130*,88/*90*) tabirafusp alfa tedromer* (IG) *13242 (131/*133*,93/*94*), tabirafusp alfa* (IG) *13243 (131/*133*,93/*94*).*
**100**	**16-G1v56**	**4**	*luveltamab tazevibulin 12040 (126,88), luveltamab tazide 12041 (126,88), ispectamab debotansine 12079 (126,88), ispectamab tazide 12139 (127,89).*
**101**	**16-G1v56-1**	**2**	*anvatabart pactil 12271 (127,89), anvatabart opadotin 12272 (127,89).*
**102**	**16-G1v56-2**	**2**	*bezetabart 12653 (129,91), bezetabart debotansine 12652 (129,91).*
**103**	**17-G1v57**	**9**	*glofitamab 11145_H (121,83), alnuctamab 11457_H (123,85), englumafusp alfa* (IG) *11793_N (125/*127*,89), trontinemab 12120_H,M (126/*127*,88), tobemstomig 12318_M (127/*129*,89/*90*), lomvastomig 12319_M (127,89), forimtamig 12346 (127,89), clesitamig 13237_ H,M (131,93), zareprumig 13958_M (134,).* Idem[d] 17-KCv57 (9).
**106**	**17-G1v57-3**	**1**	*tuvonralimab 11841 (125,87).*
**107**	**17-G1v58**	**5**	*volrustomig 12168 (127,89), rilvegostomig 12837 (127,89), sabestomig 12457 (128,90), tilatamig 13020 (130,92), tilatamig samrotecan 13021 (130/132, 92/93).*
**111**	**6-G1v59-1**	**2**	*tepoditamab 10766 (118,80), besufetamig 13363 (132,94).*

**112**	**6-G1v59-2**	**1**	*crovalimab 10882 (119,81).*
**113**	**6-G1v60**	**4**	*precemtabart 13071 (130,92), precemtabart tocentecan 13008 (130,92),*<br>*gamsitabart 13908 (134,), gamsitabart tocentecan 13909 (134,).*
**114**	**6-G1v60-1**	**1**	*crovalimab 10882 (119,81).*
**117**	**6-G1v63**	**4**	*abatacept* (FPIA) *8495 (91,53), belatacept* (FPIA) *8627 (9359),*<br>*letolizumab 10436 (116,78), tegoprubart 12545 (128,90).*
**118**	**6,7-G1v63-2**	**1**	*emugrobart 13238 (131,93).*
**120**	**6-G1v64**	**2**	*maridebart 12601 (128,90), maridebart cafraglutide 12602 (128,90).*
**121**	**6-G1v65**	**2**	*enristomig 12416 (129,91), ozekibart 12417 (128,99).*
**122**	**1-G1v66**	**4**	*benmelstobart 12205 (128,90), zubotamig 13007 (130,92),*<br>*opamtistomig 13330 (132,), lifonebart 13851 (134,).*
**123**	**1-G1v66-80**	**1**	*envafolimab 10930 (120,82)*
**125**	**14-G1v68**	**5**	*zanidatamab 10997 (121,83), zanidatamab zovodotin 11433 (123/*125*,85/*86*),*<br>*reozalimab 11904 (126,88), obertamig 12551 (128,90),*<br>*pasritamig 13153 (131,93).*
**126**	**17-G1v68-1**	**1**	*reozalimab 11904 (126,88).*
**127**	**14-G1v69**	**5**	*zanidatamab 10997 (121,83), zanidatamab zovodotin 11433 (123/*125*,85/*86*),*<br>*reozalimab 11904 (126,88), obertamig 12551 (128,90),*<br>*pasritamig 13153 (131,93).*
**128**	**17-G1v69-1**	**1**	*reozalimab 11904 (126,88).*
**129**	**15-G1v70**	**4**	*abatacept* (FPIA) *8495 (91,53), belatacept* (FPIA) *8627 (93,59),*<br>*letolizumab 10436 (116,78), tegoprubart 12545 (128,90).*
**130**	**14-G1v72**	**5**	*zenocutuzumab 10687_M (117/*118*,79), tepoditamab 10766_H (118,80),*<br>*petosemtamab 11136_H (121,83), pamvatamig 12549_H (129,91),*<br>*besufetamig 13363_H (132,94).*
**131**	**14-G1v72-1**	**1**	*fidasimtamab 11670_M (125,87).*
**132**	**14-G1v72-2**	**2**	*latikafusp* (IG) *12098 (126,88), zeclantamig 13865 (134,).*
**133**	**14-G1v72-3**	**9**	*plamotamab 11020 (120,82), vibecotamab 11021 (120,82),*<br>*tidutamab 11022 (120,82), vudalimab 11464 (123,85),*<br>*bavunalimab 11465 (123,87), izuralimab 11466 (123,85),*<br>*xaluritamig 12082 (127,89), efbalropendekin* (FPIA) *12475 (128/*130*,90),*<br>*emaretamig 13773 (133,95).*
**135**	**14-G1v72-5**	**1**	*aveltamig 13944 (134,).*
**136**	**14-G1v73**	**5**	*zenocutuzumab 10687_H (117/*118*,79), tepoditamab 10766_M (118,80),*<br>*petosemtamab 11136_M (121,83), pamvatamig 12549_M (129,91),*<br>*besufetamig 13363_M (132,94).*
**137**	**14-G1v73-1**	**1**	*fidasimtamab 11670_H (125,87).*
**138**	**14-G1v73-2**	**1**	*latikafusp* (IG) *12098_H (126,88).*
**139**	**10-G1v73-3**	**1**	*emaretamig 13773 (133,95).*
**141**	**14-G1v73-5**	**1**	*zeclantamig 13865 (134,).*
**142**	**14-G1v73-6**	**1**	*aveltamig 13944 (134,).*
**143**	**14-G1v74**	**44**	• 39 with 14-G1v32 :<br>*vanucizumab 9950 (111,73), cergutuzumab amunaleukin 10080 (113,75)*,<br>*faricimab 10563 (118,80), cibisatamab 10636 (118,80),*<br>*simlukafusp alfa* (IG) *11116 (121,83), glofitamab 11145 (121,83),*<br>*alnuctamab 11457 (123,85), simridarlimab 11730 (125,87),*<br>*goflikicept* (FPIA) *11764 (124,85), englumafusp alfa* (IG) *11793 (125/*127*,89),*<br>*trontinemab 12120 (126,88), volrustomig 12168 (127,89),*<br>*eciskafusp alfa* (IG) *12317 (127,89), tobemstomig 12318 (127,89),*<br>*lomvastomig 12319 (127,89), forimtamig 12346 (127,89),*<br>*rilvegostomig 12387(127,89), sabestomig 12457 (128,90),*<br>*silevimig 12660 (129,91), efercoleukin alfa* (FPIA) *12762 (129,91),*

			tilatamig 13020 (130,92), tilatamig samrotecan 13021 (130,92),<br>amostomig 13052 (130,92), invikafusp alfa (IG) 13065 (130/131,92),<br>velinotamig 13145 (131,93), efarindodekin alfa (FPIA) 13222 (131,93),<br>clesitamig 13237 (131,93), spevatamig 13260 (131,93),<br>peluntamig 13261 (131,93), efranarelaxin alfa (CPCA) 13420 (132,94),<br>tixentamig 13477 (132,94), arumakimig 13493 (132,94),<br>lumivatamig 13593 (133,95), tepilukimig 13604 (133,95),<br>emzotamig13697 (133,95), rolditamig deuderuxtecan 13866 (134,),<br>zareprumig 13958 (134,), torutamig 13997 (134,),<br>rolditamig 14031 (134,),<br>• 1 with 14-G1v33 :<br>etakafusp alfa (IG) 12919 (130/131,92),<br>• 3 with 14-G1v91 :<br>ingitamig 12958 (131,93), azerutamig 13130 (131,93),<br>vadalentamig 13892 (134,),<br>• 1 with 14-G1v73-6 :<br>alveltamig 13944 (134,).
**144**	**14-G1v75**	**44**	• 39 with 14-G1v33 :<br>vanucizumab 9950 (111,73), cergutuzumab amunaleukin 10080 (113,75),<br>faricimab 10563 (118,80), cibisatamab 10636 (118,80),<br>simlukafusp alfa (IG) 11116 (121,83), glofitamab 11145 (121,83),<br>alnuctamab 11457 (123,85), simridarlimab 11730 (125,87),<br>goflikicept (FPIA) 11764 (124,85), englumafusp alfa (IG) 11793 (125/127,89),<br>trontinemab 12120 (126,88), volrustomig 12168 (127,89),<br>eciskafusp alfa (IG) 12317 (127,89), tobemstomig 12318 (127,89),<br>lomvastomig 12319 (127,89), forimtamig 12346 (127,89),<br>rilvegostomig 12387(127,89), sabestomig 12457 (128,90),<br>silevimig 12660 (129,91), efercoleukin alfa (FPIA) 12762 (129,91),<br>tilatamig 13020 (130,92), tilatamig samrotecan 13021 (130,92),<br>amostomig 13052 (130,92), invikafusp alfa (IG) 13065 (130/131,92),<br>velinotamig 13145 (131,93), efarindodekin alfa (FPIA) 13222 (131,93),<br>clesitamig 13237 (131,93), spevatamig 13260 (131,93),<br>peluntamig 13261 (131,93), efranarelaxin alfa (CPCA) 13420 (132,94),<br>tixentamig 13477 (132,94), arumakimig 13493 (132,94),<br>lumivatamig 13593 (133,95), tepilukimig 13604 (133,95),<br>emzotamig13697 (133,95), rolditamig deuderuxtecan 13866 (134,),<br>zareprumig 13958 (134,), torutamig 13997 (134,),<br>rolditamig 14031 (134,),<br>• 1 with 14-G1v32 :<br>etakafusp alfa (IG) 12919 (130/131,92),<br>• 3 with 14-G1v90 :<br>ingitamig 12958 (131,93), azerutamig 13130 (131,93),<br>vadalentamig 13892 (134,),<br>• 1 with 14-G1v72-5 :<br>alveltamig 13944 (134,).
**145**	**16-G1v76**	**4**	opelkibart 12810 (129,91), opelkibart elmanitin 12811 (129,91),<br>pamlectabart 13245 (131,93), pamlectabart tismanitin 13244 (131,93).
**146**	**9-G1v77**	**2**	opelkibart 12810 (129,91), opelkibart elmanitin 12811 (129,91).
**147**	**9-G1v77-1**	**1**	faricimab 10563 (118,80).
**148**	**9-G1v77-2**	**1**	tuvonralimab 11841 (125,87).
**149**	**9-G1v78**	**6**	crovalimab 10882 (119,81), adintrevimab 12026 (125,87),<br>turenkibart 12621 (131,93), pemivibart 13017 (129,91),<br>clesitamig 13237 (131,93), ibramvibart 13806 (132,94).

150	9-G1v78-1	1	*emugrobart 13238 (131,93).*
152	9-G1v78-3	1	*bapineuzumab 8624 (93,55).*
153	9-G1v78-4	1	*tepsababart 13902 (134,).*
156	16-G1v81	2	*micvotabart pelidotin 13003 (130,92), micvotabart 13004 (130,92).*
157	18-G1v82-1	6	*amivantamab 11030 (121,83), epcoritamab 11078 (121/123/127,83/84/88), acasunlimab 11565 (124,86), ivicentamab 11702 (125,87),*<br>*tecaginlimab 11729 (125,87), zubotamig 13007 (130,92).*
158	18-G1v82-2	6	*amivantamab 11030 (121,83), epcoritamab 11078 (121/123/127,83/84/88), acasunlimab 11565 (124,86), ivicentamab 11702 (125,87),*<br>*tecaginlimab 11729 (125,87), zubotamig 13007 (130,92).*
159	18-G1v82-3	3	*anbenitamab 11611 (124,86), goflikicept* (FPIA) *11764 (124,85),*<br>*anbenitamab repodatecan 13457 (132,94)*
160	18-G1v82-4	3	*anbenitamab 11611 (124,86), goflikicept* (FPIA) *11764 (124,85),*<br>*anbenitamab repodatecan 13457 (132,94)*
161	18-G1v82-5	9	*plamotamab 11020 (120,82), vibecotamab 11021 (120,82),*<br>*tidutamab 11022 (120,82), vudalimab 11464 (123,85),*<br>*bavunalimab 11465 (123/125,87), izuralimab 11466 (123,85),*<br>*xaluritamig 12082 (127,89), efbalropendekin alfa* (FPIA) *12475 (128/*130*,90), emaretamig 13773 (133,95).*
162	18-G1v82-6	9	*plamotamab 11020 (120,82), vibecotamab 11021 (120,82),*<br>*tidutamab 11022 (120,82), vudalimab 11464 (123,85),*<br>*bavunalimab 11465 (123/125,87), izuralimab 11466 (123,85),*<br>*xaluritamig 12082 (127,89), efbalropendekin alfa* (FPIA) *12475 (128/*130*,90), emaretamig 13773 (133,95).*
163	18-G1v82-7	1	*tuvonralimab 11841 (125,87).*
166	10-G1v83	8	*obrixtamig 12889 (130,92), ramantamig 13340 (132,94),*<br>*etuptamig 13402 (132,94), anafiltamig 13438 (133,95),*<br>*emzotamig 13697 (133,95), nelvutamig 13774 (133,95),*<br>*rolditamig deuderuxtecan 13866 (134,), rolditamig 14031 (134,).*
167	10-G1v83-1	4	*duvortuxizumab 10506 (116,78), obrindatamab 11181 (123,85),*<br>*amtabafusp alfa* (IG) *13207 (131,93), lumivatamig (133,95).*
168	17-G1v84	1	*tuvonralimab 11841 (125,87).*
169	17-G1v84-1	1	*linclatamig 13026 (130,92).*
170	17-G1v84-2	1	*linclatamig 13026 (130,92).*
171	7-G1v85	1	*atenastobart 12848 (131,93).*
172	7-G1v86	1	*emugrobart 13238 (131,93).*
173	10-G1v87	1	*emugrobart 13238 (131,93).*
174	10-G1v88	2	*atenastobart 12848 (131,93), emugrobart 13238 (131,93).*
176	10-G1v89	3	*crovalimab 10882 (119,81), clesitamig 13237 (131,93),*<br>*emugrobart 13238 (131,93).*
177	14-G1v90	3	*ingitamig 12958 (131,93), azerutamig 13130 (131,93),*<br>*vadalentamig 13892 (134,).*
178	14-G1v91	3	*ingitamig 12958 (131,93), azerutamig 13130 (131,93),*<br>*vadalentamig 13892 (134,).*
179	17-G1v92-1	1	*spevatamig 13260_H (131,93).*
180	17-G1v92-2	1	*spevatamig 13260_M (131,93).*
181	17-G1v93	1	*spevatamig 13260_H (131,93).*
182	14-G1v94	1	*clesitamig 13237_H,_M (131,93).*
183	14-G1v95	2	*velinotamig 13145_M (131,93), tepilukimig 13604 (133,95).*
184	14-G1v96	1	*velinotamig 13145_H,M (131,93).*
185	14-G1v96-1	5	*telisotuzumab vedotin 10365 (115,77), telisotuzumab 10366 (115,77),*<br>*telisotuzumab adizutecan 13001 (130,92), zevontabart 13956 (134,),*

			zevontabart vedotin 13957 (134,).
**187**	**10,13-G1v98**	**1**	*tixestobart 13358 (132,94).*
**189**	**14-G1v99-1**	**2**	*ompekimig 13515_H (132,94), tilrekimig 13516_H (132,94).*
**190**	**14-G1v99-2**	**2**	*ompekimig 13515_M (132,94), tilrekimig 13516_M (132,94).*
**193**	**17-G1v100-1**	**1**	*velaprumig 13342_H (132,94).*
**195**	**1,5-G1v101-20**	**1**	*frexalimab 11963_H (126,88).*
**196**	**2-G1v102**	**1**	*ensituximab 9300_H (103,65).*
**197**	**2-G1v103**	**1**	*ocaratuzumab 9590_H (107,69).*
**198**	**3-G1v104**	**1**	*opicinumab 10090 (113,75).*
**700**	**7-G1v105**	**1**	*dalantercept* (FPIA) *9427 (105,67).*
**701**	**7-G1v106**	**1**	*giloralimab 11241 (122,84).*
**703**	**5-G1v108**	**4**	*torapsel* (CPCA) *8337 (91,53), sotatercept* (FPIA) *9188 (102/*104*,64/*65*),* *dalantercept* (FPIA) *9427 (105,67), ramatercept* (FPIA) *9606 (108,70).*
**707**	**6-G1v112** *chimerisotype G1[12aaG4]*	**1**	*cabotamig 12632 (129,91).*
**711**	**10-G1v115**	**1**	*tividenofusp alfa 12197 (128,90).*
**795*[e]**	**2,7-G1v7, 10-G1v7-1** *chimerisotype G1[8aaG2]*	(2)	*talacotuzumab 10508 (117,79), tafasitamab 10835 (119,81),*
**796***	**14-G1v90, 14-G1v75**	(3)	Idem 14-G1v90.
**797***	**14-G1v91, 14-G1v74**	(3)	Idem 14-G1v91.
**799***	**6-G1v63, 15-G1v70**	(4)	Idem 6-G1v63 (4), idem 6-G1v70 (4).
**203**	**6-G2v3**	**2**	*denrikibart 13177 (131,93), melrilimab 11351 (123,85).*
**204**	**6-G2v3-1**	**2**	*visilizumab 8054 (84,46), quetmolimab 11040 (120,82).*
**209**	**9-G2v8-1**	**1**	*vamikibart 12560 (128,90).*
**210**	**5-G2v20**	**2**	*crizanlizumab 10316 (115,77), cepeprubart 12368 (129,91).*
**211**	**9-G2v21**	**1**	*omoprubart 12638 (129,91)* with 6-G2v94 (chimerisotype *G2[11aaG4]*).
**212**	**9-G2v24**	**1**	*ravulizumab 10659 (117,79)* with 6-G2v94 (chimerisotype *G2[11aaG4]*).
**214**	**8,10-G2v36-7**	**1**	*abituzumab 9509 (109,71)* with 15-G2v37-1 (chimerisotype *G2[hG1S5]*).
**215**	**15-G2v37**	**3**	*nemolizumab 10064 (112,74), satralizumab 10065 (114/116,77/78),* *efsubaglutide alfa* (CPCA) *12853.*
**216**	**15-G2v37-1** *chimerisotype G2[hG1S5]*	**1**	*abituzumab 9509 (109,71)* with 8,10-G2v36-7.
**219**	**6-G2v60**	**7**	*tanezumab 8941 (99,61), ponezumab 9322 (104,66),* *bococizumab 9840 (110,72), ralpancizumab 9841 (110,72),* *fremanezumab 10299 (115,77), elranatamab 11838 (125,87),* *efsubaglutide alfa* (CPCA) *12853 (131,93).*
**221**	**6-G2v60-66**	**1**	*elranatamab 11838 (125,87).*
**223**	**14-G2v72-1**	**1**	*elranatamab 11838 (125,87).*
**224**	**14-G2v72-2**	**1**	*navicixizumab 10220 (114,76).*
**225**	**14-G2v73-1**	**1**	*elranatamab 11838 (125,87).*
**226**	**14-G2v73-2**	**1**	*navicixizumab 10220 (114,76).*
**227**	**9-G2v78-2**	**1**	*satralizumab 10065 (114/116,77/78).*
**228**	**6-G2v94** *chimerisotype G2[11aaG4]*	**6**	*eculizumab 8231 (87,49), samalizumab 9307 (103,65),* *olendalizumab 10037 (114/*116*,78), ravulizumab 10659 (117,79)*, *omoprubart 12638 (129,91), ascuprubart 13837 (134,).*
**229**	**6-G2v95** *chimerisotype G2[4aaG1,3aaG4]*	**2**	*nemolizumab 10064 (112,74), satralizumab 10065 (114/*116*,77/*78*).*
**231**	**10-G2v111**	**nd[c]**	*nemolizumab 10064 (112,74), satralizumab 10065 (114/116,77/78).*
**299***	**15-G2v37-1** *G2[hG1S5],*	(1)	*abituzumab 9509 (109,71).*

	**8,10-G2v36-7**		
**403**	**6-G4v3**	**16**	*inclacumab 9512 (106,68), bleselumab 10114 (113,75) , romilkimab 10622 (118,80), sutimlimab 10737 (118,80), feladilimab 11311 (122,84), bexmarilimab, 11328 (122,84), amlitelimab 11545 (124,86), quisovalimab 12100 (125,87), ebrasodebart 12275 (130,92), riliprubart 12577 (128,90), umikibart 12925 (130,92), efdoralprin alpha* (FPIA) *13115 (131,93), claseprubart 13138 (132,94) tanruprubart 13359 (132,94), latarcibart 13690 (133,95), zamristobart 13722 (133,95).*
**404**	**6-G4v3-1**	**1**	*timolumab 10248 (114,76).*
**405**	**6-G4v3-44**	**1**	*gamgertamig 13797_H (133,95).*
**407**	**6-G4v4**	**30**	*dulaglutide* (CPCA) *9164 (103,65), emibetuzumab 9932 (111,73), galcanezumab 10277 (114,76), mirikizumab 10657 (117,79), frovocimab 10859 (119,81), teclistamab 10928 (120,82), zagotenemab 11050 (121,83), cinrebafusp alfa* (IG) *11094 (121,83), adebrelimab 11300 (122,84), letaplimab 11366 (123,85), torudokimab 11572 (124,86), retlirafusp alfa* (IG) *11662 (124,86), tamgiblimab 11690 (125,87), voxalatamab 11734 (125,87), sirexatamab 11751 (125,87), zansecimab 11797 (124,85), ciduvectamig 12199 (127,89), patecibart 12333 (128,90), fepixnebart 12352 (127,89), etentamig 12522 (130,92), vipalanebart 12534 (129,91), negalstobart 12629 (129,91), lucorafusp alfa* (IG) *12728 (130,92), rezetamig 12767 (129,91), venanprubart 12769 (129,91), efdelikofusp alfa* (FPIA) *13122 (131,93), efzilonkofusp alfa* (FPIA) *13123 (131,93), solabafusp alfa* (IG) *13188 (131,93), surovatamig 13199 (131/132, 93), turigrobart 14005 (134,).*
**408**	**12-G4v5**	**228**	*gemtuzumab 8024 (83,45), inotuzumab ozogamicin 8574 (92,54), dulaglutide* (CPCA) *9164 (103,65), lebrikizumab 9165 (101,63), olokizumab 9333 (103,65), urelumab 9365 (104,66), lirilumab 9415 (107,69), tabalumab 9430 (105,67), blosozumab 9440 (105,67), ixekizumab 9467 (105,67), crenezumab 9482 (105,67), inclakumab 9512 (106,68), fasinumab 9589 (107,69), nivolumab 9623 (107,69), simtuzumab 9626 (107,69), concizumab 9636 (108,70) dupilumab 9669 (108,70), efpegsomatropin* (CPCA) *(114,75), pembrolizumab 9798 (109,72), ulocuplumab 9854 (110,72), fletikumab 9876 (110,72), emibetuzumab 9932 (111,73), evinacumab 10013 (112,74), andecaliximab 10035(115,77), trevogrumab 10087 (113,75), monalizumab 10113 (113,75), bleselumab 10114 (113,75), emicizumab 10115 (113,75), cabiralizumab 10121 (114,76), rinucumab 10175 (113,75), landogrozumab 10188 (113,75), crotedumab 10196 (114,76), rozanolixizumab 10213 (115,77), timolumab 10248 (114,76), galcanezumab 10277 (114,76), gemtuzumab ozogamicin 10315 (115,77), camrelizumab 10400 (115,77), efizonerimod alfa* (FPIA) *10417 (117,79), tislelizumab 10553 (117,79), romilkimab 10622 (118,80), spartalizumab 10624 (117,79), tibulizumab 10656 (117,79), mirikizumab 10657 (117,79), gosuranemab 10663 (119,81), cemiplimab 10691 (119,81), relatlimab 10735 (119,81), sutimlimab 10737 (118,80), cetrelimab 10757 (118,80), dostarlimab 10787 (119,81), cobolimab 10788 (120,82), sintilimab 10801 (119,81), toripalimab 10820 (119,81), orilanolimab 10825 (119,81), temelimab 10830 (119,81)*

			zampilimab 10843 (119,81), frovocimab 10859 (119,81),<br>teclistamab 10928 (120,82), pacmilimab 10938 (121,83),<br>pozelimab 10945 (120,82), axatilimab 10953 (121,83),<br>tilavonemab 10957 (120,82), balstilimab10993 (120,82),<br>garadacimab 10996 (120,82), magrolimab 10999 (120,82),<br>ieramilimab 11005 (120,82), pepinemab 11011 (120,82),<br>nimacimab 11023 (120,82), semorinemab 11024 (120,82),<br>odronextamab 11035 (121,83), garetosmab 11041 (120,82),<br>tomaralimab 11044 (120,82), zagotenemab 11050 (121,83),<br>encelimab 11056 (121,83), cinrebafusp alfa (IG) 11094 (121,83),<br>retifanlimab 11095 (121,83), sasanlimab 11161 (121,83),<br>narsoplimab 11174 (121,83), serplulimab 11176 (121,83),<br>favezelimab 11179 (121,85), fianlimab 11182 (121,83),<br>ongericimab 11188 (122,84), tifcemalimab 11192 (122,86),<br>bepranemab 11224 (122,84), tesnatilimab 11249 (122,84),<br>sabatolimab 11256 (122,84), efmarodocokin alfa (FPIA) 11266 (122,84),<br>adebrelimab 11300 (122,84), miptenalimab 11302 (122,84),<br>ezabenlimab 11303 (122,84), modakafusp alfa (IG) 11306 (122,84),<br>feladilimab 11311 (122,84), bexmarilimab 11328 (122,84),<br>sugemalimab 11330 (122,84), itepekimab 11333 (122,84),<br>tebotelimab 11338 (122,84), imsidolimab 11340 (124,86),<br>vixarelimab 11350 (123,85), letaplimab 11366 (123,85),<br>zimberelimab 11413 (123,85), geptanolimab 11436 (123,85),<br>pimivalimab 11461 (123,85), apitegromab 11471 (123,85),<br>finotonlimab 11492 (124,86), mibavademab 11510 (124,85),<br>lusvertikimab 11533 (124,85), amlitelimab 11545 (124,86),<br>pucotenlimab 11561 (124,86), nadecnemab 11567 (124,86),<br>torudokimab 11572 (124,86), lemzoparlimab 11596 (124,86),<br>ginisortamab 11604 (125,87), xeligekimab 11625 (125,87),<br>retlirafusp alfa (IG) 11662 (124,86), ubamatamab 11679 (125,87),<br>tamgiblimab 11690 (125,87), anumigilimab 11718 (125,87),<br>voxalatamab 11734 (125,87), livmoniplimab 11741 (125,87),<br>sirexatamab 11751 (125,87), lorigerlimab 11771 (125,87)<br>exidavnemab 11775 (125,87), zansecimab 11797(124,85),<br>ligufalimab 11810 (125,87), manfidokimab 11811 (125,87),<br>iparomlimab 11840 (125,87), eblasakimab 11881 (125,87),<br>nofazinlimab 11892 (125,87), acrixolimab 11969 (126,88),<br>enuzovimab 12009 (125,87), visugromab 12044 (126,88),<br>quisovalimab 12100 (125,87), linvoseltamab 12104 (126,88),<br>denecimig 12155 (127,89), ciduvectamig 12199 (127,89),<br>resugosbart 12244 (127,89), trabikibart 12247 (128,90),<br>polzastobart 12249 (127,89), freneslerbart 12255 (127,89),<br>mevonlerbart 12256 (127,89), bremzalerbart 12257 (127,89),<br>umesolerbart 12258 (127,89), atisnolerbart 12259 (127,89),<br>evunzekibart 12262 (127,89), perenostobart 12269 (127,89),<br>ebrasodebart 12275 (130,92), linavonkibart 12283 (127,89),<br>maplirpacept (FPIA) 12295 (127,89), zigakibart 12304 (127,89),<br>varokibart 12309 (127,89), efrilacedase alfa (CPCA) 12312 (126,88),<br>patecibart 12333 (128,90), narlumosbart 12344 (127,89),<br>fepixnebart 12352 (127,89), eltrekibart 12372 (129,91),<br>ucenprubart 12373 (129,91), pradusinstobart 12375 (127,89),<br>lipustobart 12392 (127,89), rademikibart 12401 (128,90),<br>enlonstobart 12413 (128,90), oloctinebart 12421 (128,90),

			stapokibart 12443 (128,90), davutamig 12481 (128,90), vonsetamig 12494 (128,90), etentamig 12522 (130,92) vensobafusp alfa (IG) *12527 (128,90), vipalanebart 12534 (129,91), rolistobart 12567 (128,90), riliprubart 12577 (128,90), gruticibart 12624 (129,91), negalstobart 12629 (129,91), abiprubart 12636 (129, 91), nelmastobart 12640 (129,91), lucorafusp alfa* (IG) *12728 (130,92), roconkibart 12736 (129,91), rezetamig 12767 (129,91), venanprubart 12769 (129,91), reflocibart 12770 (129,91), nezastomig 12804 (129,91), brivestobart 12847 (131,93), zaltenibart 12858 (130,92), comekibart 12879 (130,92), vixticibart 12917 (130,92), imelciment* (IG) *12918 (130,92), umikibart12925 (130,92), marlotamig 13010 (130,94), safimestomig 13102 (131,93), efdoralprin alfa* (FPIA) *13115 (131,93), armocibart 13116 (131,93), birinkibart 13121 (131,93), efdelikofusp alfa* (FPIA) *13122 (131,93), efzilonkofusp alfa* (FPIA) *13123 (131,93), claseprubart 13138 (132,94), alcestobart 13148 (131,93), solabafusp alfa* (IG) *13188 (131,93) surovatamig 13199 (131,93), inclocibart 13212 (133,95), drotokibart 13214 (131,93), mavrostobart 13259 (131,93), zadoprubart 13268 (131,93), eltivutabart 13273 (131,93), sutacimig 13275 (131,93), zemocimig 13346 (132,94), firsekibart 13353 (132,94), tanruprubart 13359(132,94), exerenibart 13405 (132,94), ubletamig 13410 (132,94), cenvacibart 13411 (132,94), amrecibart 13412 (132,94), abazistobart 13430 (132,94), baloncibart 13486 (132,94), oturkibart 13526 (132,94), licastobart 13585 (133,95), ticalicibart 13627 (133,95), ponumkibart 13641 (133,95), gimvekibart 13663 (133,95), olsutamig 13678 (133,95), latarcibart 13690 (133,95), camtarkibart 13699 (133,95), vasigrobart 13706 (133,95), abdakibart 13713 (133,95), zamristobart 13722 (133,95), nebaprubart 13788 (133,95), gamgertamig 13797 (133,95), furtisetabart 13860 (134,), nilvanstomig 13880 (134,), evabosmig 13895 (134,), turigrobart 14005 (134,), radanstobart 14006 (134,).*
**409**	**12-G4v6**	**5**	*emicizumab, 10115 (113,75), tislelizumab 10553 (117,79), safimestomig 13102 (131,93), zemocimig 13346 (132,94), gamgertamig 13797 (133,95).*
**410**	**6-G4v7**	**8**	*odronextamab 11035_M (121,83), fianlimab 11182 (121,83), ubamatamab 11679 (125,87), linvoseltamab 12104 (126,88), vonsetamig 12494 (128,90), nezastomig 12804 (129,91), marlotamig 13010 (130,94), ubletamig 13410 (132,94).*
**412**	**10-G4v8**	**8**	*ubamatamab 11679 (125,87), linvoseltamab 12104 (126,88), davutamig 12481 (128,90), vonsetamig 12494 (128,90), nezastomig 12804 (129,91), imelciment* (IG) *12918 (130,92), marlotamig 13010 (130,94), olsutamig 13678_H (133,95).*
**413**	**10-G4v8-1**	**4**	*lusvertikimab 11533 (124,86), tibulizumab 10056 (117,79), emicizumab, 10115 (113,75), zemocimig 13346 (132).*
**415**	**6,1-G4v9-66**	**1**	*tislelizumab 10553 (117,79).*
**416**	**18-G4v10**	**5**	*teclistamab 10928 (120,82), voxalatamab 11734 (125,87), denecimig 12155 (127,89), ciduvectamig 12199 (127,89), sutacimig 13275 (131,93),*
**417**	**10,9-G4v11**	**3**	*emicizumab 10115 (113,75), safimestomig 13102 (131,93), zemocimig 13346 (132,94).*

**418**	**14-G4v12**	**3**	*emicizumab 10115_H (113,75), safimestomig 13102_H (131,93), zemocimig 13346_F9 (132,94).*
**419**	**14-G4v13**	**3**	*emicizumab 10115_O (113,75), safimestomig 13102_M (131,93), zemocimig 13346_F10 (132).*
**420**	**12-G4v17**	**2**	*clesitamig 13237_H (131,93), evabosmig 13895_M (134,).*
**421**	**9-G4v21**	**6**	*semorinemab 11024 (120,82), tebotelimab, 11338 (122,84), lorigerlimab 11771 (125,87), enuzovimab 12009 (125,87), varokibart 12309 (127,89), claseprubart 13138 (132,94).*
**422**	**9-G4v22**	**1**	*pucotenlimab 11561 (124,86).*
**423**	**9-G4v24**	**5**	*vensobafusp alfa* (IG) *12527 (128,90), riliprubart 12577 (128,90), zaltenibart 12858 (130,92), comekibart 12879 (120,82), latarcibart 13690 (133,95).*
**424**	**8-G4v30**	**1**	*efmarodocokin alfa* (FPIA) *11266 (123,85).*
**425**	**14-G4v32**	**5**	*rezetamig 12767 (129,91), etentamig 12522 (130,92), surovatamig 13199 (131,93), gamgertamig 13797_M (133,95), evabosmig_M 13895 (134,).*
**426**	**14-G4v33**	**5**	*rezetamig 12767 (129,91), etentamig 12522 (130,92), surovatamig 13199 (131,93), gamgertamig 13797_H (133,95), evabosmig_H 13895 (134,).*
**429**	**8-G4v36**	**1**	*vixarelimab 11350 (123,85).*
**430**	**9-G4v42**	**2**	*efdelikofusp alfa* (FPIA) *13122 (131,93), efzilonkofusp alfa* (FPIA) *13123 (131,93).*
**433**	**1-G4v50**	**1**	*olsutamig 13678_H_M (133,95).*
**434**	**17-G4v57-1**	**1**	*zemocimig 13346_F9 (132,94).*
**435**	**17-G4v57-2**	**1**	*zemocimig 13346_F10 (132,94).*
**436**	**17-G4v57-4**	**1**	*evabosmig_M 13895 (134,).*
**439**	**14-G4v74**	**1**	*gamgertamig 13797_M (133,95).*
**440**	**14-G4v75**	**1**	*gamgertamig 13797_H (133,95).*
**441**	**9-G4v78**	**1**	*zemocimig 13346 (132,94).*
**442**	**10-G4v83**	**2**	*odronextamab 11035_M (121,83), evabosmig 13895_H (134,).*
**443**	**10-G4v83-1**	**2**	*emicizumab 10115_H (113,75), safimestomig 13102_H (131,93).*
**444**	**10-G4v89**	**1**	*zemocimig 13346 (132,94).*
**445**	**10-G4v90** *chimerisotype G4[6aaG1]*	**1**	*vixarelimab 11350 (123,85).*
**447**	**10-G4v111**	**nd**[c]	*tibulizumab 10056 (117,79), emicizumab 10115 (113,75), zemocimig 13346 (132).*
**448**	**12-G4v112**	**1**	*clesitamig 13237 (131,93).*
**496***[e]	**12-G4v5, 10-G4v90** *G4[6aaG1]*, **8-G4v36**	(1)	*vixarelimab 11350 (123,85).*
**501**	**17-Kv84-1**	**1**	*linclatamig 13026 (130,92).*
**502**	**17-Kv84-2**	**1**	*linclatamig 13026 (130,92).*
**503**	**17-Kv92-1**	**1**	*spevatamig 13260_L (131,93).*
**504**	**17-Kv92-2**	**1**	*spevatamig 13260_N (131,93).*
**525**	**15-KCv36**	**5**	*naptumomab estafenatox 8598 (96,58), rivabazumab pegol 10144 (113,75), rivabazumab 10197 (114,76), tuvonralimab 11841 (125,87), velaprumig 13342 (132,94).*
**526**	**17-KCv37**	**1**	*spevatamig 13260_L (131,93).*
**527**	**17-KCv57**	**9**	*glofitamab 11145_N (121,83), alnuctamab 11457_N (123,85), englumafusp alfa* (IG) *11793_M (125/*127*,89), trontinemab 12120_L (126,*127*,88), tobemstomig 12318_ N (127/*129*,89/*90*), lomvastomig 12319_N (127,89), forimtamig 12346 (127,89),*

			clesitamig 13237_N (131,93), zareprumig 13958_L (134,). Idem 17-G1v57 (9).
**528**	**17-KCv57-1**	**1**	*zemocimig 13346 (132).*
**529**	**17-KCv57-3**	**1**	*tuvonralimab 11841 (125,87).*
**530**	**17-KCv57-4**	**1**	*evabosmig 13895_N (134,).*
**531**	**17-KCv68**	**1**	*reozalimab 11904 (126,88).*
**532**	**17-KCv84**	**1**	*tuvonralimab 11841 (125,87).*
**533**	**16-KCv93**	**2**	*micvotabart pelidotin 13003 (130,92), micvotabart 13004 (130,92).*
**534**	**16-KCv93-1**	**2**	*zevontabart 13956 (134,), zevontabart vedotin 13957 (134,).*
**535**	**12-KCv96**	**10**	*vanucizumab 9950 (111/113,73), faricimab 10563 (118,80), cibisatamab 10636 (118,80), glofitamab 11145 (121,83), alnuctamab 11457 (123,85), tobemstomig 12318 (127/129,89/90), lomvastomig 12319 (127,89), forimtamig 12346 (127,89) , clesitamig 13237_L (131,93), zareprumig 13958 (134,).*
**536**	**17-KCv100-1**	**1**	*velaprumig 13342_L (132).*
**570**	**12-KVv1**	**1**	*torutamig 13997 (134,).*
**575**	**10-LC2v1**	**1**	*regdanvimab 11883_L (124,85).*
**576**	**17-LC2v57-2**	**1**	*zemocimig 13346_F10 (132).*
**577**	**17-LC2v58**	**5**	*volrustomig 12168 (127,89), sabestomig 12457 (128,90), rilvegostomig 12837 (127,89), tilatamig 13020 (130,92), tilatamig samrotecan 13021 (130,92).*
**579**	**17-LC2v69**	**1**	*reozalimab 11904 (126,88).*
**580**	**10-LC3v1**	**1**	*opucolimab 11310 (122,84)*
**601**	**Canlupfam 6-G2v1**	**3**	*bedinvetmab 11027 (120,82), izenivetmab 11906 (126,88), cirevetmab 11970 (126,88).*
**602**	**Canlupfam 6-G2v2**	**1**	*nolavetbart 12563 (128,90).*
**604**	**Canlupfam 8,6-G2v29-66**	**1**	*gilvetmab 10528 (116,78).*
**651**	**11-scFv-v1**	**13**	*emerfetamab 11239 (123,85), pavurutamab 11290 (123,85), etevritamab 11418 (123,85), tarlatamab 11420 (123,85), acapatamab 11421 (123/124,85), cadonilimab 11581 (124,86), gresonitamab 11798 (125,87), vepsitamab 11822 (125,87), emirodatamab 12028 (126,88), inezetamab 12088 (126,88), galvokimig 12378 (129,91) Fab-2scFv, donzakimig 12679 (129,91) Fab-2scFv, opamtistomig 13330 (132,).*
**652**	**11-scFv-v2**	**8**	*givastomig 12650 (129,91), ragistomig 12707 (129,91), opugotamig 12941 (130,92), opugotamig olatansine 12957 (130,92), ingitamig 12958 (131,93), tovecimig 13128 (131,93), azerutamig 13130 (131,93), nesfrotamig 13381 (132,).*
**653**	**11-scFv-v3**	**1**	*nelvutamig 13774 (133,95).*
**690**	**Homsap 9-Jchain-v1**	**1**	*aplitabart 12990 (130,92).*

[a] ID : IMGT variant identifier.

[b] Nb : number of INN (in July 2026 : up to R95 and PL134 included).

[c] nd : not defined number of variants.

[d] Idem : same number as

[e] * : an asterisk indicates a combination of variants (the number of INN not in bold between parentheses is not counted in the total)
